# Isothiouronium-Mediated Conversion of Carboxylic Acids to Cyanomethyl Thioesters

**DOI:** 10.1021/acs.joc.2c02902

**Published:** 2023-02-28

**Authors:** Irmgard Tiefenbrunner, Bogdan R. Brutiu, Tobias Stopka, Nuno Maulide

**Affiliations:** Institute of Organic Chemistry, University of Vienna, 1090 Vienna Austria

## Abstract

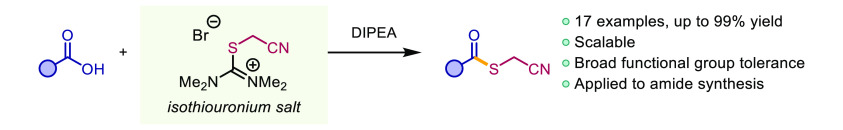

We report the development of an isothiouronium salt as a reagent for the operationally simple synthesis of cyanomethyl thioesters with high functional group tolerance and avoiding the use of thiols. Additionally, we show that the products can be engaged in amide synthesis in either a two-step or one-pot fashion.

Thioesters are a privileged class of organosulfur compounds widely found in a variety of relevant substances for humankind.^1^ In biological systems, thioesters are essential in biosynthetic pathways toward fatty acids, polyketides, and nonribosomal peptides. Perhaps the key thioester in this context is acetyl CoA, which plays a crucial role in the citric acid cycle and thus in ATP production.^2,3^ Moreover, thioesters function as intermediates in the ubiquitination of proteins,^4,5^ leading to protein degradation,^6^ DNA repair,^7^ or cell signaling.^8^ To date, several thioester-containing drugs have been developed, including fluticasone propionate (asthma treatment),^9^ spironolactone (heart failure and hypertension),^10^ and derivatized GW870086 (another fluticasone derivative with anti-inflammatory properties)^11^ (Scheme 1A).

**Scheme 1:**
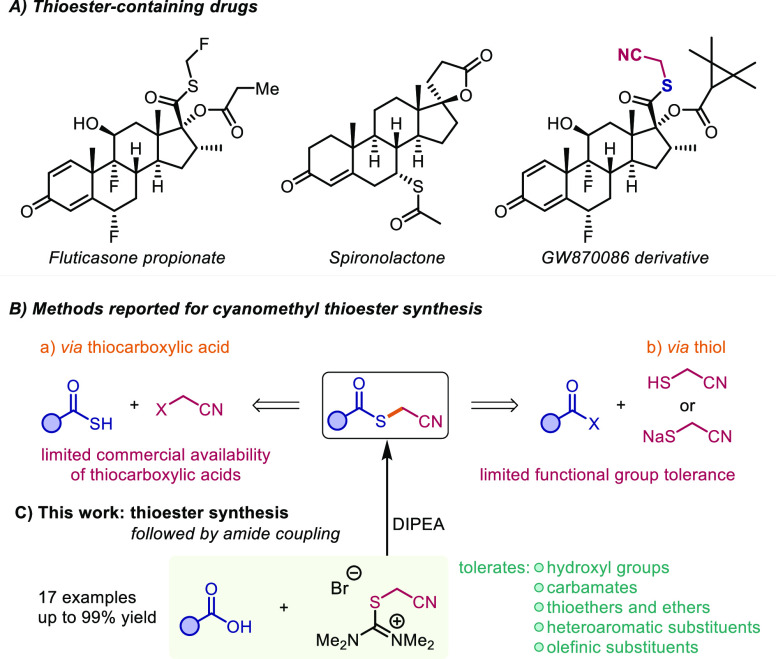
(A) Examples of Thioester-Containing Drugs. (B) Reported Methods for the Synthesis of Cyanomethyl Thioesters. (C) This Work: Cyanomethyl Thioester Synthesis Using Isothiouronium Salts

Due to the high importance of thioesters, considerable work has been devoted to their synthesis. Considering approaches described for, e.g., cyanomethyl thioesters (Scheme 1B), esterification of thiols is the most common strategy, with classical methods relying on the activation of carboxylic acids (via acid chlorides^12^ or anhydrides^13^). Recently, thioester syntheses employing photoredox catalysis have also been developed.^14−20^ Although transition-metal-catalyzed approaches have been described, functional group tolerance remains a challenge in these variants.^21,22^ Thiocarboxylic acids also feature as suitable starting materials for thioester synthesis, either by reaction with an alkyl halide^23−27^ or coupling to a Michael acceptor.^28,29^ Nevertheless, thiols and thiocarboxylic acids suffer from limited commercial availability and are prone to oxidative dimerization (Scheme 1B).^30^

Isothiouronium salts have emerged as valuable reagents for the synthesis of amides^31−35^ and sulfides.^36^ We recently demonstrated that sulfide formation from alcohols can proceed in enantiospecific fashion when isothiouronium salts are deployed.^37^ In the context of thioester formation, Garner and co-workers have successfully reported an air-stable isothiouronium reagent to access 2-pyridinethiol esters.^38^ Despite the high stability of this reagent, its functional group tolerance in the synthesis of thioesters has not been fully delineated. Inspired by these findings, we aimed to investigate isothiouronium salts for the synthesis of thioesters (Scheme 1C). Importantly, isothiouronium salts are air-stable and odorless compounds, readily accessible through alkylation of tetramethylthiourea with a suitable organohalide.^36,37^ This preparation method is typically high-yielding and requires neither specific precautions nor a workup (Scheme 2).

**Scheme 2:**
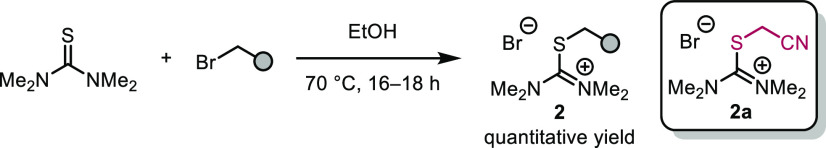
Preparation of Isothiouronium Salts

Several isothiouronium salts were prepared quantitatively using this strategy (see the Supporting Information). Interestingly, among these, only 2-(cyanomethyl)-1,1,3,3-tetramethylisothiouronium bromide (**2a**) was found to productively react with benzoic acid to yield the corresponding thioester (Table 1, see the Supporting Information for full optimization). Initially, reactions were performed in chloroform employing triethylamine as the base. However, under these conditions erratic behavior was observed, likely depending on the quality of the chloroform employed as solvent, with acidity presumably being a contributing factor (Table 1, entries 1 and 2). Therefore, other solvents were screened, revealing acetonitrile as the best choice (entry 3), while apolar solvents such as toluene proved inferior (entry 4). Protic solvents were also shown to enable the transformation but resulted in lower yields (entry 5). Next, different amine bases were evaluated, with DIPEA slightly outperforming NEt_3_ (entry 6). Finally, when 1.5 equiv. of isothiouronium salt **2a** was employed, the desired thioester **3a** was formed in 95% yield (NMR) (entry 7).

**Table 1:**
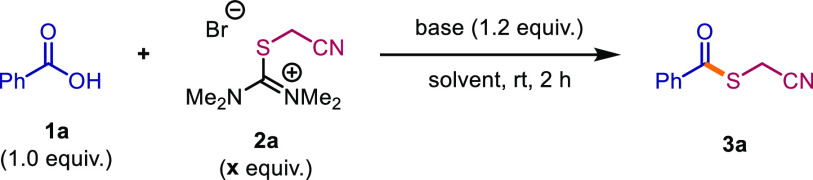
Optimization of Cyanomethyl Thioester Synthesis

entry	base	**2a** (equiv.)	solvent	yield (%)
1	NEt_3_	1.0	CHCl_3_	63
2	NEt_3_	1.0	CHCl_3_^a^	69
3	NEt_3_	1.0	**MeCN**	79
4	NEt_3_	1.0	**toluene**	15
5	NEt_3_	1.0	**iPrOH**	37
6	**DIPEA**	1.0	**MeCN**	83
7	**DIPEA**	**1.5**	**MeCN**	95 (86)^b^

a Filtered through basic aluminum oxide.

b Isolated yield.

With optimized conditions in hand, we studied the impact of varying the carboxylic acid coupling partner (Scheme 3), focusing first on benzoic acid derivatives bearing substituents with different electronic and steric properties. While most substituents were well-tolerated (**3b**, **3c**), *ortho*-substitution led to lower efficiency (**3d**). Electron-withdrawing substituents were also tolerated (**3e**), as were heteroaromatic carboxylic acids (**3f**, **3g**), which gave moderate yields, while acids bearing purely aliphatic substituents (**3h**) performed well in this reaction. Olefinic substituents (**3i**, **3j**) were well tolerated. Notably, an unprotected hydroxyl group did not interfere with thioesterification (**3k**)—an interesting observation, given our recent work on alcohol-to-thioether exchange promoted by isothiouronium salts.^37^ To assess the occurrence of racemization, (*S*)-(+)-2-phenylpropionic acid and Cbz-protected proline were subjected to the reaction conditions, providing products **3l** (90% yield, 97% ee) and **3m** (88% yield, >99% ee) with no observable epimerization. In light of this finding, we further applied our procedure successfully to a diastereomerically pure cyclopropane (**3n**), which also reacted smoothly. Dehydroabietic acid, a particularly hindered substrate, also smoothly underwent conversion to the cyanomethyl thioester derivative **3o**. Moreover, we were pleased to see that the reaction is easily scalable, affording 98% yield of **3a** on a 5 mmol scale. Thus, isothiouronium salt **2a** offers a versatile alternative to procedures described in literature for the synthesis of cyanomethyl thioesters.

**Scheme 3:**
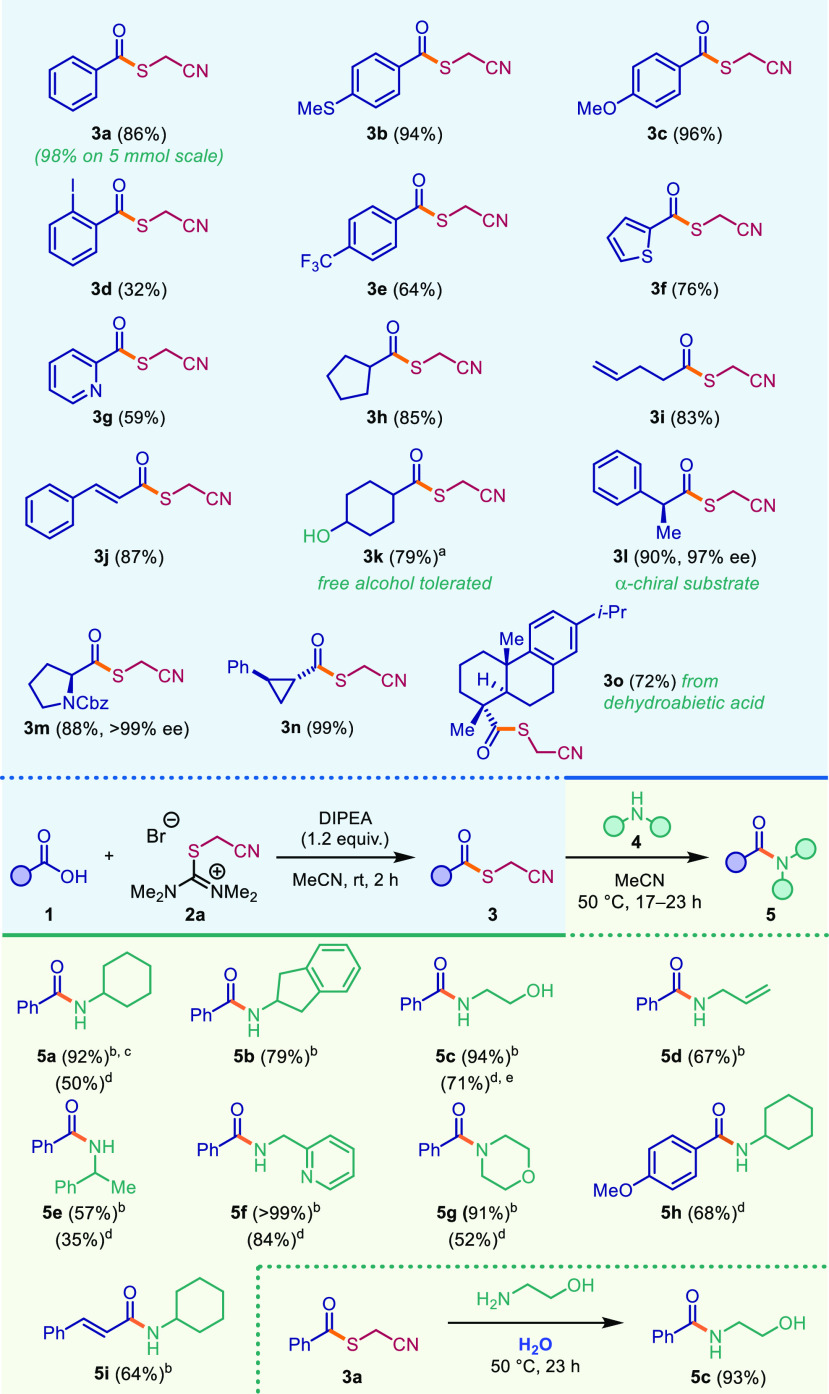
Scope of Thioesters and Amides Mixture of diastereomers. Two-step procedure. Reaction performed at rt. One-pot procedure. 5 equiv. of amine.

We then investigated cyanomethyl thioesters as precursors to amides. In acetonitrile at slightly elevated temperatures, these thioesters readily reacted with a range of amines to provide the corresponding amides. Amines with aliphatic substituents gave excellent yields (**5a**, **5b**), and we once more found that hydroxyl substituents were well tolerated (**5c**). Additionally, allylamine (**5d**), an α-secondary benzylic amine (**5e**), and pyridin-2-ylmethanamine (**5f**) were amenable to amide coupling. Secondary amines are also suitable substrates, as demonstrated by the use of morpholine (**5g**). Additionally, reaction of **3j** with cyclohexylamine gave the desired amide (**5i**) in good yield. Notably, amide **5c** could also be synthesized when water was used as a solvent instead of acetonitrile.

Next, we moved to the development of a one-pot procedure. Herein, after formation of the thioester as described previously, the corresponding amine was directly added to the reaction mixture. We found that, under the same conditions as shown above, the desired amide products were readily obtained (for optimization, see the Supporting Information) in moderate to good yields for this two-step, one-pot process.

The reaction mechanism, in analogy with that reported previously by our group (Scheme 4),^37^ likely entails formation of an intermediate such as **I**. This can subsequently liberate cyanomethylthiolate, forming species **II**, which can then be attacked by a nucleophile at the carbonyl. Two pathways are conceivable: either the thioester is directly formed through addition of cyanomethylthiolate and elimination of tetramethylurea or bromide acts as an intercepting nucleophile, forming a reactive acid bromide **III** prior to substitution by the thiolate.

**Scheme 4:**
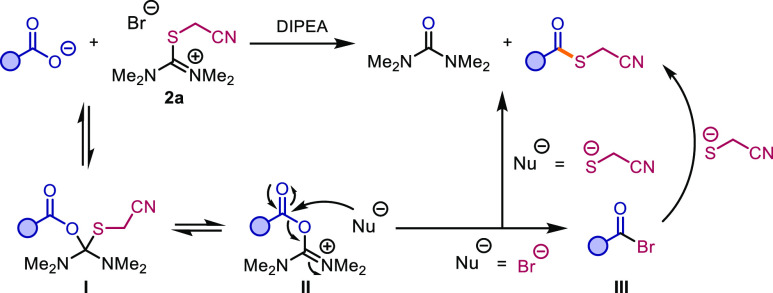
Proposed Mechanism of the Cyanomethyl Thioester Formation

In summary, we have developed easily accessible isothiouronium salt for the synthesis of cyanomethyl thioesters from carboxylic acids. It is worth noting that hydroxyl groups (among other functionalities) are tolerated in a procedure which does not rely on reactants with limited stability such as thiocarboxylic acids and thiols. Notably, both thioester formation as well as amide coupling can be performed without exclusion of air, enabling a particularly facile set up.

## Experimental Section

See this section in the Supporting Information.

## Data Availability

The data underlying this study are available in the published article and its Supporting Information.

## Supporting Information Available

General experimental procedures, NMR spectra and full characterization of all substances not yet described in literature (PDF)
