# Supplementary material for: Isothiouronium-Mediated Conversion of Carboxylic Acids to Cyanomethyl Thioesters
Source: J Org Chem. 2023 Feb 28;88(6):3941–4. doi: 10.1021/acs.joc.2c02902 (PMC10028607; doi:10.1021/acs.joc.2c02902)
Supplement: Supplementary file 1 — jo2c02902_si_001.pdf [file jo2c02902_si_001.pdf]

Supporting Information

Isothiouronium-Mediated Conversion of Carboxylic Acids to Cyanomethyl Thioesters

Irmgard Tiefenbrunner, Bogdan R. Brutiu, Tobias Stopka and Nuno Maulide*

Institute of Organic Chemistry, University of Vienna, Währinger Straße 38, 1090 Vienna (Austria)

E-Mail: nuno.maulide@univie.ac.at

Table of Contents

1. General Information S2

2. Experimental Procedures S3

2.1 General Procedure A: Isothiouronium Salts – Synthesis S3

2.2 Isothiouronium Salts – Characterization S4

2.3 Cyanomethyl Thioester Synthesis – Optimization S7

2.4 General Procedure B: Synthesis of Thioesters S9

2.5 Cyanomethyl Thioesters Characterization. S10

2.6 Two Step Amides Synthesis - Optimization S22

2.7 General Procedure C: Amide Synthesis from Cyanomethyl Esters S23

2.8 Amide Characterization S24

2.9 One-Pot Procedure – Optimization S29

2.10 General Procedure D: One-Pot Amide Synthesis S30

2.11 General Procedure E: Amide Synthesis in Water S33

3. NMR Spectra S34

4. References S63

1. General Information

Unless otherwise stated, all glassware was flame-dried before use and all reactions were performed under an atmosphere of argon. All solvents were distilled from appropriate drying agents prior to use or, if purchased in anhydrous form, used as received. All reagents were used as received from commercial suppliers, unless otherwise stated. Reaction progress was monitored by thin layer chromatography (TLC) performed on aluminum plates coated with silica gel F254 with 0.2 mmthickness. Flash column chromatography was performed using silica gel 60 (230-400 mesh, Merckand co.). Neat infrared spectra were recorded using a Perkin-Elmer Spectrum 100 FT-IR spectrometer. Wavenumbers (vmax) are reported in cm^{-1}. HRESIMS spectra (*m/z* 50-1900) were obtained on a maXis UHR ESI-Qq-TOF mass spectrometer (Bruker Daltonics, Bremen, Germany) in the positive- and/or negative ion mode by direct infusion. The sum formulas of the detected ions were determined using Bruker Compass DataAnalysis 4.1 based on the mass accuracy ($\Delta m/z \leq 5$ ppm) and isotopic pattern matching (SmartFormula algorithm). All ^{1}H NMR, $^{13}C\{^{1}H\}$ NMR, ^{19}F NMR spectra were recorded using a Bruker AV-400, AV-500, AV-600 or AV-700 spectrometer at 300 K. Chemical shifts are given in parts per million (ppm, δ), referenced to the solvent peak of $CDCl_3$, defined at δ = 7.26 ppm (^{1}H NMR) and δ = 77.16 ($^{13}C\{^{1}H\}$ NMR). Coupling constants *J* are quoted in Hz. ^{1}H NMR splitting patterns are designated as singlet (s), doublet (d),triplet (t), quartet (q) as they appeared in the spectrum. If the appearance of a signal differs from the expected splitting pattern, the observed pattern is designated as apparent (app). Splitting patterns thatcould not be interpreted or easily visualized are designated as multiplet (m) or broad (br). Enantiomeric excess was measured on a Shimadzu LC-8A preparative HPLC system using Lux Cellulose-1 or Lux Cellulose-3 chiral columns.

2. Experimental Procedures

2.1 General Procedure A: Isothiouronium Salts – Synthesis

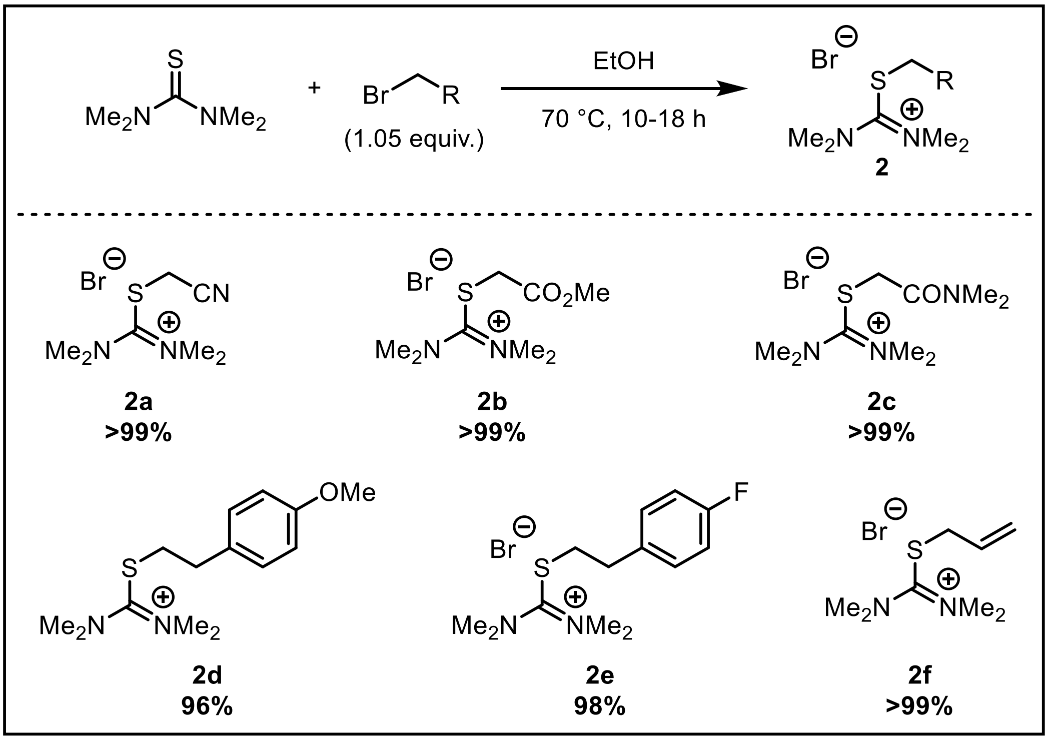

The appropriate alkyl halide (1.05 equiv.) was added to a solution of *N,N,N′,N′*-tetramethylthiourea (TMTU) (1.00 equiv.) in EtOH [1 M]. The mixture was then heated at 70 ˚C for 10 to 18 h. After subsequent cooling to rt, the volatile components of the reaction mixture were removed under reduced pressure to give the desired salt with sufficient purity for further applications.

2.2 Isothiouronium Salts – Characterization

2-(cyanomethyl)-1,1,3,3-tetramethylisothiouronium bromide (2a)

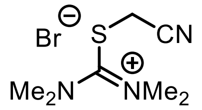

2a

Prepared according to general procedure **A** from TMTU (400 mg, 3.00 mmol, 1.00 equiv.) and bromoacetonitrile (0.22 mL, 3.15 mmol, 1.05 equiv.). The reaction was run for 10 h. White crystalline solid (770 mg, 3.00 mmol, 99% yield). Analytical data in accordance with the literature.[1]

^{1}H NMR (400 MHz, DMSO-d6) δ 4.35 (s, 2H), 3.28 (s, 12H);

^{13}C{^{1}H} NMR (101 MHz, DMSO) δ 171.5, 116.6, 43.8 (4 C), 19.1;

HRMS (ESI) m/z: $[M]^+$ Calcd for $C_7H_{14}N_3S^+$ 172.0903; Found 172.0908;

IR (neat): ν 3392, 2938, 2248, 2076, 1606, 1504, 1461, 1393, 1251, 1204, 1168, 1111, 1056 cm^{-1}.

2-(cyanomethyl)-1,1,3,3-tetramethylisothiouronium bromide (2a) – 5 g scale

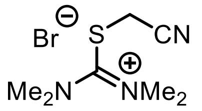

2a

Prepared according to general procedure **A** from TMTU (2.65 g, 20.0 mmol, 1.00 equiv.) and bromoacetonitrile (1.46 ml, 21.0 mmol, 1.05 equiv.). The reaction was run for 18 h. White crystalline solid (5.04 g, 20.0 mmol, >99%).

2-(2-methoxy-2-oxoethyl)-1,1,3,3-tetramethylisothiouronium bromide (2b)

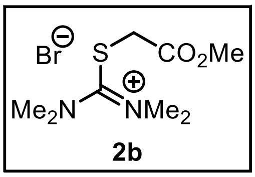

Prepared according to general procedure **A** from TMTU (268 mg, 2.00 mmol, 1.00 equiv.) and methyl bromoacetate (200 µl, 2.10 mmol, 1.05 equiv.) were used. The reaction was run for 18 h. White solid (578 mg, 2.00 mmol, >99% yield).

^{1}H NMR (400 MHz, $CDCl_3$) δ 4.04 (s, 2H), 3.80 (s, 3H), 3.49 (s, 12H);

^{13}C{^{1}H} NMR (101 MHz, $CDCl_3$) δ 168.3, 53.9, 45.0 (4 C), 36.0;

HRMS (ESI) m/z: $[M]^+$ Calcd for $C_8H_{17}N_2O_2S^+$ 205.1011; Found 205.1005;

IR (neat) ν 3423, 2957, 2934, 2923, 1736, 1641, 1605, 1397, 1261, 1111 cm^{-1}.

2-(2-(dimethylamino)-2-oxoethyl)-1,1,3,3-tetramethylisothiouronium bromide (2c)

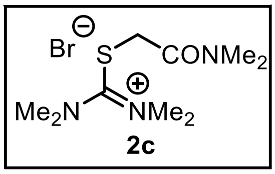

Prepared according to general procedure **A** from TMTU (267 mg, 2.00 mmol, 1.00 equiv.) and 2-bromo-*N*,*N*-dimethylacetamide (238 µl, 2.10 mmol, 1.05 equiv.) were used. The reaction was run for 16 h. White crystalline solid (601 mg, 2.00 mmol, >99% yield).

^{1}H NMR (600 MHz, $CDCl_3$) δ 4.47 (s, 2H), 3.43 (s, 12H), 3.24 (s, 3H), 2.96 (s, 3H);

^{13}C{^{1}H} NMR (151 MHz, $CDCl_3$) δ 176.1, 166.0, 44.7 (4 C), 38.7, 38.6, 36.5;

HRMS (ESI) m/z: $[M]^+$ Calcd for $C_9H_{20}N_3OS^+$ 218.1327; Found 218.1320;

IR (neat) ν 3377, 2936, 1633, 1598, 1393, 1255, 1113 cm^{-1}.

2-(4-methoxyphenethyl)-1,1,3,3-tetramethylisothiouronium bromide (2d)

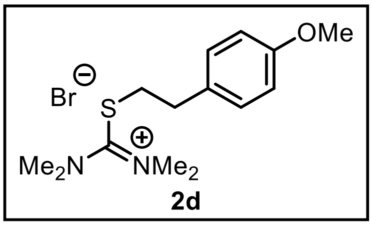

The isothiouronium salt **2d** was prepared in 96% yield (0.96 mmol) according to a reported procedure.[2] Analytical data in accordance with the literature.

2-(4-fluorophenethyl)-1,1,3,3-tetramethylisothiouronium bromide (2e)

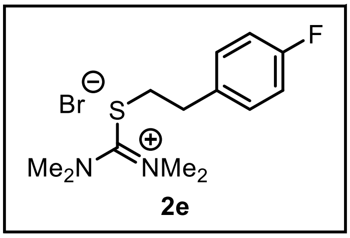

The isothiouronium salt **2e** was prepared in 98% yield (1.96 mmol) according to a reported procedure.[2] Analytical data in accordance with the literature.

2-allyl-1,1,3,3-tetramethylisothiouronium bromide (2f)

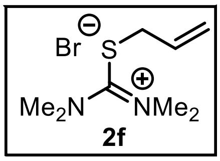

The isothiouronium salt **2f** was prepared in quant yield (1.00 mmol) according to a reported procedure.[2] Analytical data in accordance with the literature.

2.3 Cyanomethyl Thioester Synthesis – Optimization

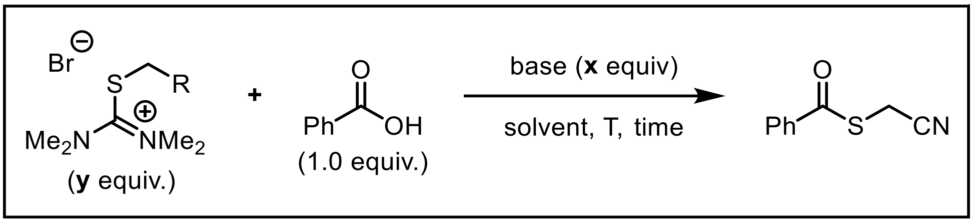

Benzoic acid (0.20 mmol, 1.00 equiv.) and 2-(cyanomethyl)-1,1,3,3-tetramethylisothiouronium bromide (1.00 – 2.00 equiv.) were dissolved in the solvent (2.0 ml, 0.1 M) under air. The base (1.05 – 2.00 equiv.) was added and the resulting mixture was stirred at the given temperature for the given time, followed by quenching by addition of a saturated aqueous solution of NH_4Cl (10 ml). The aqueous layer was extracted with DCM (3x10–15 ml) (after solvent removal when a water miscible solvent was used). The combined organic layers were dried over anhydrous $MgSO_4$, filtered and the solvent was removed under reduced pressure. NMR yields were obtained on the crude mixture, using mesitylene as the internal standard.

entry	base [equiv.]	2a [equiv.]	time [h]	T	solvent	yield [%]	comment
**1**	NEt_3 (1.2)	1.0	2	rt	$CHCl_3$	63	
**2**	**NEt_3 (2.0)**	1.0	2	rt	$CHCl_3$	51	
**3**	**NEt_3 (1.05)**	1.0	2	rt	$CHCl_3$	65	
**4**	NEt_3 (1.2)	1.0	**1**	rt	$CHCl_3$	52	
**5**	NEt_3 (1.2)	**1.2**	2	rt	$CHCl_3$	71	
**6**	NEt_3 (1.2)	1.0	2	**0 °C**	$CHCl_3$	57	
**7**	NEt_3 (1.2)	1.0	2	**60 °C**	$CHCl_3$	59	
**8**	NEt_3 (1.2)	**1.5**	2	rt	$CHCl_3$	81	
**9**	NEt_3 (1.2)	**2.0**	2	rt	$CHCl_3$	84	
**10**	**imidazole (1.2)**	1.0	2	rt	$CHCl_3$	34	
**11**	**2,6-lutidine (1.2)**	1.0	2	rt	$CHCl_3$	19	
**12**	**BTMG (1.2)**	1.0	2	rt	$CHCl_3$	27	
**13**	**DBU (1.2)**	1.0	2	rt	$CHCl_3$	27	
**14**	**TMG (1.2)**	1.0	2	rt	$CHCl_3$	36	
**15**	**DIPEA (1.2)**	1.0	2	rt	$CHCl_3$	67	
**16**	NEt_3 (1.2)	1.0	2	rt	$CHCl_3$	63	Non-anhydrous $CHCl_3$ was used, no filtration
**17**	NEt_3 (1.2)	1.0	2	rt	$CHCl_3$	69	Non-anhydrous $CHCl_3$ was used, filtration over basic aluminium oxide
**18**	NEt_3 (1.2)	1.0	2	rt	**DCM**	73	
**19**	NEt_3 (1.2)	1.0	2	rt	**DCE**	66	
**20**	NEt_3 (1.2)	1.0	2	rt	**DMF**	63	
**21**	NEt_3 (1.2)	1.0	2	rt	**MeCN**	79	
**22**	NEt_3 (1.2)	1.0	2	rt	**iPrOH**	37	
**23**	NEt_3 (1.2)	1.0	2	rt	**THF**	22	
**24**	NEt_3 (1.2)	1.0	2	rt	**toluene**	15	
**25**	**DIPEA (1.2)**	1.0	2	rt	**MeCN**	83	
**26**	**DIPEA (1.2)**	**1.5**	2	rt	**MeCN**	95 [86]	

[a] Reactions conducted on a 0.2 mmol scale. Yields refer to NMR yields. Isolated yields in brackets; BTMG (2-*tert*-butyl-1,1,3,3-tetramethylguanidine), DBU (1,8-diazabicyclo(5.4.0)undec-7-ene), TMG (1,1,3,3-tetramethylguanidine), DIPEA (*N,N*-diisopropylethylamine)

2.4 General Procedure B: Synthesis of Thioesters

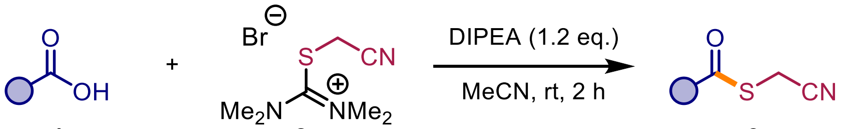

1 + **2a** → **3**

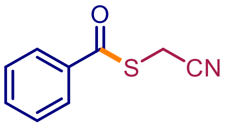

3a
(86%)
(98% on 5 mmol scale)

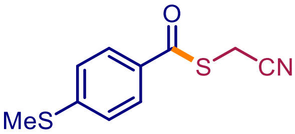

3b
(94%)

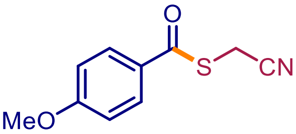

3c
(96%)

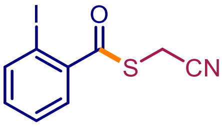

3d
(32%)

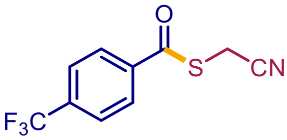

3e
(64%)

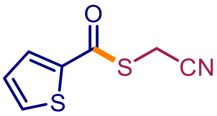

3f
(76%)

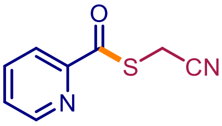

3g
(59%)

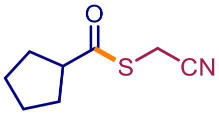

3h
(85%)

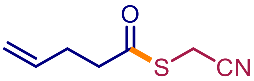

3i
(83%)

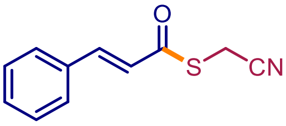

3j
(87%)

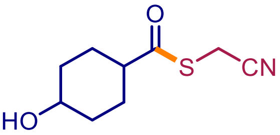

3k
(79%)[a]

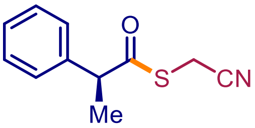

3l
(90%, 97% ee)

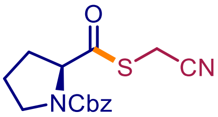

3m
(88%, >99% ee)

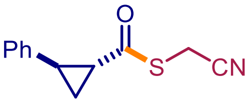

3n
(99%)

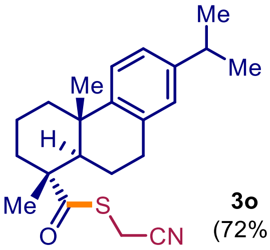

3o
(72%)

limitations:

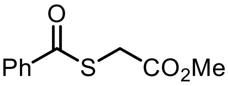

3p
<5% conversion

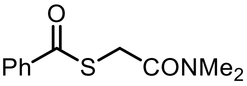

3q
no product formation

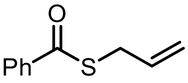

3r
<5% conversion

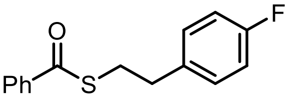

3s
<10% conversion

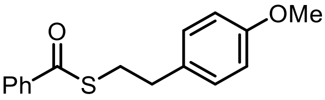

3t
<5% conversion

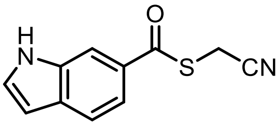

3u
72% NMR yield, difficult purification

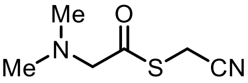

3v
no conversion, recovered starting material

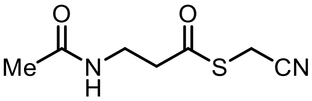

3x
<5% NMR yield

The carboxylic acid (0.20 mmol, 1.00 equiv.) and **2a** (0.30 mmol, 1.00 equiv.) were dissolved in MeCN (2.0 ml, 0.1 M) under air. DIPEA (0.24 mmol, 1.2 equiv.) was added and the resulting mixture was stirred at rt for 2 h, followed by quenching by addition of a saturated aqueous solution of NH_4Cl (10 ml). MeCN was removed under reduced pressure. The aqueous layer was extracted with DCM (3x10-15 ml). The combined organic layers were dried over $MgSO_4$, filtered and the solvent was removed under reduced pressure. The residue was purified by column chromatography.

2.5 Cyanomethyl Thioesters Characterization.

_S_-(cyanomethyl) benzothioate (3a)

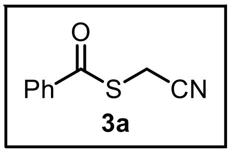

Prepared according to general procedure **B** from benzoic acid (24.8 mg, 0.20 mmol, 1.00 equiv.) and **2a** (75.7 mg, 0.30 mmol, 1.50 equiv.). Purification on silica gel (heptane/EtOAc = 9:1). Colorless oil (31.2 mg, 0.18 mmol, 86% yield). Analytical data in accordance with the literature.[3]

^{1}H NMR (400 MHz, $CDCl_3$) δ 7.97 – 7.89 (m, 2H), 7.65 (t, *J* = 7.5 Hz, 1H), 7.50 (t, *J* = 7.8 Hz, 2H), 3.86 (s, 2H);

^{13}C{^{1}H} NMR (101 MHz, $CDCl_3$) δ 188.0, 135.4, 134.7, 129.2 (2 C), 127.7 (2 C), 116.0, 14.5;

HRMS (ESI) m/z: $[M + Na]^+$ Calcd for $C_9H_7NOSNa^+$ 200.0141; Found 200.0141;

IR (neat): ν 2922, 2852, 2250, 1671, 1595, 1581, 1448, 1382, 1207, 1177, 904, 850, 772, 730, 683, 645, 616 cm^{-1}.

_S_-(cyanomethyl) benzothioate (3a) – 5 mmol scale

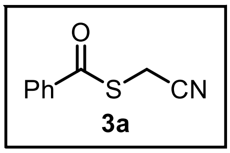

Prepared according to general procedure **B** from benzoic acid (619 mg, 5.10 mmol, 1.00 equiv.), **2a** (1.90 g, 7.50 mmol, 1.50 equiv.) and DIPEA (1.05 ml, 6.00 mmol, 1.20 equiv.). 50 ml of MeCN were used. Purification on silica gel (heptane/EtOAc = 9:1). Colorless solid (878 mg, 5.00 mmol, 98% yield).

***S*-(cyanomethyl) 4-(methylthio)benzothioate (3b)**

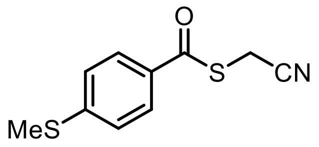

3b

Prepared according to general procedure **B** from 4-(methylthio)benzoic acid (35.2 mg, 0.21 mmol, 1.00 equiv.) and **2a** (75.7 mg, 0.30 mmol, 1.50 equiv.). Purification on silica gel (heptane/EtOAc = 12:1 to 9:1). White solid (43.7 mg, 0.20 mmol, 94% yield).

^{1}H NMR (600 MHz, $CDCl_3$) δ 7.87 – 7.77 (dt, *J* = 8.6, 1.9 Hz, 2H), 7.31 – 7.23 (dt, *J* = 8.6, 1.9 Hz, 2H), 3.85 (s, 2H), 2.53 (s, 3H);

^{13}C{^{1}H} NMR (151 MHz, $CDCl_3$) δ 186.8, 148.3, 131.3, 127.9 (2C), 125.2 (2C), 116.1, 14.8, 14.4;

HRMS (ESI) m/z: $[M + Na]^+$ Calcd for $C_{10}H_9NOS_2Na^+$ 246.0023; Found 246.0015;

IR (neat) ν 2987, 2942, 2922, 2247, 1671, 1588, 1180, 741 cm^{-1}.

***S*-(cyanomethyl) 4-methoxybenzothioate (3c)**

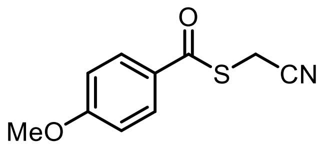

3c

Prepared according to general procedure **B** from *p*-anisic acid (30.3 mg, 0.20 mmol, 1.00 equiv.) and **2a** (75.7 mg, 0.30 mmol, 1.50 equiv.). Purification on silica gel (heptane/EtOAc = 9:1 to 5:1). White solid (39.5 mg, 0.19 mmol, 96% yield).

^{1}H NMR (600 MHz, $CDCl_3$) δ 7.94 – 7.86 (m, 2H), 6.95 (d, *J* = 8.9 Hz, 2H), 3.88 (s, 3H), 3.84 (s, 2H);

^{13}C{^{1}H} NMR (151 MHz, $CDCl_3$) δ 186.3, 164.8, 130.0 (2C), 128.0, 116.2, 114.3 (2C), 55.8, 14.3;

HRMS (ESI) m/z: $[M + Na]^+$ Calcd for $C_{10}H_9NO_2SNa^+$ 230.0252; Found 230.0246;

IR (neat) ν 2985, 2939, 2844, 2250, 1669, 1601, 1264, 1170, 912 cm^{-1}.

S-(cyanomethyl) 2-iodobenzothioate (3d)

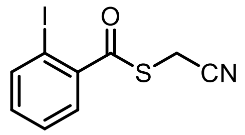

Prepared according to general procedure **B** from 2-Iodobenzoic acid (49.4 mg, 0.20 mmol, 1.00 equiv.) and **2a** (75.7 mg, 0.30 mmol, 1.50 equiv.). Purification on silica gel (heptane/EtOAc = 9:1 to 2:1). Orange oil (19.4 mg, 0.06 mmol, 32% yield).

^{1}H NMR (400 MHz, $CDCl_3$) δ 8.04 (d, *J* = 7.9 Hz, 1H), 7.68 (d, *J* = 7.7 Hz, 1H), 7.51 (t, *J* = 7.6 Hz, 1H), 7.29 (d, *J* = 9.9 Hz, 1H), 3.93 (s, 2H);

$^{13}C\{^1H\}$ NMR (101 MHz, $CDCl_3$) δ 189.8, 141.6, 140.4, 133.6, 129.3, 128.4, 115.5, 91.7, 15.5;

HRMS (ESI) m/z: $[M + Na]^+$ Calcd for $C_9H_6INOSNa^+$ 325.9113; Found 325.9104;

IR (neat) ν 2979, 2924, 2851, 2250, 1685, 1577, 1458, 1430, 1383, 1281, 1266, 1205, 1047, 1019, 906, 764 cm^{-1}.

S-(cyanomethyl) 4-(trifluoromethyl)benzothioate (3e)

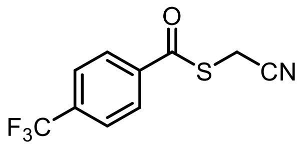

Prepared according to general procedure **B** from 4-(trifluoromethyl)benzoic acid (38.8 mg, 0.20 mmol, 1.00 equiv.) and **2a** (75.7 mg, 0.30 mmol, 1.00 equiv.). Purification on silica gel (Heptane/EtOAc = 2:1). Colorless oil (31.5 mg, 0.13 mmol, 64% yield).

^{1}H NMR (400 MHz, $CDCl_3$): δ 8.06 (d, *J* = 8.2 Hz, 2H), 7.78 (d, *J* = 8.3 Hz, 2H), 3.90 (s, 2H);

$^{13}C\{^1H\}$ NMR (101 MHz, $CDCl_3$): δ 187.4, 138.1, 135.94 (q, *J* = 32.8 Hz), 128.1 (2C), 126.3 (2C, q, *J* = 3.7 Hz), 123.4 (q, *J* = 273.0 Hz), 115.5, 14.8;

^{18}F NMR (376 MHz, $CDCl_3$): δ -63.3;

HRMS (ESI) m/z: $[M + Na]^+$ Calcd for $C_{10}H_6F_3NOSNa^+$: 268.0014 found: 268.0016;

IR (neat): ν 2987, 2935, 2251, 1675, 1584, 1410, 1387, 1324, 1267, 1211, 1173, 1130, 1113, 1066, 1016, 917, 851, 775 cm^{-1}.

S-(cyanomethyl) thiophene-2-carbothioate (3f)

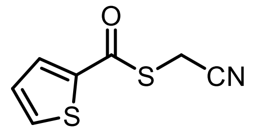

3f

Prepared according to general procedure **B** from thiophene-2-carboxylic acid (25.6 mg, 0.20 mmol, 1.00 equiv.) and **2a** (75.7 mg, 0.30 mmol, 1.00 equiv.). Purification on silica gel (Heptane/EtOAc = 3:1). Colorless oil (27.7 mg, 0.15 mmol, 76% yield).

^{1}H NMR (400 MHz, $CDCl_3$) δ 7.83 (dd, *J* = 3.9, 1.2 Hz, 1H), 7.74 (dd, *J* = 4.9, 1.2 Hz, 1H), 7.17 (dd, *J* = 4.9, 3.9 Hz, 1H), 3.87 (s, 2H);

$^{13}C\{^1H\}$ NMR (101 MHz, $CDCl_3$) δ 179.8, 139.9, 134.8, 132.7, 128.6, 115.9, 14.6;

HRMS (ESI) m/z: $[M + H]^+$ Calcd for $C_7H_6NOS_2^+$ 183.9885; Found 183.9881;

IR (neat) v. 2930; 2250, 1688, 1263, 1121, 926, 745, 702, 619 cm^{-1}.

S-(cyanomethyl) pyridine-2-carbothioate (3g)

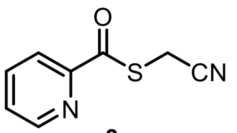

3g

Prepared according to general procedure **B** from pyridine-2-carboxylic acid (24.6 mg, 0.20 mmol, 1.00 equiv.) and **2a** (75.7 mg, 0.30 mmol, 1.00 equiv.). Purification on silica gel (Heptane/EtOAc = 3:1). Colorless oil (21.1 mg, 0.12 mmol, 59% yield).

^{1}H-NMR (400 MHz, $CDCl_3$): δ 8.69 (ddd, *J* = 4.7, 1.5, 0.9 Hz, 1H), 8.05 – 7.95 (m, 1H), 7.90 (td, *J* = 7.7, 1.6 Hz, 1H), 7.58 (ddd, *J* = 7.5, 4.7, 1.2 Hz, 1H), 3.77 (s, 2H);

$^{13}C\{^1H\}$ NMR (101 MHz, $CDCl_3$): δ 190.8, 150.4, 149.5, 137.7, 128.9, 121.0, 116.3, 14.4;

HRMS (ESI) m/z: $[M + Na]^+$ Calcd for $C_8H_6N_2OSNa^+$: 201,0093 found: 201.0095;

IR (neat): v 1687, 1266, 1221, 924, 731, 705, 645, 617 cm^{-1}.

_S_-(cyanomethyl) cyclopentanecarbothioate (3h)

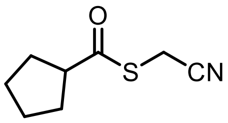

3h

Prepared according to general procedure **B** from cyclopentane carboxylic acid (21.7 µl, 0.20 mmol, 1.00 equiv.) and **2a** (75.7 mg, 0.30 mmol, 1.50 equiv.). Purification on silica gel (heptane/EtOAc = 9:1 to 7:1). Orange oil (28.7 mg, 0.17 mmol, 85% yield).

^{1}H NMR (400 MHz, $CDCl_3$) δ 3.66 (s, 2H), 3.02 (p, *J* = 7.9 Hz, 1H), 1.98 – 1.91 (m, 2H), 1.89 – 1.80 (m, 2H), 1.79 – 1.67 (m, 2H), 1.68 – 1.55 (m, 2H);

^{13}C{^{1}H} NMR (151 MHz, $CDCl_3$) δ 199.0, 116.1, 53.0, 30.5 (2C), 25.9 (2C), 14.2;

HRMS (ESI) m/z: $[M + Na]^+$ Calcd for $C_8H_{11}NOSNa^+$ 192.0459; Found 192.0453;

IR (neat) ν 3324, 2960, 2923, 2871, 2852, 2250, 2208, 1701, 1527, 938, 869, 703 cm^{-1}.

_S_-(cyanomethyl) pent-4-enethioate (3i)

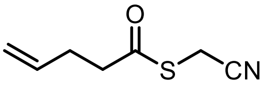

3i

Prepared according to general procedure **B** from 4-pentenoic acid (20.5 µl, 0.20 mmol, 1.00 equiv.) and **2a** (75.7 mg, 0.30 mmol, 1.50 equiv.). Purification on silica gel (heptane/EtOAc = 15:1 to 9:1). Yellow oil (25.9 mg, 0.17 mmol, 83% yield).

^{1}H NMR (600 MHz, $CDCl_3$) δ 5.78 (ddt, *J* = 16.8, 10.2, 6.5 Hz, 1H), 5.08 (dd, *J* = 17.1, 1.2 Hz, 1H), 5.05 (d, *J* = 10.2 Hz, 1H), 3.68 (s, 2H), 2.74 (t, *J* = 7.4 Hz, 2H), 2.45 (q, *J* = 7.0 Hz, 2H);

^{13}C{^{1}H} NMR (151 MHz, $CDCl_3$) δ 194.8, 135.4, 116.7, 115.9, 42.8, 29.1, 14.3;

HRMS (ESI) m/z: $[M + Na]^+$ Calcd for $C_7H_9NOSNa^+$ 178.0303; Found 178.0299;

IR (neat) ν 3324, 2920, 2851, 2206, 1680, 1462, 912, 741 cm^{-1}.

S-(cyanomethyl) (E)-3-phenylprop-2-enethioate (3j)

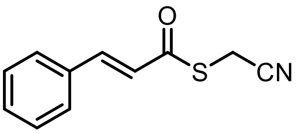

3j

Prepared according to general procedure **B** from trans-cinnamic acid (29.9 mg, 0.20 mmol, 1.00 equiv.) and **2a** (75.7 mg, 0.30 mmol, 1.5 equiv.). Purification on silica gel (heptane/EtOAc = 16:1 to 5:1). White solid (35.6 mg, 0.18 mmol, 87% yield). Analytical data in accordance with literature.[4]

^{1}H NMR (700 MHz, $CDCl_3$) δ 7.69 (d, J = 15.8 Hz, 1H), 7.58 – 7.54 (m, 2H), 7.47 – 7.39 (m, 3H), 6.72 (d, J = 15.8 Hz, 1H), 3.81 (s, 2H);

^{13}C{^{1}H} NMR (176 MHz, $CDCl_3$) δ 185.7, 143.7, 133.5, 131.5, 129.2 (2C), 128.8 (2C), 122.9, 116.0, 14.3;

HRMS (ESI) m/z: $[M + Na]^+$ Calcd for $C_{11}H_9NOSNa^+$ 226.0303; Found 226.0299;

IR (neat) ν 3324, 3063, 3031, 3006, 2952, 2923, 2849, 2328, 2207, 1737, 1267, 1136, 742 cm^{-1}.

S-(cyanomethyl) 4-hydroxycyclohexane-1-carbothioate (3k)

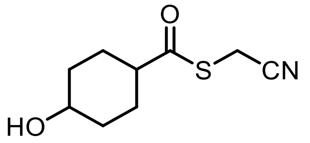

3k

Prepared according to general procedure **B** from 4-hydroxycyclohexane-1-carboxylic acid (cis, trans mixture, 29.5 mg, 0.20 mmol, 1.00 equiv.) and **2a** (75.7 mg, 0.30 mmol, 1.50 equiv.). Purification on silica gel (heptane/EtOAc = 7:1 to 1:1). Pale yellow oil (28.5 mg, 0.14 mmol, 70% yield of one isomer and 3.6 mg, 0.02 mmol, 9% yield of the other isomer and 8.6 mg, 0.04 mmol, 21% yield of a 1:1 mixture of both isomers).

Major isomer:

^{1}H NMR (600 MHz, $CDCl_3$) δ 3.98 – 3.94 (br, 1H), 3.66 (s, 2H), 2.60 (tt, J = 9.7, 3.7 Hz, 1H), 2.05 – 1.93 (m, 2H), 1.80 – 1.70 (m, 4H), 1.67 – 1.58 (m, 2H);

^{13}C{^{1}H} NMR (151 MHz, $CDCl_3$) δ 198.5, 116.1, 65.9, 50.8, 31.9 (2C), 23.7 (2C), 14.0;

HRMS (ESI) m/z: $[M + Na]^+$ Calcd for $C_9H_{13}NO_2SNa^+$ 222.0565; Found 222.0560;

IR (neat) ν 3396, 2934, 2862, 2251, 1700, 962 cm^{-1}.

Minor isomer:

^{1}H NMR (600 MHz, $CDCl_3$) δ 3.66 (s, 2H), 3.66 – 3.60 (m, 1H), 2.52 (tt, *J* = 11.7, 3.5 Hz, 1H), 2.06 (ddd, *J* = 12.6, 8.8, 3.3 Hz, 4H), 1.64 – 1.52 (m, 2H), 1.39 – 1.28 (m, 2H);

^{13}C{^{1}H} NMR (151 MHz, $CDCl_3$) δ 198.4, 116.0, 69.5, 51.2, 34.3 (2C), 27.5 (2C), 14.1.

HRMS (ESI) m/z: $[M + Na]^+$ Calcd for $C_9H_{13}NO_2SNa^+$ 222.0565; Found 222.0558;

IR (neat) ν 3323, 2924, 2853, 2360, 1713, 1677, 1453, 1136, 740 cm^{-1}.

***S*-(cyanomethyl) 2-phenylpropanethioate (rac-3l)**

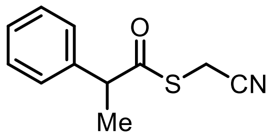

rac-3l

Prepared according to general procedure **B** from racemic phenylpropionic acid (32.2 mg, 0.21 mmol, 1.00 equiv.) and **2a** (75.7 mg, 0.30 mmol, 1.4 equiv.). Purification on silica gel (heptane/EtOAc = 15:1 to 9:1). Colourless oil (38.5 mg, 0.19 mmol, 87% yield).

^{1}H NMR (600 MHz, $CDCl_3$) δ 7.41 – 7.28 (m, 5H), 3.93 (q, *J* = 7.1 Hz, 1H), 3.63 (d, *J* = 16.8 Hz, 1H), 3.56 (d, *J* = 16.8 Hz, 1H), 1.60 (d, *J* = 7.1 Hz, 3H);

^{13}C{^{1}H} NMR (151 MHz, $CDCl_3$) δ 197.7, 138.3, 129.1 (2 C), 128.3 (2 C), 128.3, 115.9, 54.1, 18.1, 14.5;

HRMS (ESI) m/z: $[M + Na]^+$ Calcd for $C_{11}H_{11}NOSNa^+$ 228.0459; Found 228.0455;

IR (neat) ν 3320, 3064, 3030, 2980, 2935, 2250, 1698, 1494, 1453, 944, 740, 701 cm^{-1}.

S-(cyanomethyl) (*S*)-2-phenylpropanethioate (3l)

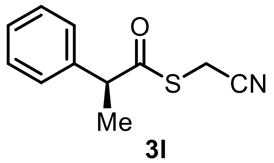

3l

Prepared according to general procedure **B** from (S)-(+)-2-phenylpropionic acid (30.7 mg, 0.20 mmol, 1.00 equiv.) and **2a** (75.7 mg, 0.30 mmol, 1.50 equiv.). Purification on silica gel (heptane/EtOAc = 15:1 to 9:1). Colorless oil (37.9 mg, 0.18 mmol, 90% yield).

HPLC: *ee*: 97%

Racemic standard

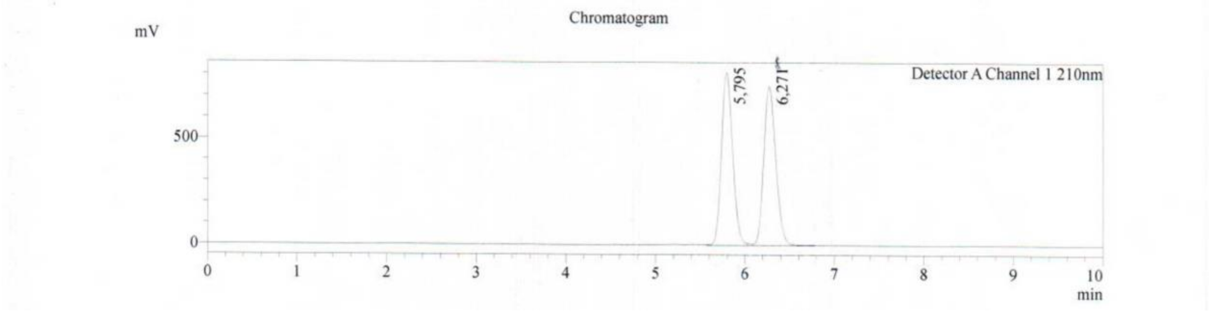

Method Description:
Column:Chiralpak IH-3 150x4,6mm
Solvent System: n-Heptan+0,1%IPA/IPA 8:2
Flow:1 ml/min
T=25°C

Detector A Channel 1 210nm

Peak#	Ret. Time	Area	Area%
1	5,795	7058788	50,130
2	6,271	7022256	49,870
Total		14081043	100,000

3k:

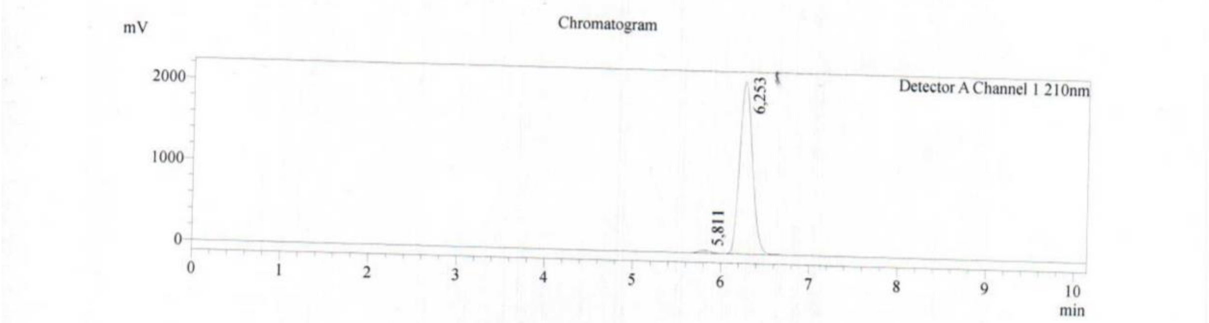

Method Description:
Column:Chiralpak IH-3 150x4,6mm
Solvent System: n-Heptan+0,1%IPA/IPA 8:2
Flow:1 ml/min
T=25°C

Detector A Channel 1 210nm

Peak#	Ret. Time	Area	Area%
1	5,811	308644	1,412
2	6,253	21553585	98,588
Total		21862228	100,000

benzyl (*S*)-2-(((cyanomethyl)thio)carbonyl)pyrrolidine-1-carboxylate (3m)

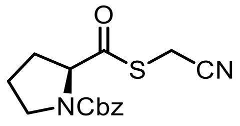

3m

Prepared according to general procedure **B** from Cbz-proline (50.6 mg, 0.20 mmol, 1.00 equiv.) and **2a** (75.7 mg, 0.30 mmol, 1.50 equiv.). Purification on silica gel (DCM/MeOH = 100:0 to 100:1). Orange oil (53.6 mg, 0.18 mmol, 88% yield).

^{1}H NMR (600 MHz, $CDCl_3$) δ rotamers 7.41 – 7.28 (m, 5H), 5.18$_a$ (s, 2H), 5.09$_b$ (d, *J* = 12.2 Hz, 2H), 4.57 (dd, *J* = 40.9, 7.2 Hz, 1H), 3.74 – 3.40 (m, 4H), 2.33 – 2.16 (m, 1H), 2.10 (s, 1H), 2.04 – 1.86 (m, 2H);

$^{13}C\{^1H\}$ NMR (151 MHz, $CDCl_3$) δ rotamers (1:1 mixture) 199.4, 198.9, 155.5, 154.5, 136.3, 136.0, 128.6, 128.3, 128.3, 128.0, 115.9, 115.8, 67.7, 66.4, 65.9, 47.4, 47.1, 31.6, 30.6, 24.3, 23.5, 14.3, 14.1;

HRMS (ESI) m/z: $[M + Na]^+$ Calcd for $C_{15}H_{16}N_2O_3SNa^+$ 327.0779; Found 327.0765;

IR (neat) ν 3033, 2981, 2929, 2879, 2250, 1712, 1401, 1358 cm^{-1}.

benzyl 2-(((cyanomethyl)thio)carbonyl)pyrrolidine-1-carboxylate (rac-3m)

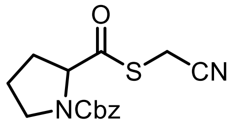

rac-3m

Prepared according to general procedure **B** from (Z-DL-proline (50.6 mg, 0.20 mmol, 1.00 equiv.) and **2a** (75.7 mg, 0.30 mmol, 1.50 equiv.). Purification on silica gel (DCM/MeOH = 100:0 to 100:1). Orange oil (37.9 mg, 0.13 mmol, 62% yield).

^{1}H NMR (400 MHz, $CDCl_3$) δ 7.43 – 7.28 (m, 5H), 5.24 – 5.03 (m, 2H), 4.58 (dd, J = 26.7, 7.9 Hz, 1H), 3.77 – 3.41 (m, 4H), 2.34 – 2.18 (m, 1H), 2.12 (br s, 1H), 1.97 (br s, 2H).

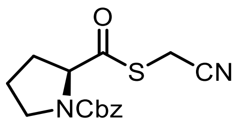

3m

HPLC: *ee*: >99%

Racemic standard (purchased from ABCR):

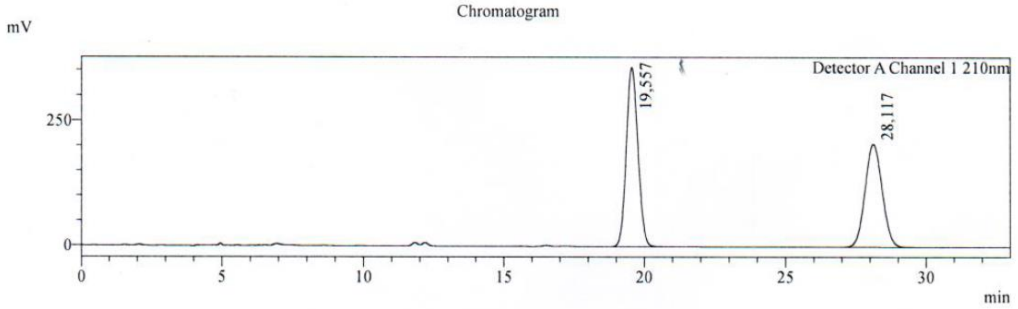

Detector A Channel 1 210nm

Peak#	Ret. Time	Area	Area%
1	19,557	10262069	53,943
2	28,117	8761798	46,057
Total		19023867	100,000

Detector A Channel 2 230nm

Peak#	Ret. Time	Area	Area%
1	19,559	2726967	54,024
2	28,118	2320742	45,976
Total		5047709	100,000

Method Description:
Column:Lux-Cellulose1 (Chiralcel OD-H) 250x4,6mm
Particle Size 5 micrometer
Solvent System: n-Heptan+0,1%IPA/IPA 7:3
Flow:0,7 ml/min

3l:

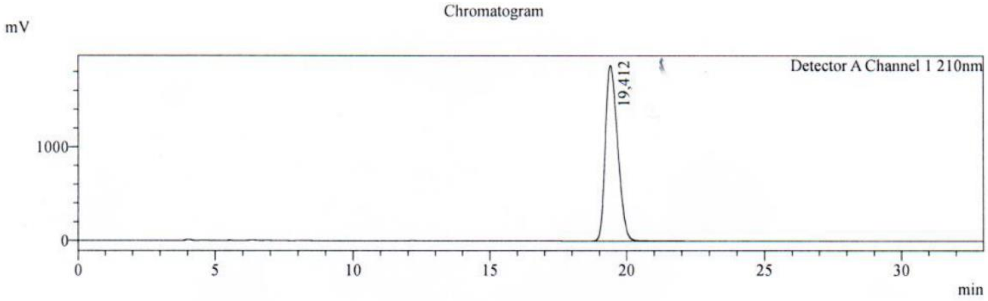

Detector A Channel 1 210nm

Peak#	Ret. Time	Area	Area%
1	19,412	58704496	100,000
Total		58704496	100,000

Detector A Channel 2 230nm

Peak#	Ret. Time	Area	Area%
1	19,412	16438744	100,000
Total		16438744	100,000

Method Description:
Column:Lux-Cellulose1 (Chiralcel OD-H) 250x4,6mm
Particle Size 5 micrometer
Solvent System: n-Heptan+0,1%IPA/IPA 7:3
Flow:0,7 ml/min

***trans S*-(cyanomethyl)-2-phenylcyclopropane-1-carbothioate (3n)**

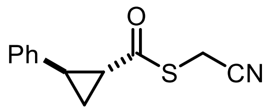

3n

Prepared according to general procedure **B** from *trans* 2-phenyl-1-cyclopropanecarboxylic acid (32.4 mg, 0.20 mmol, 1.00 equiv.) and **2a** (75.7 mg, 0.30 mmol, 1.00 equiv.)**.** Purification on silica gel (Heptane/EtOAc = 5:1). Colorless oil (43.0 mg, 0.20 mmol, 99% yield).

^{1}H-NMR (400 MHz, $CDCl_3$): δ 7.27 – 7.20 (m, 2H), 7.16 (ddd, *J* = 7.4, 3.8, 1.3 Hz, 1H), 7.07 – 7.00 (m, 2H), 3.64 (d, *J* = 1.4 Hz, 2H), 2.70 (ddd, *J* = 9.4, 6.9, 4.0 Hz, 1H), 2.18 (ddd, *J* = 8.2, 5.1, 4.1 Hz, 1H), 1.80 (dt, *J* = 9.7, 5.0 Hz, 1H), 1.50 (ddd, *J* = 8.1, 7.0, 4.8 Hz, 1H);

^{13}C{^{1}H} NMR (101 MHz, $CDCl_3$): δ 193.6, 138.9, 128.8 (2C), 127.2, 126.4 (2C), 33.5, 29.8, 19.6, 14.4;

HRMS (ESI) m/z: $[M + Na]^+$ Calcd for $C_{12}H_{11}NOSNa^+$: 240.0454 found: 240.0454;

IR (neat): ν 1690, 1264, 1031, 986, 731, 699 cm^{-1}.

***S*-(cyanomethyl) (1*R*,4*aS,*10*aR*)-7-isopropyl-1,4a-dimethyl-1,2,3,4,4a,9,10,10a-octahydrophen-anthrene-1-carbothioate (3o)**

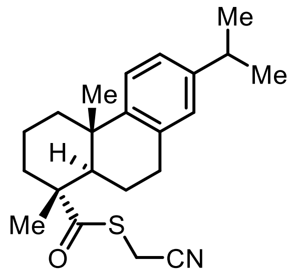

3o

Prepared according to general procedure **B** from dehydroabietic acid (60.1 mg, 0.20 mmol, 1.00 equiv.) and **2a** (75.7 mg, 0.30 mmol, 1.00 equiv.)**.** Purification on silica gel (Heptane/EtOAc = 3:1). Colorless oil (51.4 mg, 0.15 mmol, 72% yield).

^{1}H NMR (400 MHz, $CDCl_3$) 7.17 (d, ^3J = 8.2 Hz, 1H), 7.02 (dd, ^3J = 8.2 Hz, ^4J = 1.6 Hz, 1H), 6.91 (d, ^4J = 1.6 Hz, 1H), 3.63 (d, ^3J = 1.0 Hz, 2H), 2.97 – 2.78 (m, 3H), 2.38 – 2.31 (m, 1H), 2.21 (dd, ^2J = 12.4 Hz, ^3J = 2.0 Hz, 1H), 1.94 – 1.64 (m, 5H), 1.56 – 1.45 (m, 2H), 1.39 (s, 3H), 1.25 (s, 3H), 1.24 (d, ^3J = 6.9 Hz, 6H);

^{13}C{^{1}H} NMR (101 MHz, $CDCl_3$) δ 204.0, 146.3, 146.2, 134.6, 127.2, 124.2, 124.2, 116.3, 55.5, 46.1, 37.8, 37.7, 37.4, 33.6, 30.0, 25.6 (2C), 24.1, 21.7, 18.7, 16.7, 14.5;

HRMS (ESI) m/z: $[M + Na]^+$ Calcd for $C_{22}H_{29}NOSNa^+$ 378.1862; Found 378.1857;

IR (neat) ν 2930, 2249, 1688, 1496, 1458, 1384, 1201, 1146, 1040, 938, 921, 822, 779, 728, 651, 628 cm^{-1}.

2.6 Two Step Amides Synthesis - Optimization

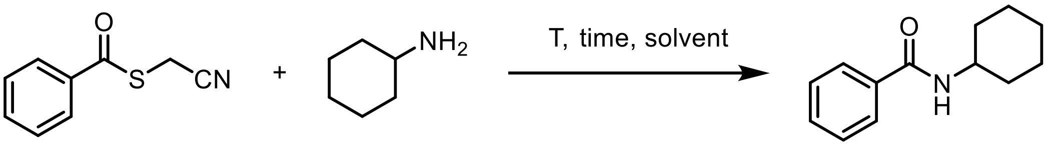

The thioester (0.2 mmol, 1.0 equiv.) was dissolved in the MeCN (2 ml, 0.1 M). Cyclohexylamine (46 µl, 0.4 mmol, 1.5 – 3.0 equiv.) was added and the reaction mixture was stirred for the stated time at the stated temperature. The reaction was quenched by addition of a saturated aqueous solution of NH_4Cl. MeCN was removed under reduced pressure. The aqueous layer was extracted with DCM (3x10 ml). The combined organic layers were dried over $MgSO_4$, filtered and the solvent was removed under reduced pressure.

entry	T	time [h]	amine [equiv.]	yield [%]
**1**	rt	2	2	76
**2**	rt	3.5	2	89
**3**	rt	20	2	>99 [92][a]
**4**	50 °C	2	2	89
**5**	50 °C	4	2	100
**6**	50 °C	4	3	100
**7**	50 °C	4	1.5	96

[a] isolated yield

2.7 General Procedure C: Amide Synthesis from Cyanomethyl Esters

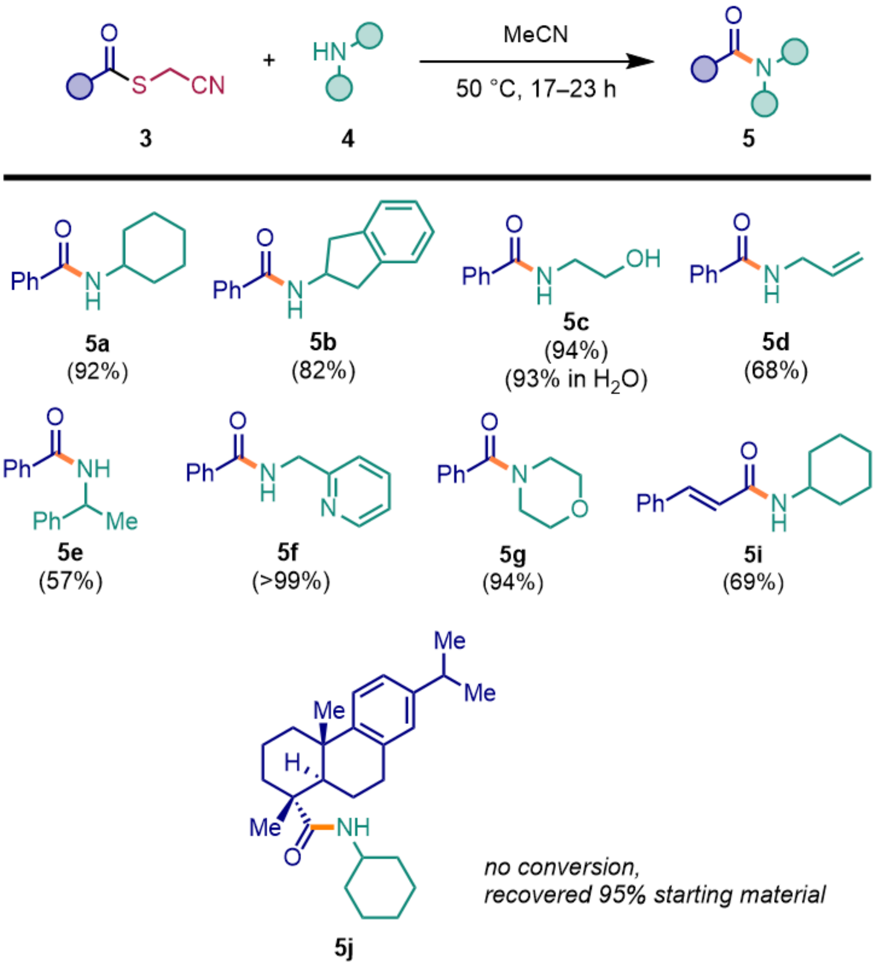

S-(Cyanomethyl) benzothioate (0.20 mmol, 1.00 equiv.) was dissolved in MeCN (2.0 ml, 0.1 M). The amine (0.40 mmol, 2.00 equiv.) was added and the reaction mixture was stirred for 17-23 h at 50 °C. The reaction was quenched by addition of a saturated aqueous solution of NH_4Cl (10 ml). MeCN was removed under reduced pressure. The aqueous layer was extracted with DCM (3x10 ml). The combined organic layers were dried over $MgSO_4$, filtered and the solvent was removed under reduced pressure. The product was purified by column chromatography.

2.8 Amide Characterization

N-cyclohexylbenzamide (5a)

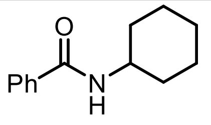

5a

Prepared according to general procedure **C** from *S*-(cyanomethyl) benzothioate (36.1 mg, 0.20 mmol, 1.00 equiv.) and cyclohexylamine (46 µl, 0.40 mmol, 2.00 equiv.). Purification on silica gel (DCM/MeOH = 100:0 to 100:1). White solid (37.4 mg, 0.18 mmol, 92% yield). Analytical data in accordance with the literature.[5]

^{1}H NMR (600 MHz, $CDCl_3$) δ 7.74 (dd, *J* = 5.2, 3.3 Hz, 2H), 7.48 – 7.43 (m, 1H), 7.42 – 7.37 (m, 2H), 6.10 (d, *J* = 5.2 Hz, $1H_{NH}$), 3.96 (tdt, *J* = 11.9, 8.0, 3.9 Hz, 1H), 2.06 – 1.96 (m, 2H), 1.78 – 1.70 (m, 2H), 1.67 – 1.60 (m, 1H), 1.46 – 1.35 (m, 2H), 1.29 – 1.13 (m, 3H);

^{13}C{^{1}H} NMR (151 MHz, $CDCl_3$) δ 166.7, 135.2, 131.3, 128.6 (2C), 127.0 (2C), 48.8, 33.3 (2C), 25.7, 25.0 (2C);

HRMS (ESI) m/z: $[M]^+$ Calcd for $C_{13}H_{18}NO^+$ 204.1388; Found 204.1381;

IR (neat) ν 3339, 3242, 2929, 2852, 2179, 2034, 1713, 1627, 1558, 742, 699 cm^{-1}.

N-(2,3-dihydro-1*H*-inden-2-yl)benzamide (5b)

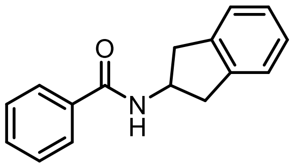

5b

Prepared according to general procedure **C** from *S*-(cyanomethyl) benzothioate (35.8 mg, 0.20 mmol, 1.00 equiv.) and 2-aminoindan (52.0 µl, 0.40 mmol, 2.00 equiv.). The reaction was run for 17 h. Purification on silica gel (DCM/MeOH = 100:0 to 100:1). Orange solid (37.4 mg, 0.16 mmol, 79% yield). Analytical data in accordance with the literature.[6]

^{1}H NMR (600 MHz, $CDCl_3$) δ 7.73 (d, *J* = 7.6 Hz, 2H), 7.47 (t, *J* = 7.4 Hz, 1H), 7.40 (t, *J* = 7.6 Hz, 2H), 7.28 – 7.23 (m, 2H), 7.22 – 7.18 (m, 2H), 6.37 (d, *J* = 5.0 Hz, $1H_{NH}$), 5.00 – 4.91 (m, 1H), 3.42 (dd, *J* = 16.2, 7.1 Hz, 2H), 2.93 (dd, *J* = 16.1, 4.4 Hz, 2H);

^{13}C{^{1}H} NMR (151 MHz, $CDCl_3$) δ 167.4, 141.0, 134.7, 131.6, 128.7 (2C), 127.0 (2C), 127.0 (2C), 125.0

(2C), 51.2, 40.4 (2C);

HRMS (ESI) m/z: $[M + H]^+$ Calcd for $C_{16}H_{16}NO^+$ 238.1231; Found 238.1228;

IR (neat) ν 3333, 3070, 3038, 3025, 2959, 2917, 2850, 1635, 1535, 736 cm^{-1}.

***N*-(2-hydroxyethyl)benzamide (5c)**

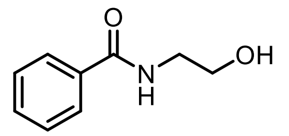

5c

Prepared according to general procedure **C** from *S*-(cyanomethyl) benzothioate (35.7 mg, 0.20 mmol, 1.00 equiv.) and 2-aminoethanol (24.1 µl, 0.40 mmol, 2.00 equiv.). The reaction was run for 23 h. Purification on silica gel (heptane/EtOAc = 9:1 to 0:10). Orange oil (31.4 mg, 0.19 mmol, 94% yield). Analytical data in accordance with the literature.[7]

^{1}H NMR (600 MHz, $CDCl_3$) δ 7.77 – 7.71 (m, 2H), 7.45 (t, *J* = 7.4 Hz, 1H), 7.36 (t, *J* = 7.7 Hz, 2H), 7.05 (s, $1H_{NH}$), 3.76 (t, *J* = 5.0 Hz, 2H), 3.56 (dd, *J* = 10.2, 5.4 Hz, 2H);

^{13}C{^{1}H} NMR (151 MHz, $CDCl_3$) δ 168.9, 134.2, 131.7, 128.7 (2C), 127.1 (2C), 62.0, 42.9;

HRMS (ESI) m/z: $[M + H]^+$ Calcd for $C_9H_{12}NO_2^+$ 166.0868; Found 166.0862;

IR (neat) ν 3319, 3063, 3031, 2927, 2876, 2851, 2361, 1714, 1640, 1539, 1292, 692 cm^{-1}.

***N*-allylbenzamide (5d)**

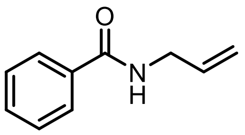

5d

Prepared according to general procedure **C** from *S*-(cyanomethyl) benzothioate (36.1 mg, 0.20 mmol, 1.00 equiv.) and allylamine (30 µl, 0.40 mmol, 2.00 equiv.). The reaction was run for 22 h. Purification on silica gel (DCM/MeOH = 100:0 to 100:1). Yellow oil (21.6 mg, 0.13 mmol, 67% yield). Analytical data in accordance with the literature.[8]

^{1}H NMR (600 MHz, $CDCl_3$) δ 7.81 – 7.75 (m, 2H), 7.52 – 7.47 (m, 1H), 7.46 – 7.40 (m, 2H), 6.22 (s, $1H_{NH}$), 5.94 (ddt, *J* = 17.1, 10.3, 5.7 Hz, 1H), 5.27 (ddd, *J* = 17.1, 3.0, 1.6 Hz, 1H), 5.19 (dq, *J* = 10.2, 1.3 Hz, 1H), 4.10 (tt, *J* = 5.7, 1.5 Hz, 2H);

$^{13}C\{^1H\}$ NMR (151 MHz, $CDCl_3$) δ 167.5, 134.6, 134.3, 131.7, 128.7 (2C), 127.0 (2C), 116.9, 42.6;

HRMS (ESI) m/z: $[M + H]^+$ Calcd for $C_{10}H_{12}NO^+$ 162.0919; Found 162.0911;

IR (neat) ν 3296, 3084, 3062, 3030, 2986, 2959, 2923, 2853, 1638, 1536, 1308, 693 cm^{-1}.

***N*-(1-phenylethyl)benzamide (5e)**

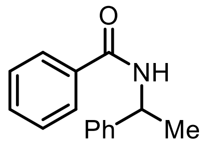

5e

Prepared according to general procedure **C** from *S*-(cyanomethyl) benzothioate (35.6 mg, 0.20 mmol, 1.00 equiv.) and (*S*)-(-)-phenylethylamine (51.6 µl, 0.40 mmol, 2.00 equiv.). The reaction was run for 17 h. Purification on silica gel (DCM/MeOH = 100:0 to 100:1). Yellow solid (25.7 mg, 0.11 mmol, 57% yield). Analytical data in accordance with the literature.[5]

^1H NMR (600 MHz, $CDCl_3$) δ 7.77 (dd, *J* = 5.1, 3.4 Hz, 2H), 7.51 – 7.47 (m, 1H), 7.44 – 7.38 (m, 4H), 7.38 – 7.34 (m, 2H), 7.30 – 7.26 (m, 1H), 6.38 (d, *J* = 6.1 Hz, $1H_{NH}$), 5.34 (p, *J* = 7.0 Hz, 1H), 1.61 (d, *J* = 6.9 Hz, 3H);

$^{13}C\{^1H\}$ NMR (151 MHz, $CDCl_3$) δ 166.7, 143.3, 134.7, 131.6, 128.9 (2C), 128.7 (2C), 127.6, 127.1 (2C), 126.4 (2C), 49.3, 21.9;

HRMS (ESI) m/z: $[M + H]^+$ Calcd for $C_{15}H_{16}NO^+$ 226.1231; Found 226.1228;

IR (neat) ν 3326, 3060, 3030, 2974, 2919, 2851, 1634, 1525, 697 cm^{-1}.

***N*-(pyridin-2-ylmethyl)benzamide (5f)**

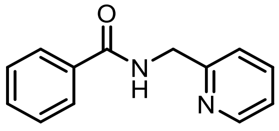

5f

Prepared according to general procedure **C** from *S*-(cyanomethyl) benzothioate (35.3 mg, 0.20 mmol, 1.00 equiv.) and 2-(aminomethyl)pyridine (41.2 µl, 0.40 mmol, 2.00 equiv.). The reaction was run for 17 h. Purification on silica gel (DCM/MeOH = 100:0 to 70:1). Yellow oil (42.7 mg, 0.20 mmol, >99% yield). Analytical data in accordance with the literature.[5]

^1H NMR (600 MHz, $CDCl_3$) δ 8.54 (d, *J* = 4.5 Hz, 1H), 7.89 – 7.84 (m, 2H), 7.68 (s, $1H_{NH}$), 7.66 (td, *J* =

7.7, 1.7 Hz, 1H), 7.48 (ddd, *J* = 6.7, 3.9, 1.2 Hz, 1H), 7.42 (dd, *J* = 10.4, 4.6 Hz, 2H), 7.31 (d, *J* = 7.8 Hz, 1H), 7.20 (dd, *J* = 7.0, 5.3 Hz, 1H), 4.74 (d, *J* = 4.9 Hz, 2H);

$^{13}C\{^1H\}$ NMR (151 MHz, $CDCl_3$) δ 167.5, 156.4, 149.1, 136.9, 134.5, 131.6, 128.6 (2 C), 127.2 (2 C), 122.5, 122.3, 44.9;

HRMS (ESI) m/z: $[M + H]^+$ Calcd for $C_{13}H_{13}N_2O^+$ 213.1028; Found 213.1016;

IR (neat) ν 3371, 3323, 3275, 3085, 3064, 3008, 2930, 2853, 2360, 1641, 1538, 1310, 711 cm^{-1}.

morpholino(phenyl)methanone (5g)

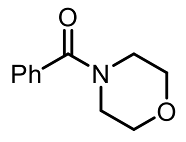

5g

Prepared according to general procedure **C** from *S*-(cyanomethyl) benzothioate (35.8 mg, 0.20 mmol, 1.00 equiv.) and morpholine (35.0 µl, 0.40 mmol, 2.00 equiv.). The reaction was run for 23 h. Purification on silica gel (DCM:MeOH:DMA = 100:0:1 to 100:2:1). Dark orange oil (34.8 mg, 0.18 mmol, 91% yield). Analytical data in accordance with the literature.[9]

^1H NMR (600 MHz, $CDCl_3$) δ 7.42 – 7.39 (m, 5H), 3.77 – 3.43 (m, 8H);

$^{13}C\{^1H\}$ NMR (151 MHz, $CDCl_3$) δ 170.6, 135.4, 130.0, 128.7, 127.2, 67.0, 48.3, 42.6;

HRMS (ESI) m/z: $[M + H]^+$ Calcd for $C_{11}H_{14}NO_2^+$ 192.1025; Found 192.1019;

IR (neat) ν 3059, 2964, 2922, 2854, 2360, 1631, 1427, 1278, 1016, 710 cm^{-1}.

N-cyclohexylcinnamamide (5i)

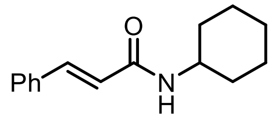

5i

Prepared according to general procedure **C** from **3i** (40.6 mg, 0.20 mmol, 1.00 equiv.) and cyclohexylamine (46 µl, 0.40 mmol, 2.00 equiv.). The reaction was run for 23 h. Purification on silica gel (Heptane:Ethyl acetate = 2:1). White solid (31.6 mg, 0.14 mmol, 69% yield). Analytical data in accordance with the literature.[11]

^{1}H NMR (400 MHz, $CDCl_3$) δ 7.61 (d, *J* = 15.6 Hz, 1H), 7.49 (dd, *J* = 7.5, 1.8 Hz, 2H), 7.41 – 7.32 (m, 3H), 6.36 (d, *J* = 15.6 Hz, 1H), 5.47 (d, *J* = 6.5 Hz, 1H), 3.92 (tdt, *J* = 12.0, 8.0, 3.9 Hz, 1H), 2.06 – 1.90 (m, 2H), 1.79 – 1.70 (m, 2H), 1.69 – 1.60 (m, 1H), 1.41 (tt, *J* = 15.5, 3.4 Hz, 2H), 1.27 – 1.08 (m, 4H).

2.9 One-Pot Procedure – Optimization

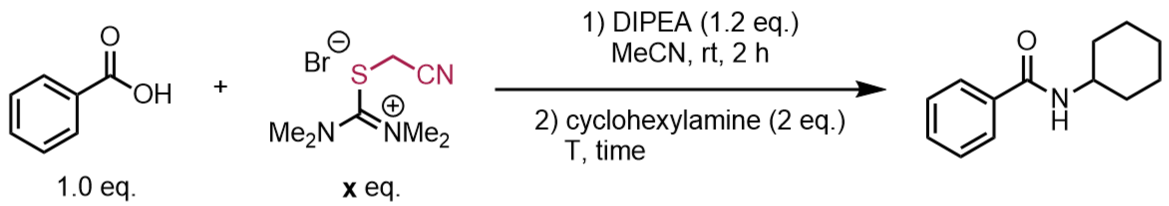

2-(Cyanomethyl)-1,1,3,3-tetramethylisothiouronium bromide (1.00–1.50 equiv.) and benzoic acid (0.20 mmol, 1.00 equiv.) were dissolved in MeCN (2.0 ml, 0.1 M). The reaction mixture was stirred at rt for 2 h. Then, cyclohexylamine (2.0 equiv.) was added and stirring was continued for the stated time at the stated temperature. The reaction was quenched by addition of a saturated aqueous solution of NH_4Cl (10 ml). MeCN was removed under reduced pressure and the aqueous layer was extracted with DCM (3x10–20 ml). The combined organic layers were dried over $MgSO_4$, filtered and the solvent was removed under reduced pressure. The NMR yield was determined using mesitylene as the internal standard.

entry	isothiouronium salt [equiv.]	T [°C]	time [h]	yield [%]
**1**	1	50	18	88
**2**	1.5	50	18	94 [50]
**3**	1.5	50	4	73
**4**	1.5	rt	18	82

2.10 General Procedure D: One-Pot Amide Synthesis

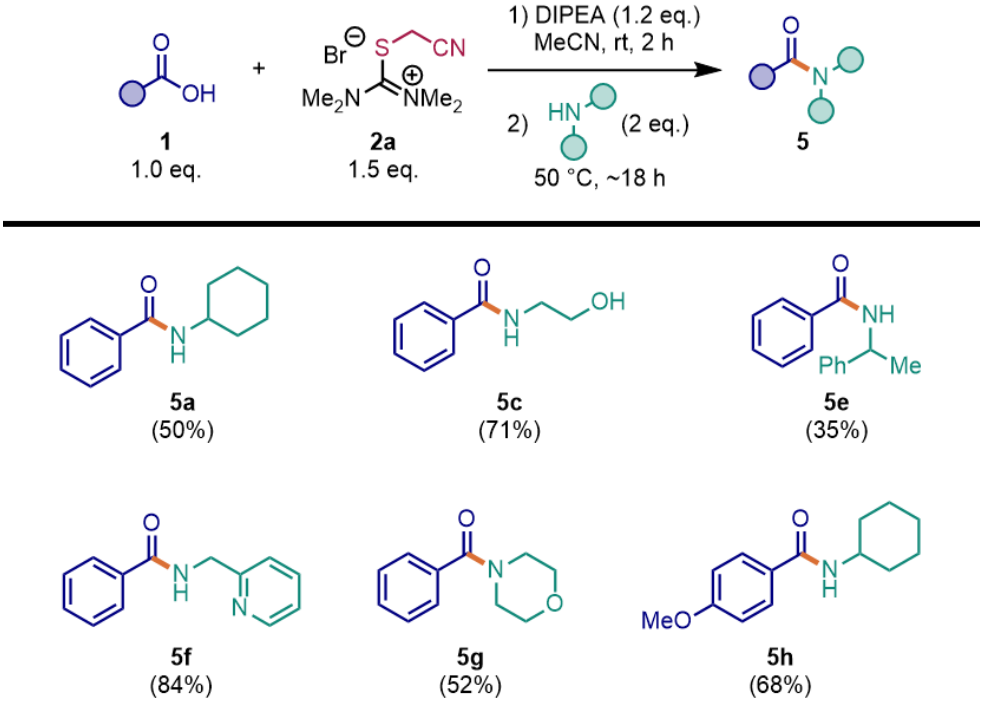

2-(Cyanomethyl)-1,1,3,3-tetramethylisothiouronium bromide (0.30 mmol, 1.50 equiv.) and the carboxylic acid (0.20 mmol, 1.00 equiv.) were dissolved in MeCN (2.0 ml, 0.1 M). The reaction mixture was stirred at rt for 2 h. Then the amine (0.40 mmol, 2.00 equiv.) was added and stirring was continued for the stated time at 50 °C. The reaction was quenched by addition of a saturated aqueous solution of NH_4Cl (10 ml). MeCN was removed under reduced pressure and the aqueous layer was extracted with DCM (3x10–20 ml). The combined organic layers were dried over $MgSO_4$, filtered and the solvent was removed under reduced pressure. The crude material was purified by column chromatography using a gradient of DCM/MeOH or heptane/EtOAc or DCM/NEt_3 or DCM/heptane.

***N*-cyclohexylbenzamide (5a)**

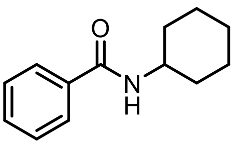

5a

Prepared according to general procedure **D** from **2a** (75.7 mg, 0.30 mmol, 1.50 equiv.) benzoic acid (24.8 mg, 0.20 mmol, 1.00 equiv.) and cyclohexylamine (42.0 µl, 0.40 mmol, 2.00 equiv.). The reaction was run for 18 h. Purification on silica gel (DCM/MeOH = 100:0 to 200:1). Orange solid (21.2 mg, 0.10 mmol, 51%). Analytical data in accordance with the literature.[5]

***N*-(2-hydroxyethyl)benzamide (5c)**

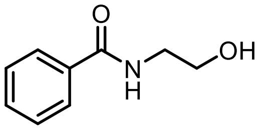

5c

Prepared according to general procedure **D** from **2a** (75.7 mg, 0.30 mmol, 1.50 equiv.), benzoic acid (24.2 mg, 0.20 mmol, 1.00 equiv.) and 2-aminoethanol (60.4 µl, 1.0 mmol, 5.0 equiv.). The reaction was run for 22 h. Purification on silica gel (DCM/MeOH = 100:0 to 40:1). Orange solid (23.2 mg, 0.14 mmol, 71% yield). Analytical data in accordance with the literature.[7]

(S)-*N*-(1-phenylethyl)benzamide (5e)

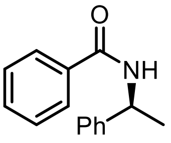

5e

Prepared according to general procedure **D** from **2a** (75.7 mg, 0.30 mmol, 1.50 equiv.) benzoic acid (24.8 mg, 0.20 mmol, 1.0 equiv.) and (*S*)-(-)-1-phenylethylamine (51.6 µl, 0.40 mmol, 1.20 equiv.). The reaction was run for 19 h. Purification on silica gel (heptane/EtOAc = 15:1 to 7:1). White solid (15.9 mg, 0.07 mmol, 35% yield). Analytical data in accordance with the literature.[5]

***N*-(pyridin-2-ylmethyl)benzamide (5f)**

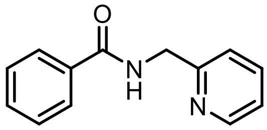

5f

Prepared according to general procedure **D** from **2a** (75.7 mg, 0.31 mmol, 1.50 equiv.), benzoic acid (24.6 mg, 0.20 mmol, 1.00 equiv.) and 2-(aminomethyl)pyridine (41.2 µl, 0.40 mmol, 2.00 equiv.). The reaction was run for 22 h. Purification on silica gel (DCM/NEt_3 = 100:1). Yellow oil (36.0 mg, 0.17 mmol, 84% yield). Analytical data in accordance with the literature.[5]

morpholino(phenyl)methanone (5g)

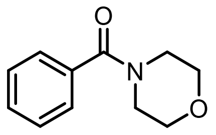

5g

Prepared according to general procedure **D** from **2a** (75.7 mg, 0.30 mmol, 1.50 equiv.), benzoic acid (24.7 mg, 0.20 mmol, 1.00 equiv.) and morpholine (35.0 µl, 0.40 mmol, 2.00 equiv.). The reaction was run for 22 h. Purification on silica gel (DCM/MeOH = 100:0 to 200:1). Orange solid (19.9 mg, 0.10 mmol, 52%). Analytical data in accordance with the literature.[9]

***N*-cyclohexyl-4-methoxybenzamide (5h)**

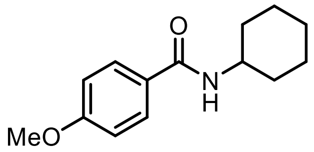

5h

Prepared according to general procedure **D** from **2a** (75.7 mg, 0.30 mmol, 1.50 equiv.), p-methoxy benzoic acid (30.9 mg, 0.20 mmol, 1.00 equiv.) and cyclohexylamine (45.8 µl, 0.40 mmol, 2.00 equiv.). The reaction was run for 22 hPurification of on silica gel (heptane/EtOAc = 7:1 to 4:1). White solid (32.0 mg, 0.14 mmol, 68% yield). Analytical data in accordance with the literature.[10]

^{1}H NMR (700 MHz, CDCl$_3$) δ 7.77 – 7.67 (m, 2H), 6.93 – 6.83 (m, 2H), 5.97 (d, *J* = 6.8 Hz, 1H), 3.99 – 3.89 (m, 1H), 3.82 (s, 3H), 2.04 – 1.96 (m, 2H), 1.77 – 1.71 (m, 2H), 1.66 – 1.59 (m, 1H), 1.44 – 1.35 (m, 2H), 1.27 – 1.13 (m, 3H).

^{13}C{^{1}H} NMR (176 MHz, CDCl$_3$) δ 166.2, 162.1, 128.7 (2 C), 127.5, 113.7 (2 C), 55.5, 48.7, 33.4, 25.7, 25.1.

IR (neat) ν 3300, 3005, 2933, 2917, 2852, 2360, 1736, 1713, 1625, 1254, 740 cm^{-1}.

HRMS (ESI) m/z: [M + H]$^+$ Calcd for $C_{14}H_{20}NO_2^+$ 234.1489; Found 234.1490.

2.11 General Procedure E: Amide Synthesis in Water

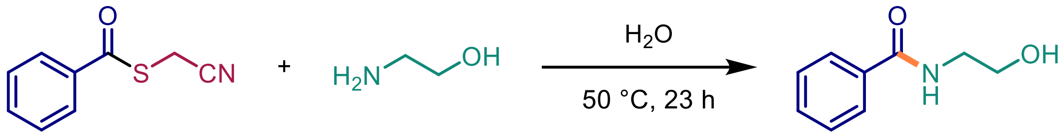

3a **5c**

S-(Cyanomethyl) benzothioate (0.20 mmol, 1.00 equiv.) was dissolved in distilled water (2.0 ml, 0.1 M). The amine (0.40 mmol, 2.00 equiv.) was added and the reaction mixture was stirred for 23 h at 50 °C. The reaction was quenched by addition of a saturated aqueous solution of NH_4Cl (10 ml). The solution was extracted with DCM (3x10 ml). The combined organic layers were dried over $MgSO_4$, filtered and the solvent was removed under reduced pressure. The product was purified by column chromatography.

N-(2-hydroxyethyl)benzamide (5c)

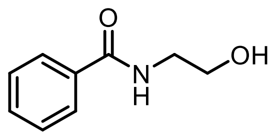

5c

Prepared according to general procedure **E** from *S*-(cyanomethyl) benzothioate (35.7 mg, 0.20 mmol, 1.00 equiv.) and 2-aminoethanol (24.1 µl, 0.40 mmol, 2.00 equiv.). The reaction was run for 23 h. Purification on silica gel (heptane/EtOAc = 9:1 to 0:10). Orange oil (30.6 mg, 0.19 mmol, 93% yield). Analytical data in accordance with the literature.[7]

3. NMR Spectra

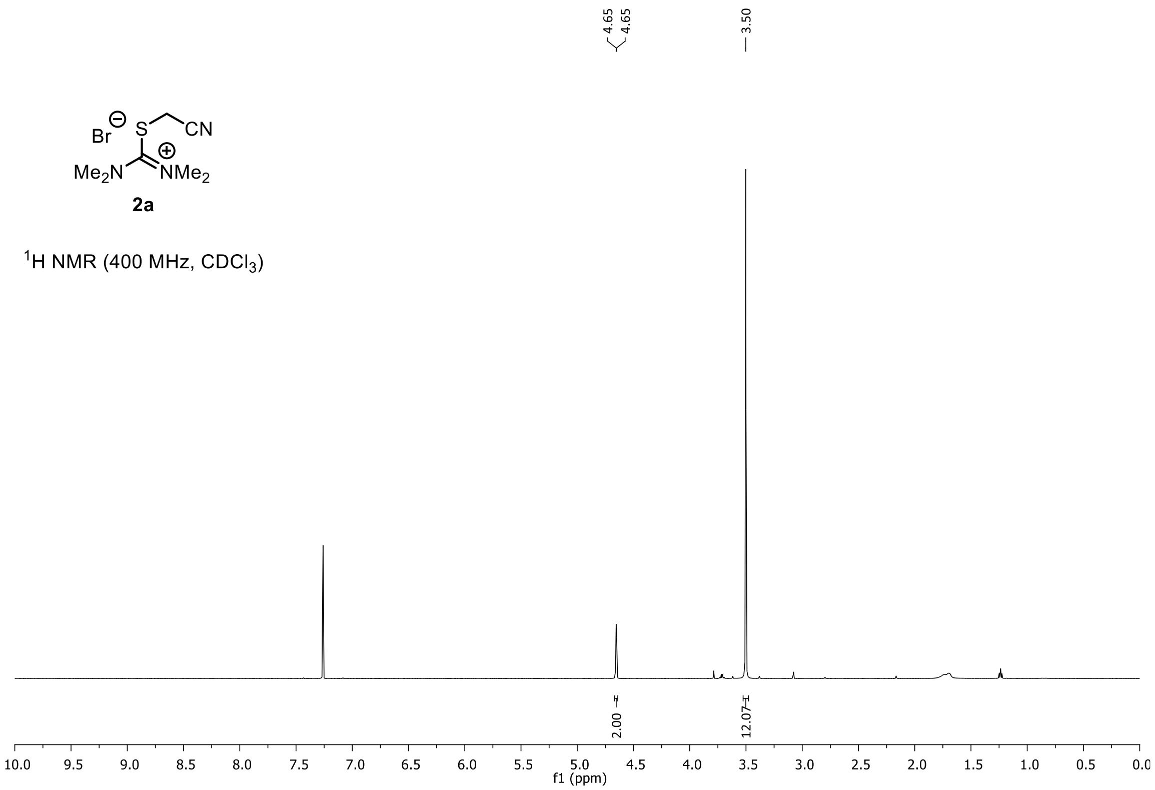

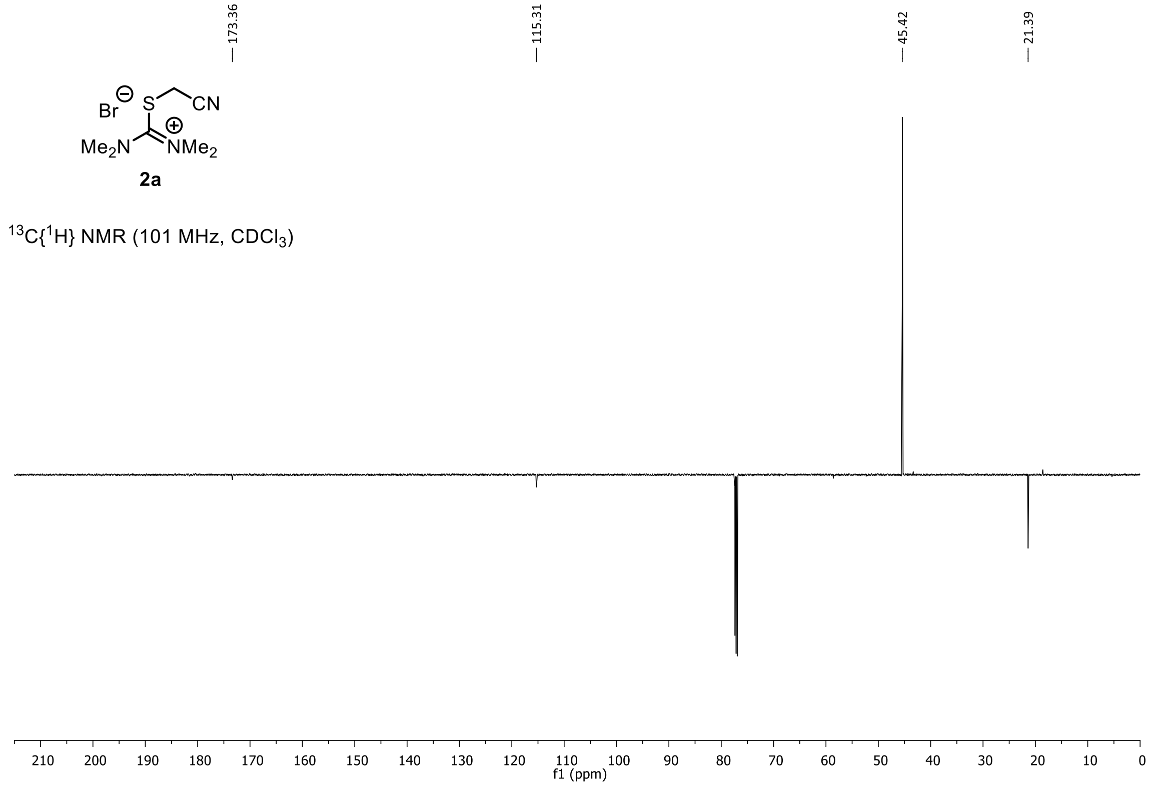

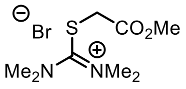

2b

^{1}H NMR (400 MHz, $CDCl_3$)

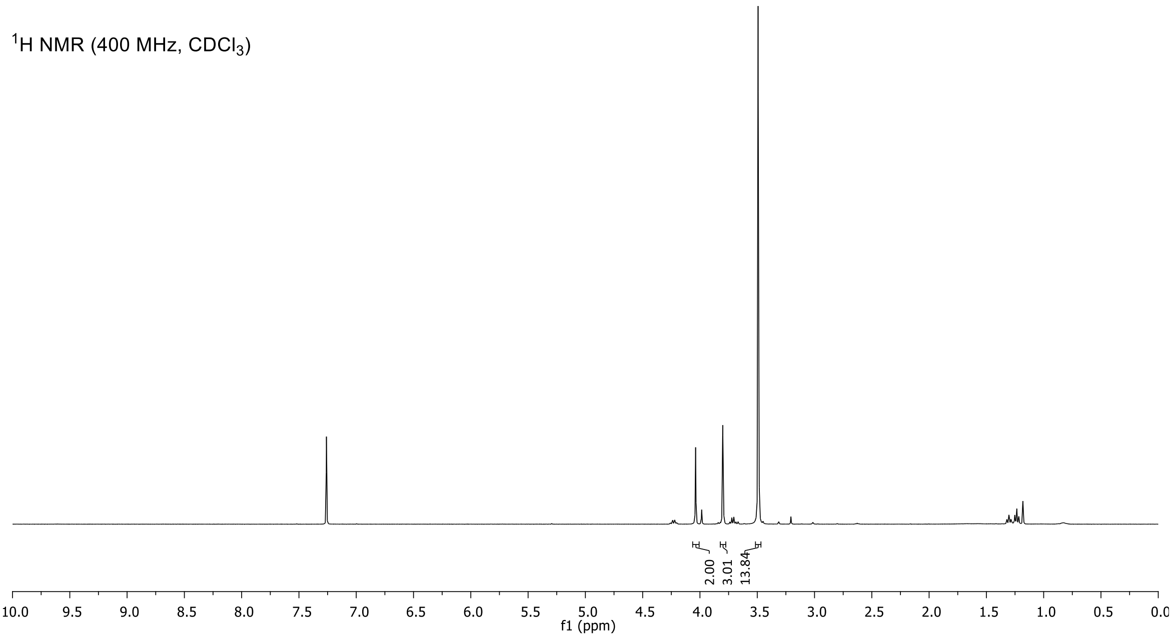

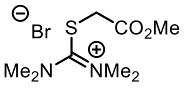

2b

$^{13}C\{^{1}H\}$ NMR (101 MHz, $CDCl_3$)

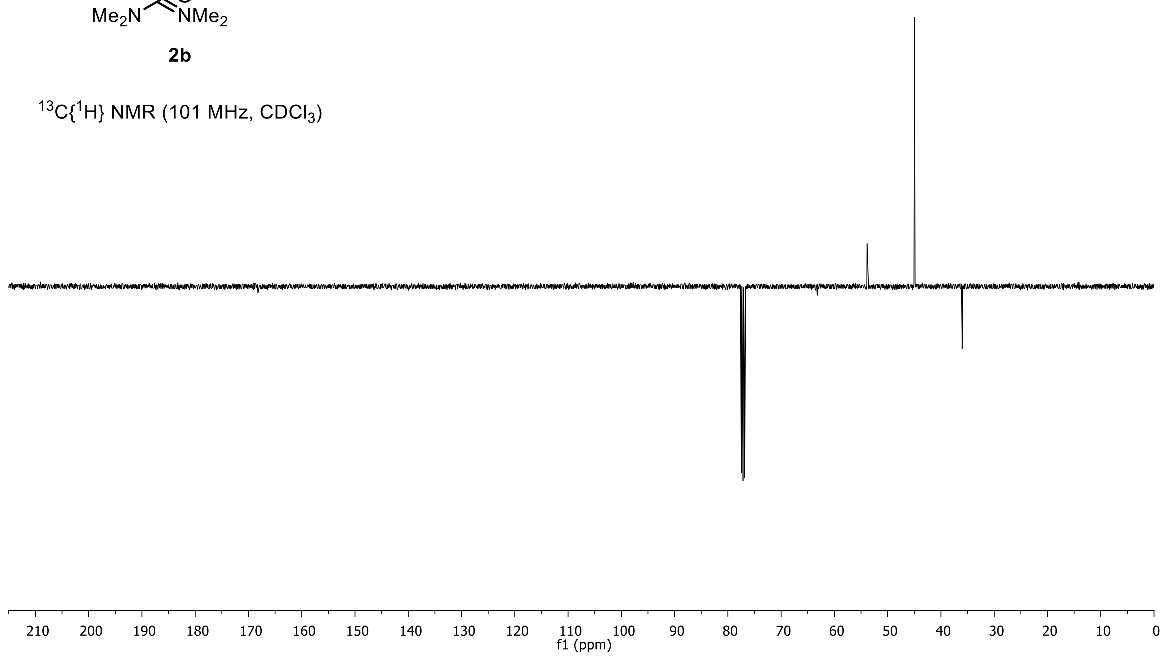

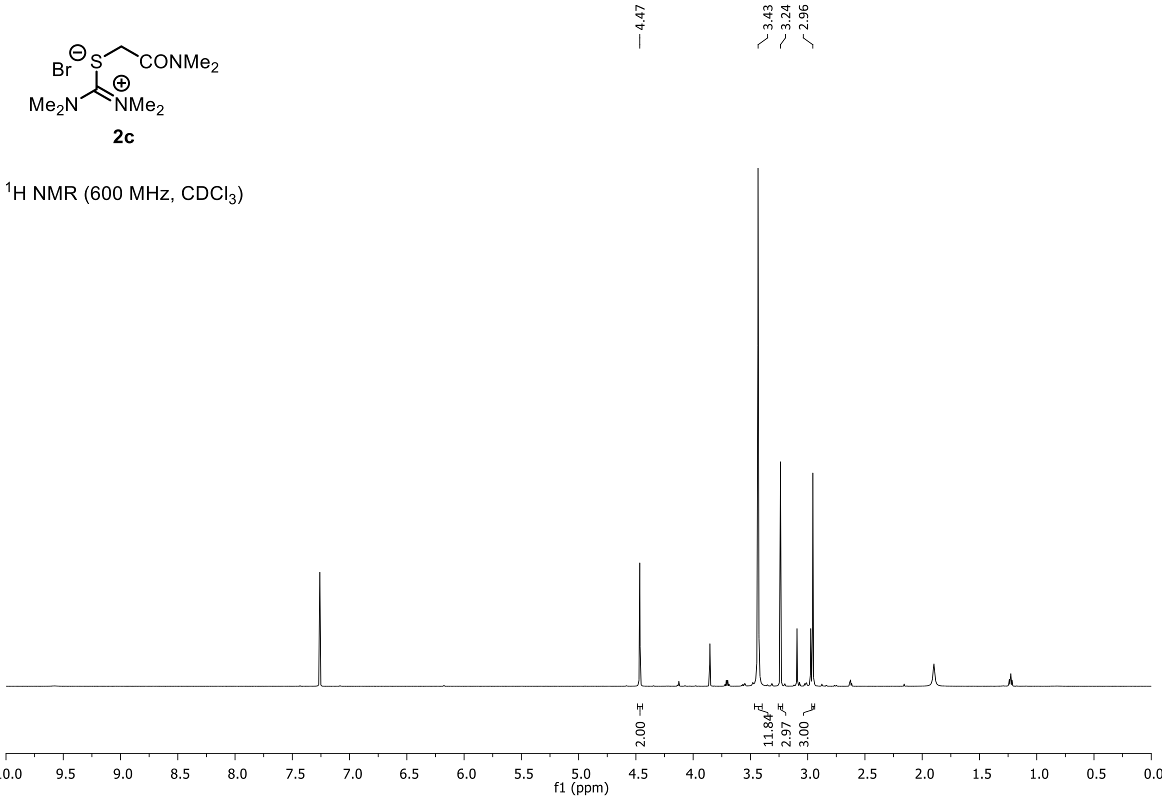
4.47
3.43
3.24
2.96
Br
S
CONNMe2
Me2N
NMe2
2c
1H NMR (600 MHz, CDCl3)
2.00
11.84
2.97
3.00
f1 (ppm)

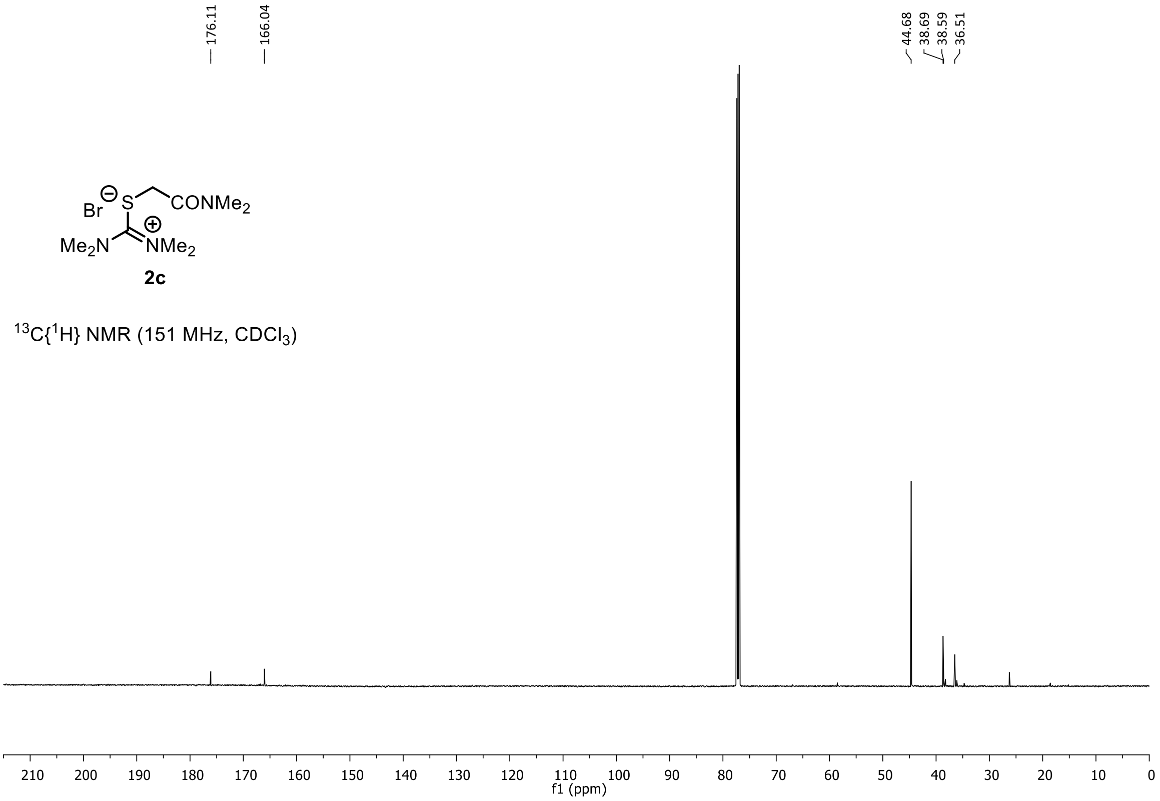
176.11
166.04
44.68
38.69
38.59
36.51
Br
S
CONMe2
Me2N
NMe2
2c
13C{1H} NMR (151 MHz, CDCl3)
f1 (ppm)

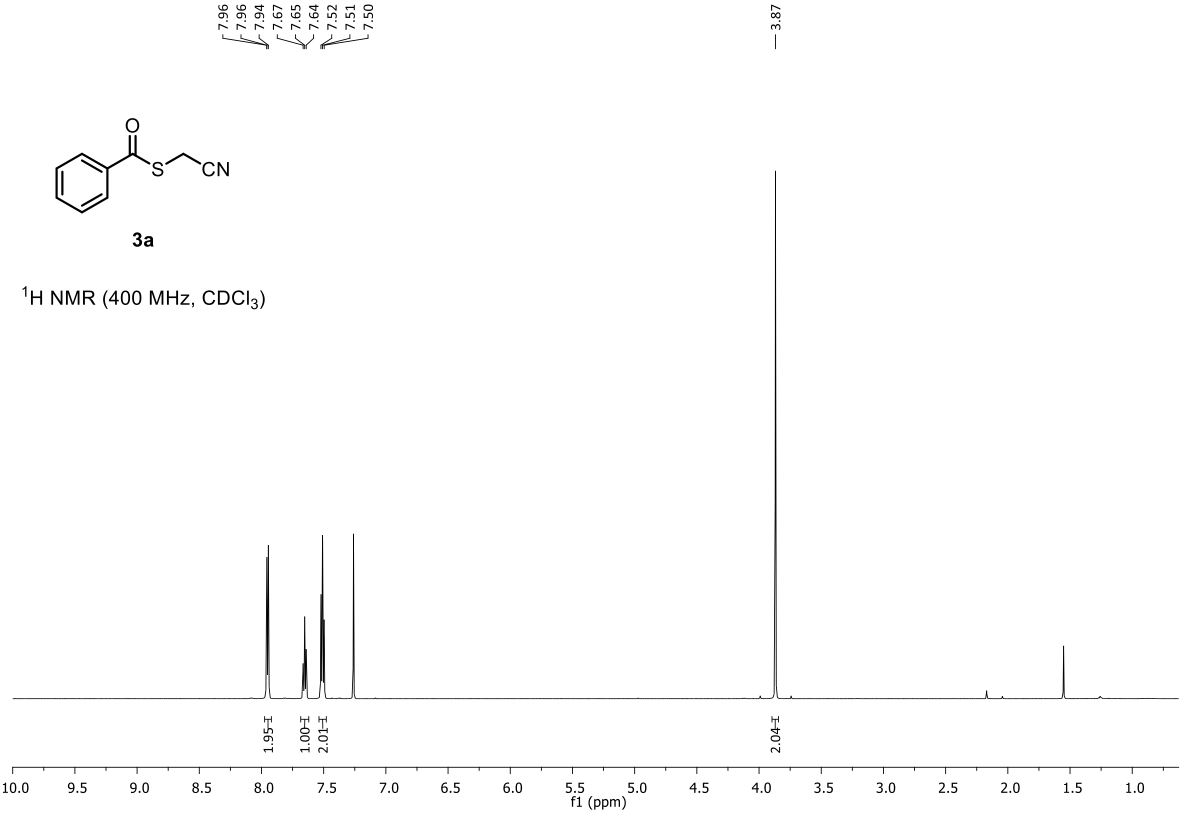
7.96
7.96
7.94
7.67
7.65
7.64
7.52
7.51
7.50
3.87
O
S
CN
3a
^{1}H NMR (400 MHz, CDCl$_3$)
1.95
1.00
2.01
2.04
10.0
9.5
9.0
8.5
8.0
7.5
7.0
6.5
6.0
5.5
5.0
4.5
4.0
3.5
3.0
2.5
2.0
1.5
1.0
f1 (ppm)

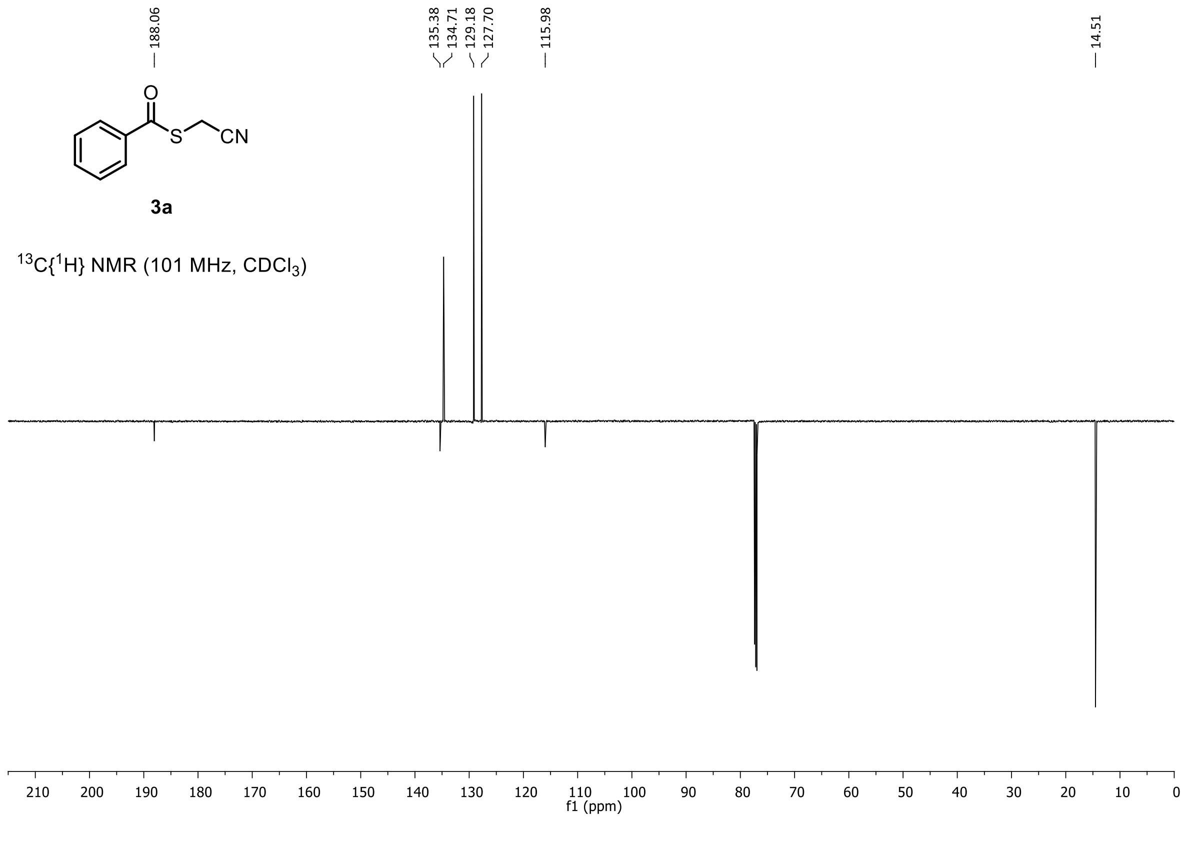
188.06
135.38
134.71
129.18
127.70
115.98
14.51
O
S
CN
3a
^{13}C{^{1}H} NMR (101 MHz, CDCl$_3$)
210
200
190
180
170
160
150
140
130
120
110
100
90
80
70
60
50
40
30
20
10
0
f1 (ppm)

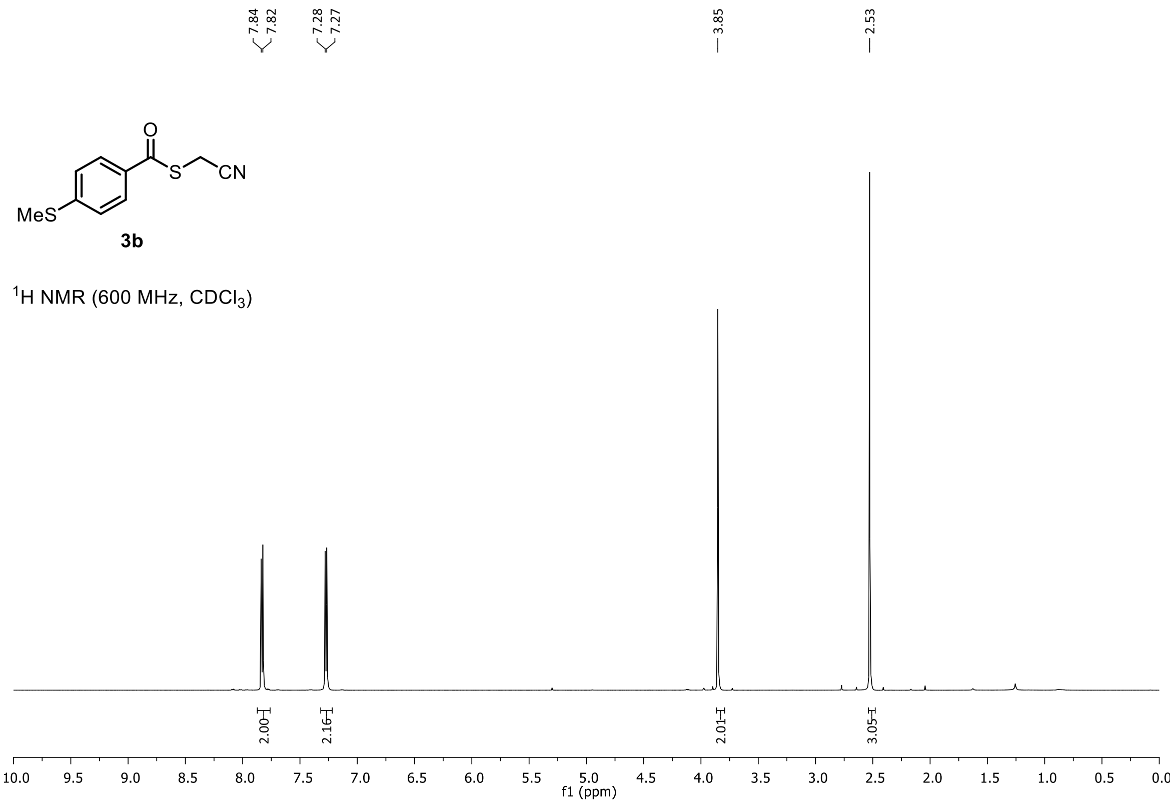
7.84
7.82
7.28
7.27
3.85
2.53
MeS
S
CN
O
3b
^{1}H NMR (600 MHz, CDCl$_3$)
2.00
2.16
2.01
3.05
10.0 9.5 9.0 8.5 8.0 7.5 7.0 6.5 6.0 5.5 5.0 4.5 4.0 3.5 3.0 2.5 2.0 1.5 1.0 0.5 0.0
f1 (ppm)

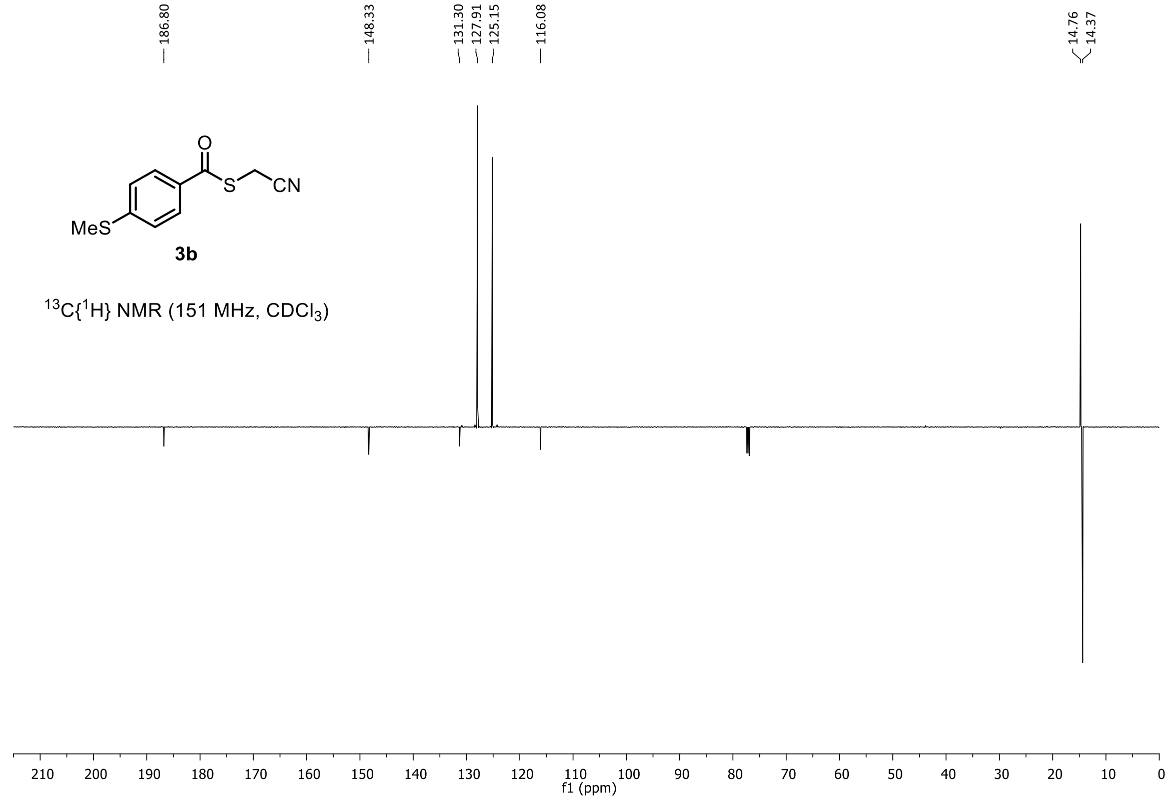
186.80
148.33
131.30
127.91
125.15
116.08
14.76
14.37
MeS
S
CN
O
3b
^{13}C{^{1}H} NMR (151 MHz, CDCl$_3$)
210 200 190 180 170 160 150 140 130 120 110 100 90 80 70 60 50 40 30 20 10 0
f1 (ppm)

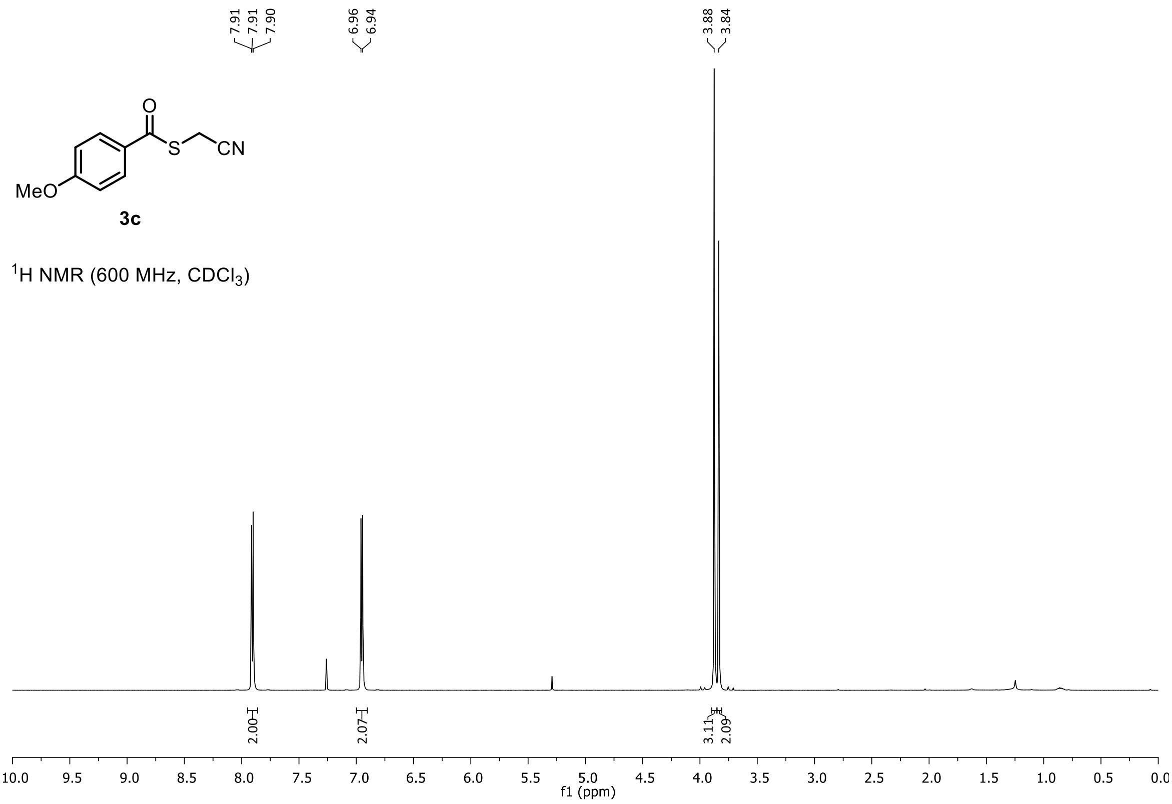
7.91
7.91
7.90
6.96
6.94
3.88
3.84
MeO
S
CN
O
3c
1H NMR (600 MHz, CDCl3)
2.00
2.07
3.11
2.09
10.0 9.5 9.0 8.5 8.0 7.5 7.0 6.5 6.0 5.5 5.0 4.5 4.0 3.5 3.0 2.5 2.0 1.5 1.0 0.5 0.0
f1 (ppm)

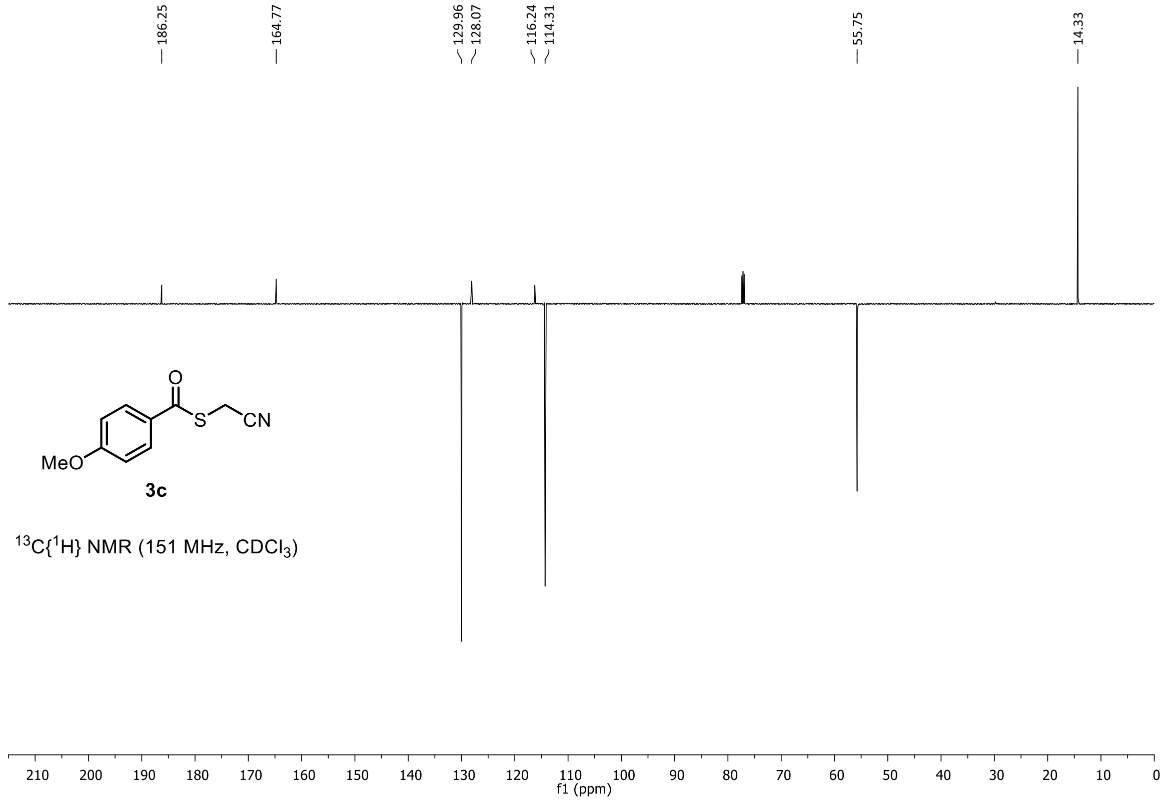
186.25
164.77
129.96
128.07
116.24
114.31
55.75
14.33
MeO
S
CN
O
3c
13C{1H} NMR (151 MHz, CDCl3)
210 200 190 180 170 160 150 140 130 120 110 100 90 80 70 60 50 40 30 20 10 0
f1 (ppm)

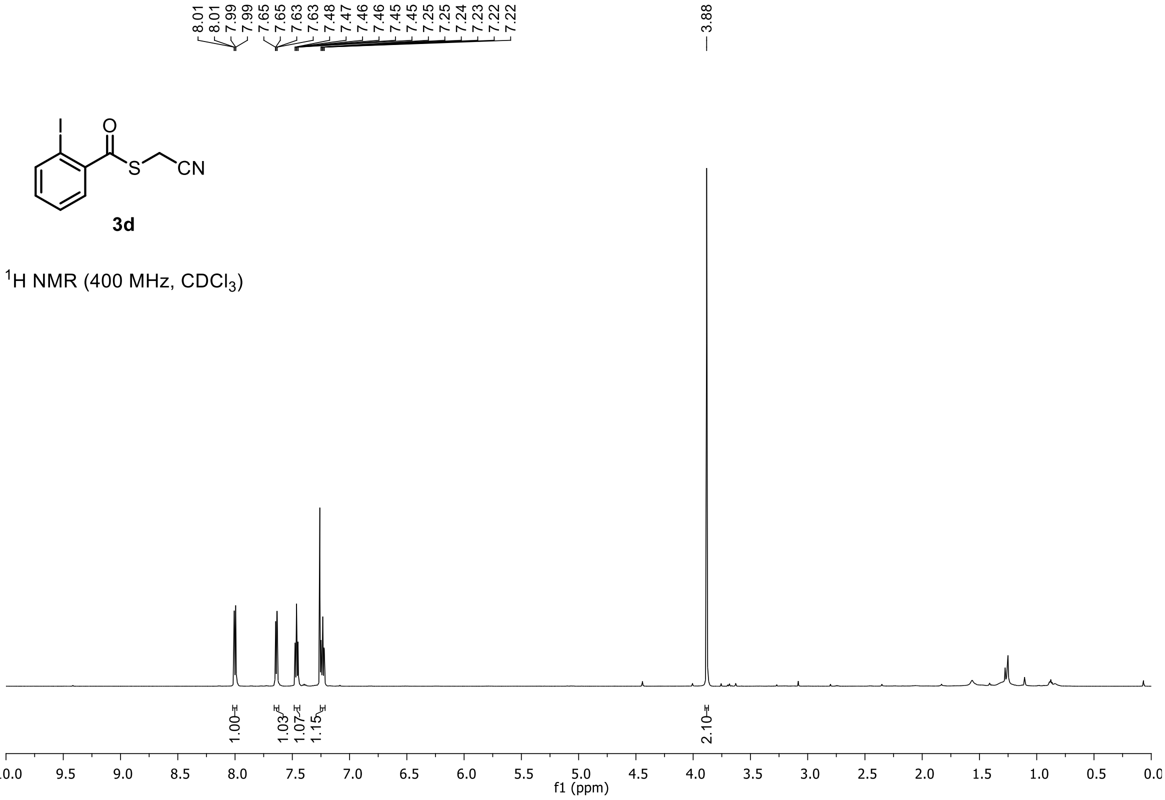
8.01
8.01
7.99
7.65
7.65
7.63
7.63
7.48
7.47
7.46
7.45
7.45
7.25
7.24
7.23
7.22
7.22
3.88
3d
1H NMR (400 MHz, CDCl3)
1.00
1.03
1.07
1.15
2.10
10.0 9.5 9.0 8.5 8.0 7.5 7.0 6.5 6.0 5.5 5.0 4.5 4.0 3.5 3.0 2.5 2.0 1.5 1.0 0.5 0.0
f1 (ppm)

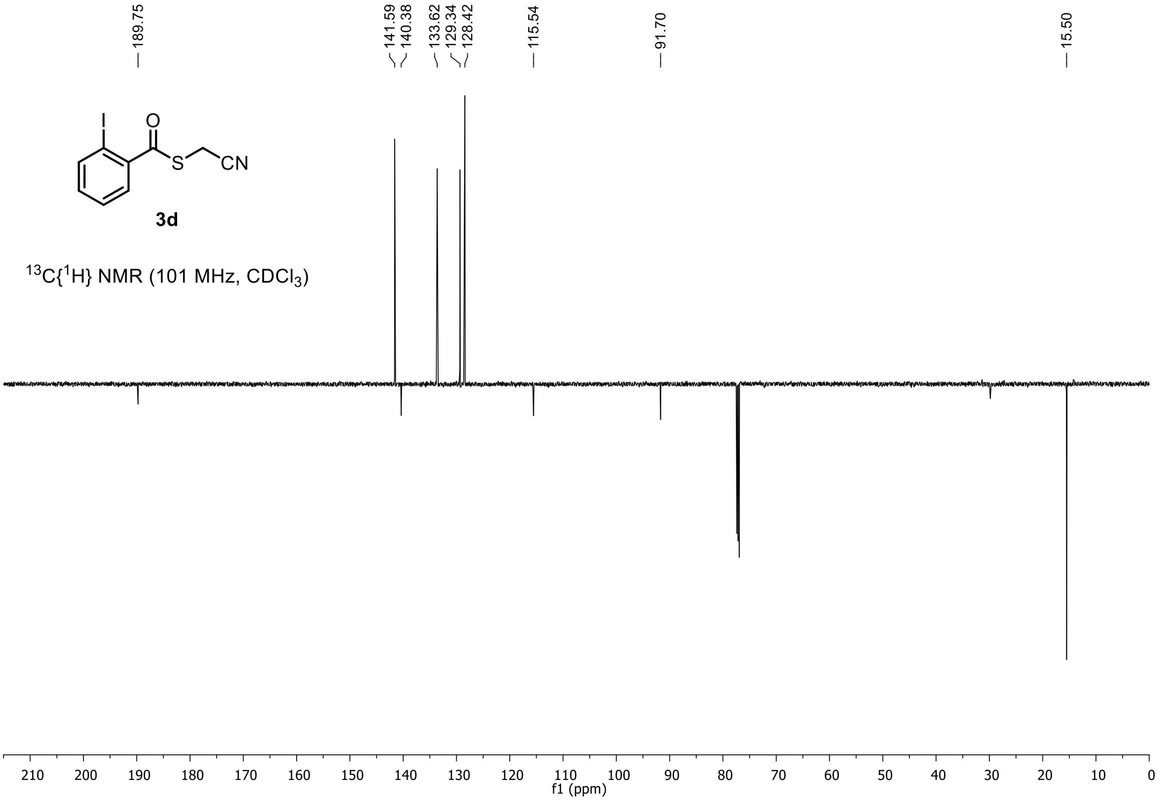
189.75
141.59
140.38
133.62
129.34
128.42
115.54
91.70
15.50
3d
13C{1H} NMR (101 MHz, CDCl3)
210 200 190 180 170 160 150 140 130 120 110 100 90 80 70 60 50 40 30 20 10 0
f1 (ppm)

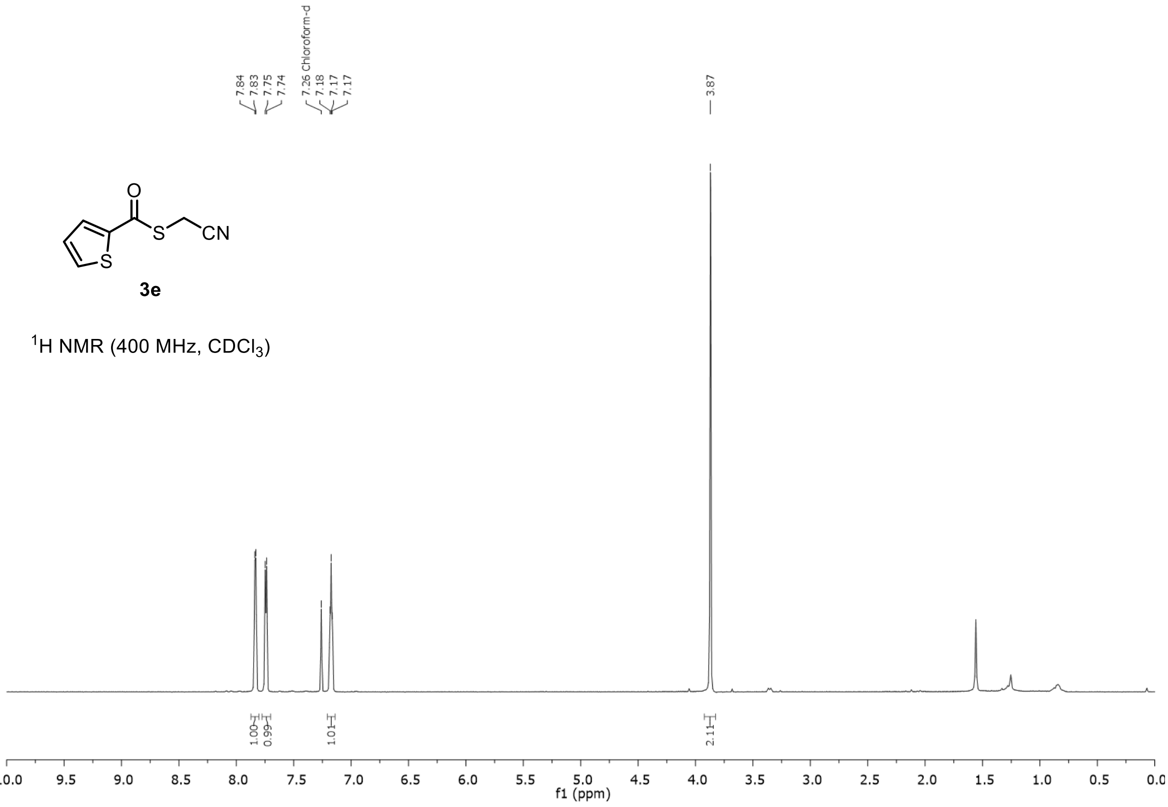
7.84
7.83
7.75
7.74
7.26 Chloroform-d
7.18
7.17
7.17
3.87
O
S
CN
S
3e
1H NMR (400 MHz, CDCl3)
1.00
0.99
1.01
2.11
f1 (ppm)

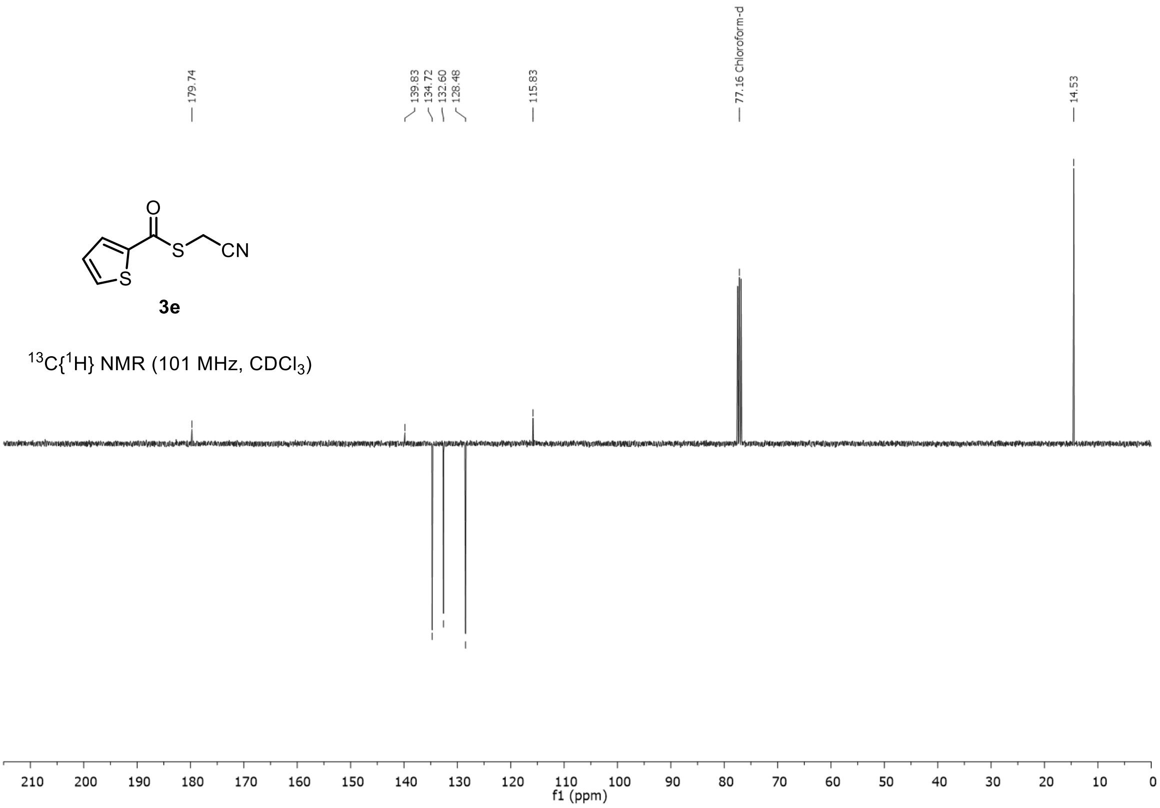
179.74
139.83
134.72
132.60
128.48
115.83
77.16 Chloroform-d
14.53
O
S
CN
S
3e
13C{1H} NMR (101 MHz, CDCl3)
f1 (ppm)

3f

^{1}H NMR (400 MHz, $CDCl_3$)

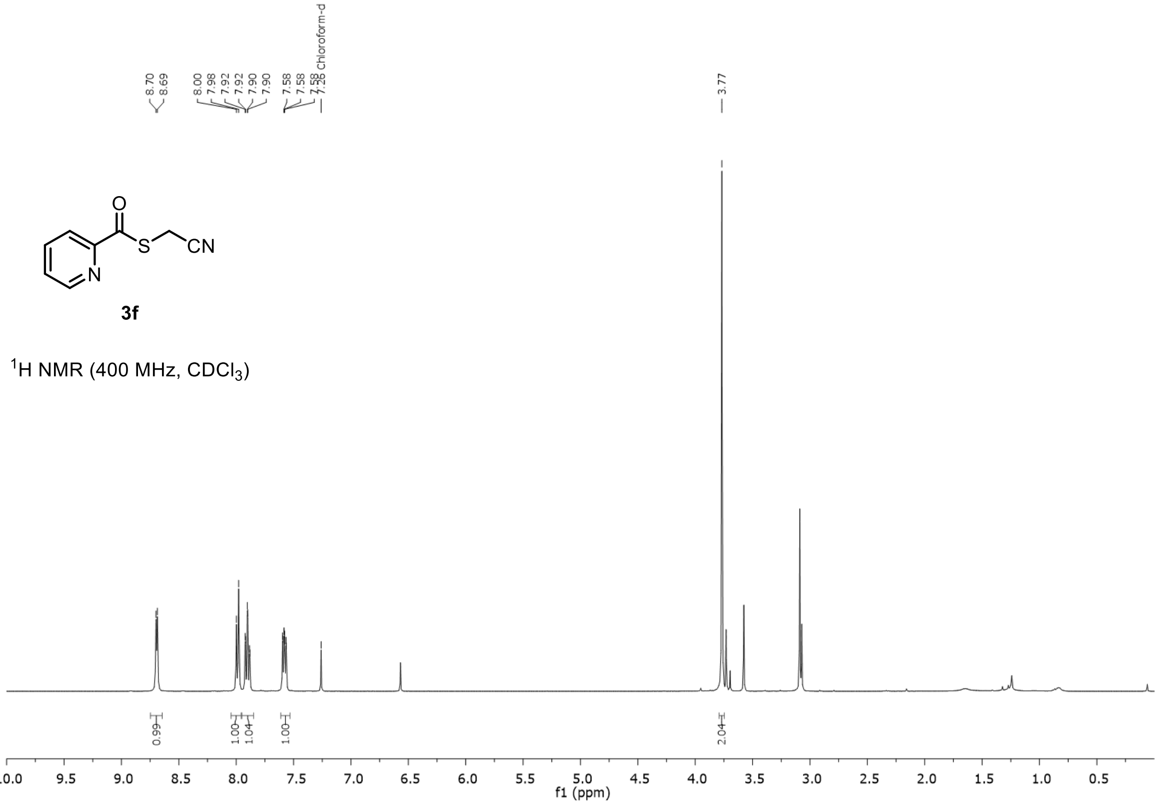

3f

$^{13}C\{^{1}H\}$ NMR (101 MHz, $CDCl_3$)

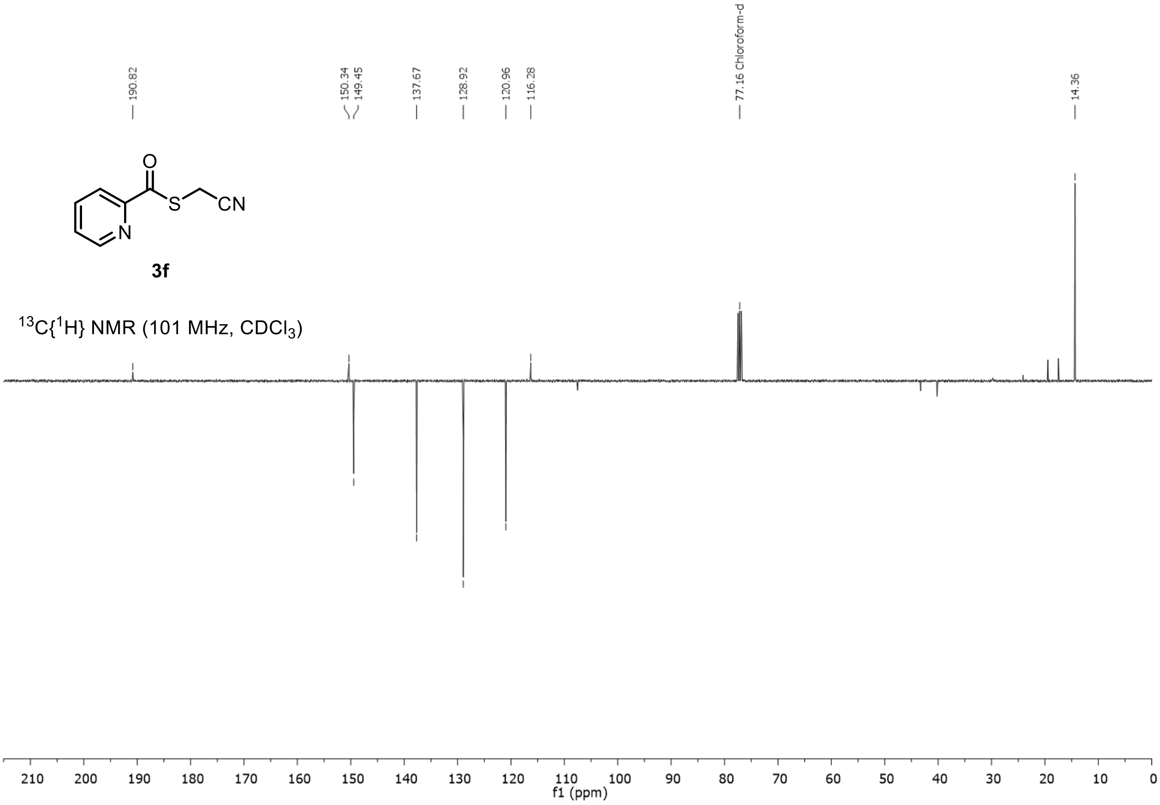

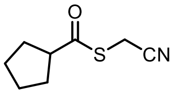

3g

^{1}H NMR (400 MHz, $CDCl_3$)

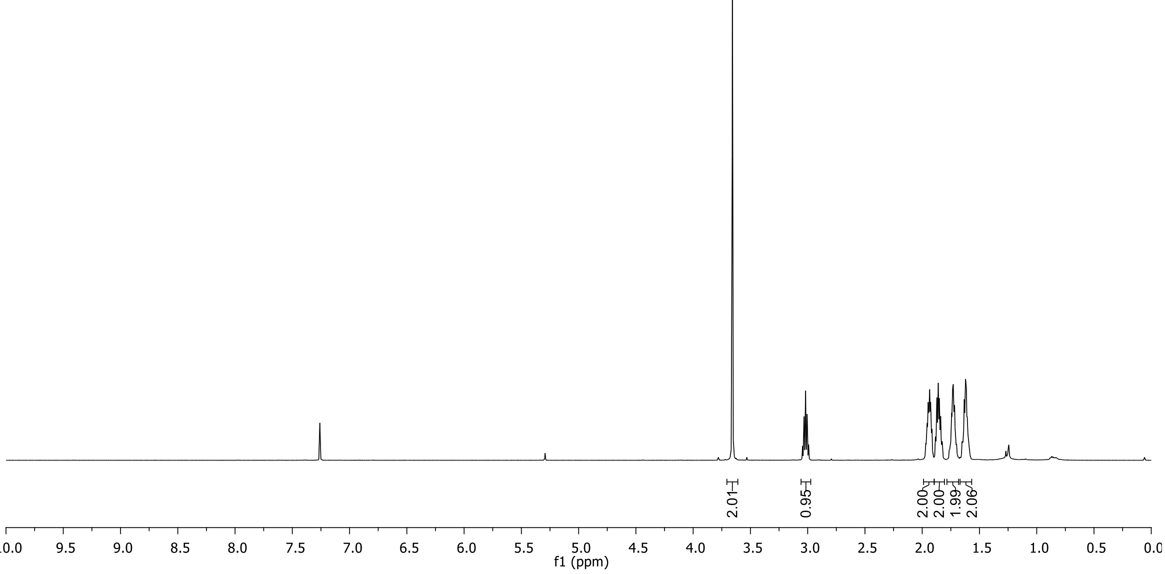

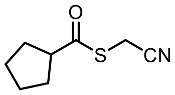

3g

$^{13}C\{^1H\}$ NMR (151 MHz, $CDCl_3$)

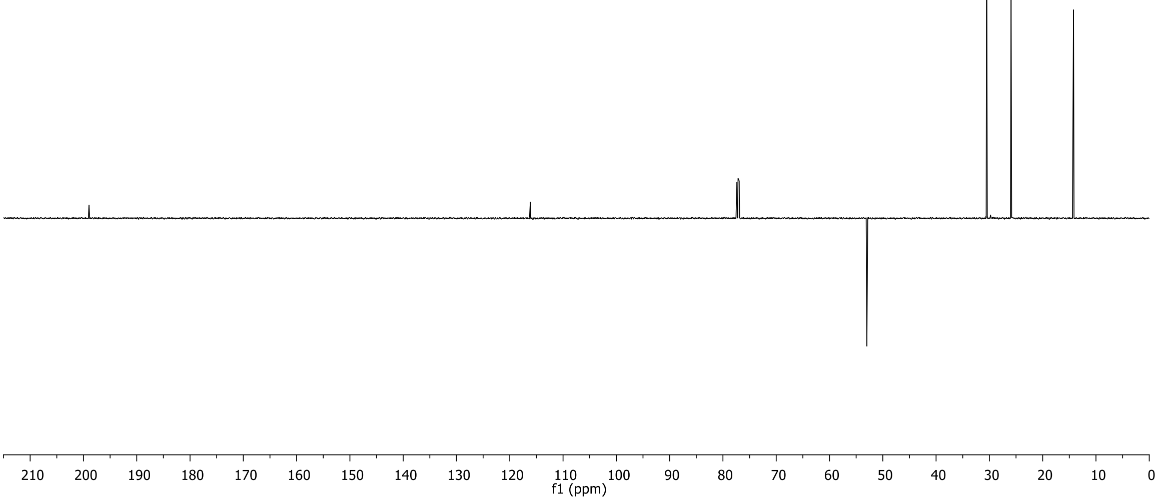

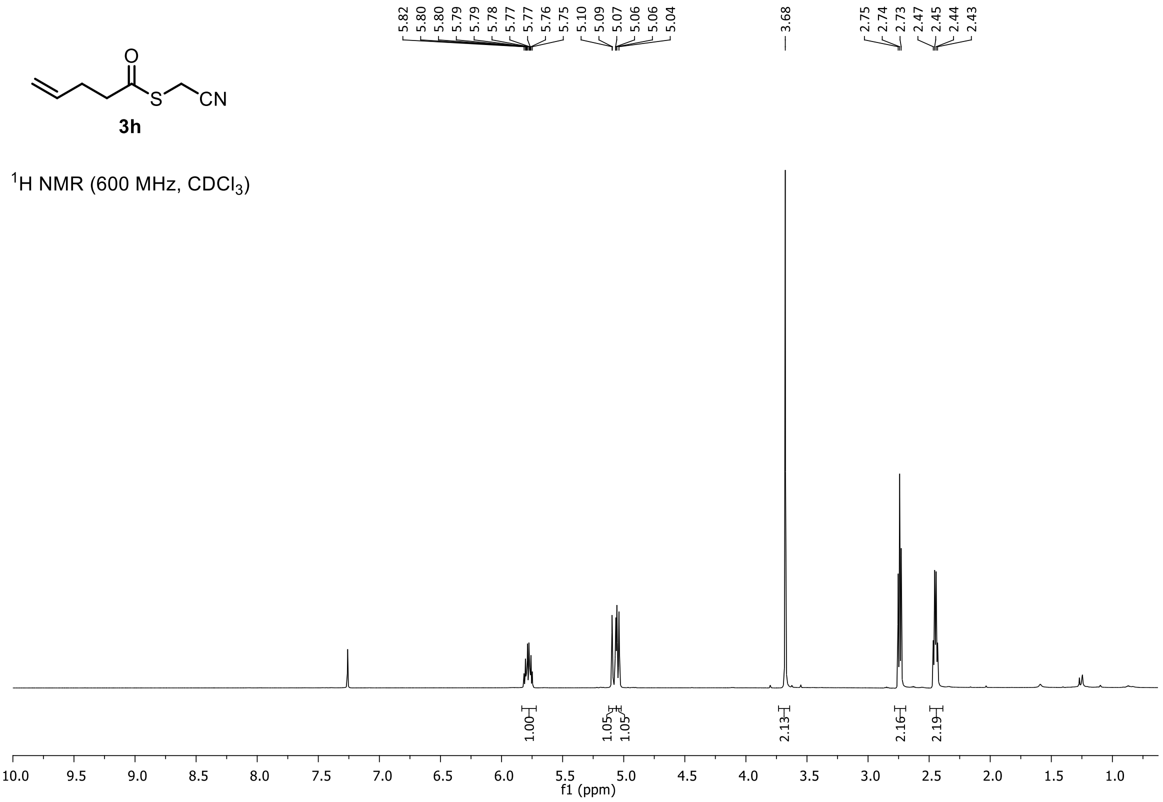
3h
^1H NMR (600 MHz, $CDCl_3$)
f1 (ppm)

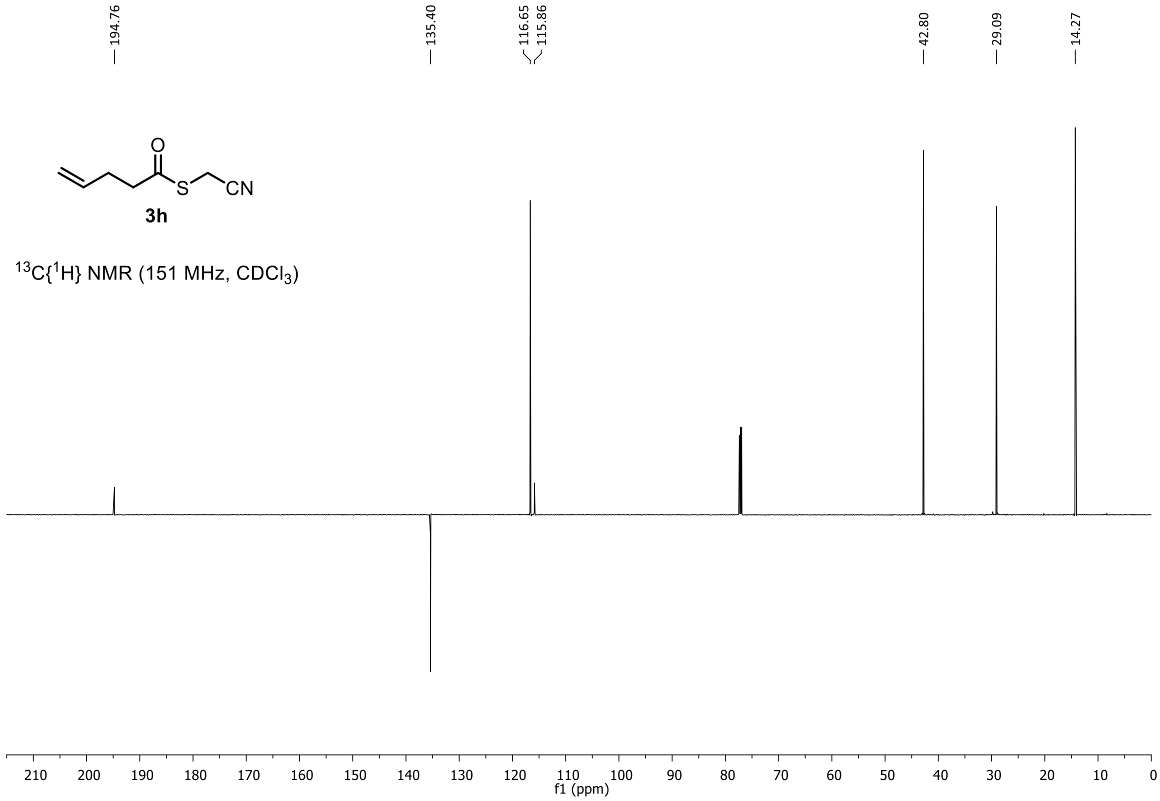
3h
$^{13}C\{^1H\}$ NMR (151 MHz, $CDCl_3$)
f1 (ppm)

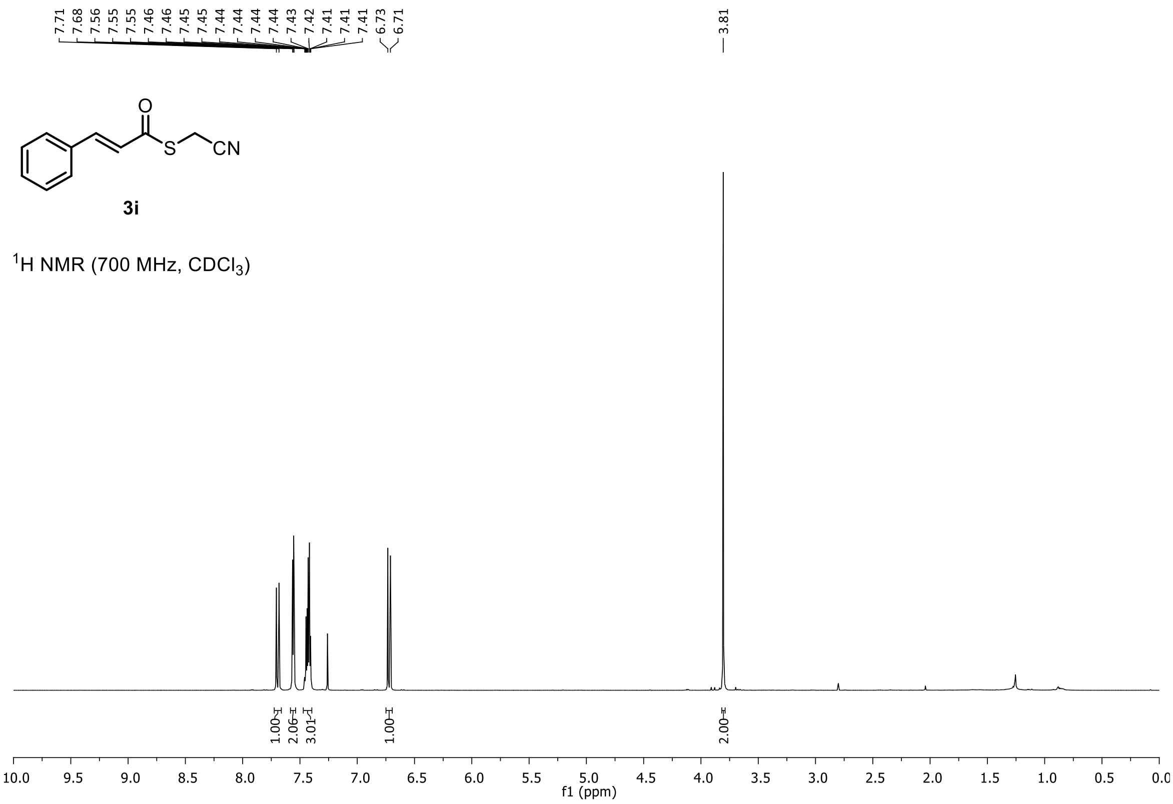
7.71 7.68 7.56 7.55 7.55 7.46 7.46 7.45 7.45 7.44 7.44 7.44 7.44 7.43 7.42 7.41 7.41 7.41 6.73 6.71
3.81
O
S
CN
3i
^{1}H NMR (700 MHz, $CDCl_3$)
1.00 2.06 3.01 1.00 2.00
10.0 9.5 9.0 8.5 8.0 7.5 7.0 6.5 6.0 5.5 5.0 4.5 4.0 3.5 3.0 2.5 2.0 1.5 1.0 0.5 0.0
f1 (ppm)

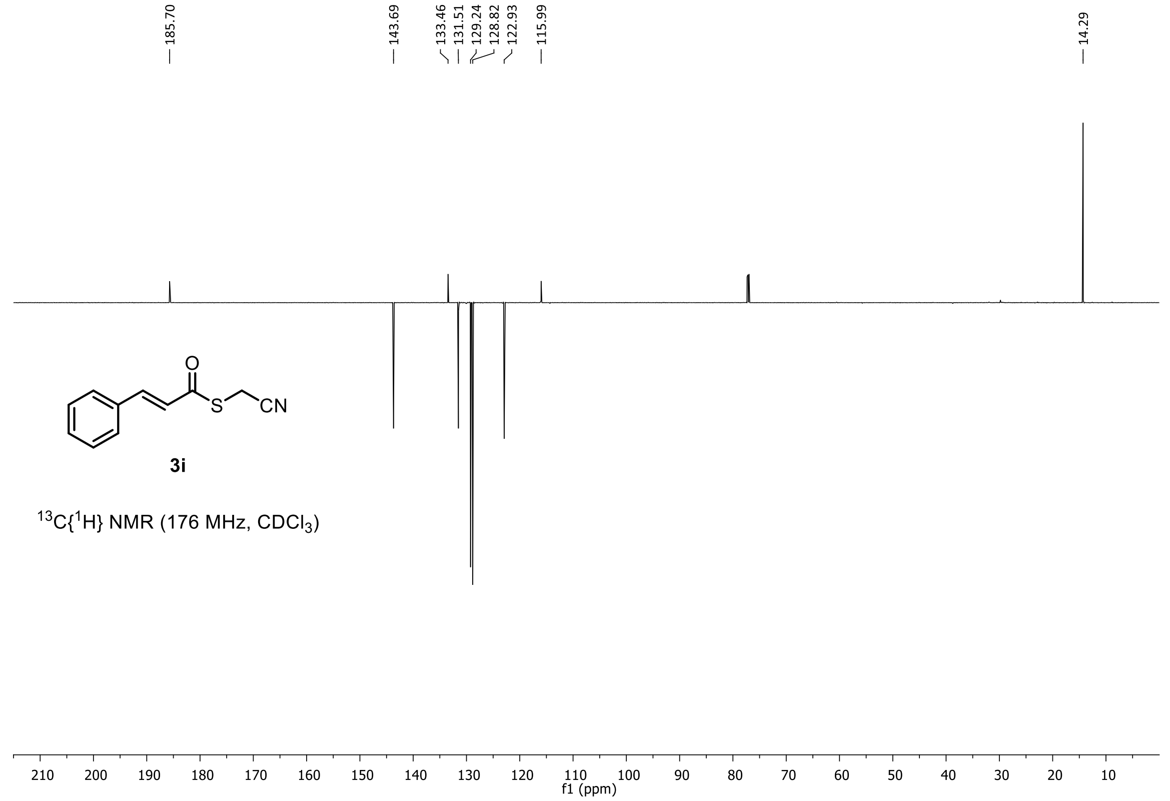
185.70
143.69
133.46 131.51 129.24 128.82 122.93
115.99
14.29
O
S
CN
3i
^{13}C{^{1}H} NMR (176 MHz, $CDCl_3$)
210 200 190 180 170 160 150 140 130 120 110 100 90 80 70 60 50 40 30 20 10
f1 (ppm)

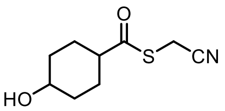

3j major

^{1}H NMR (600 MHz, $CDCl_3$)

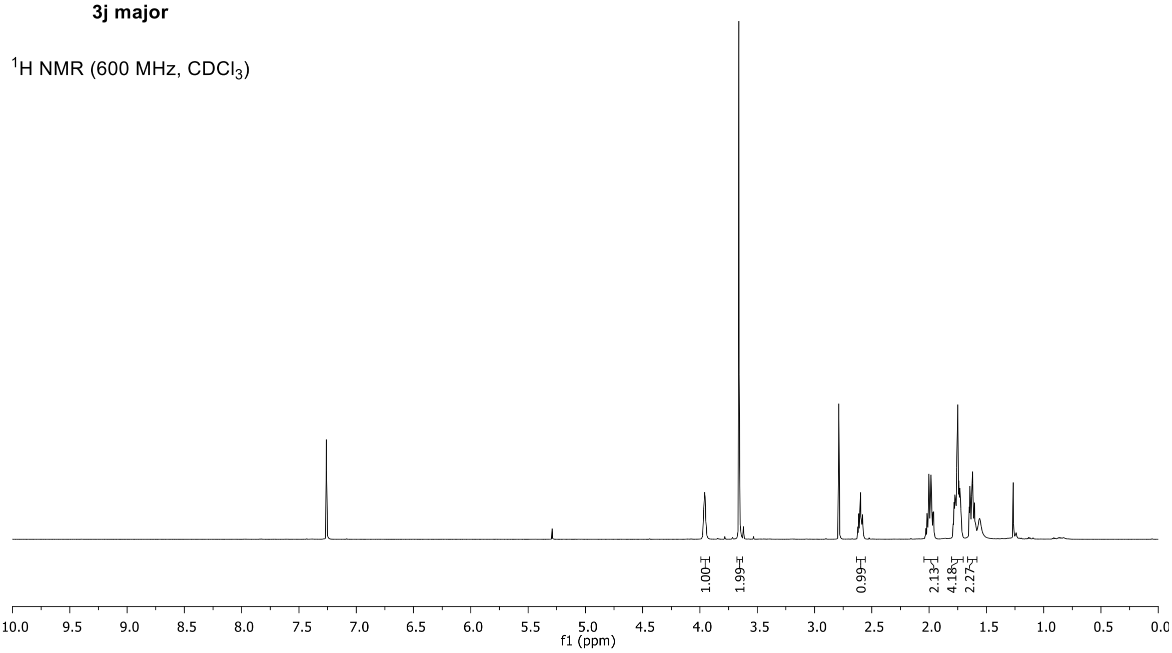

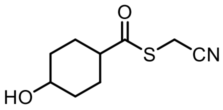

3j major

^{13}C{^{1}H} NMR (151 MHz, $CDCl_3$)

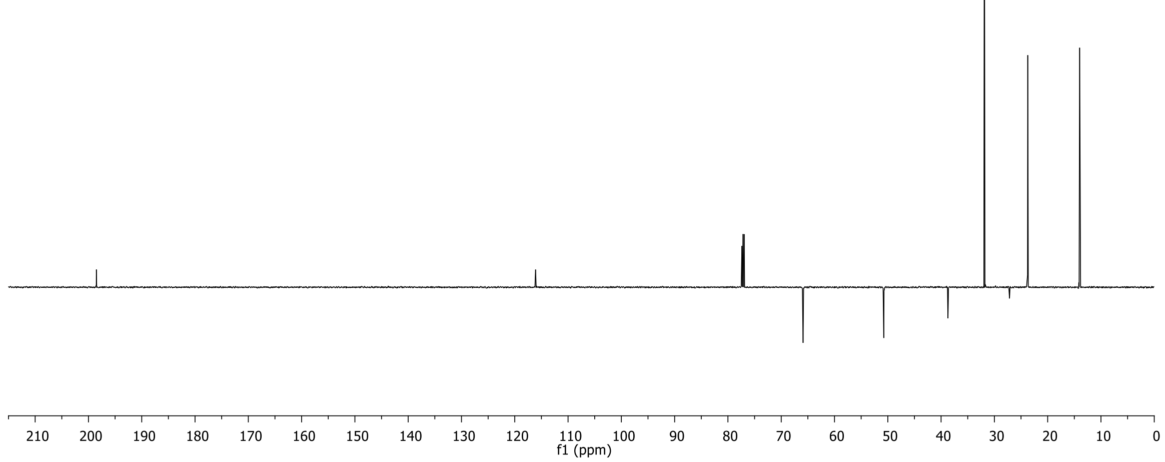

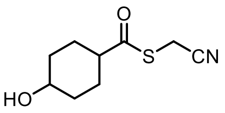

3j minor

^{1}H NMR (600 MHz, $CDCl_3$)

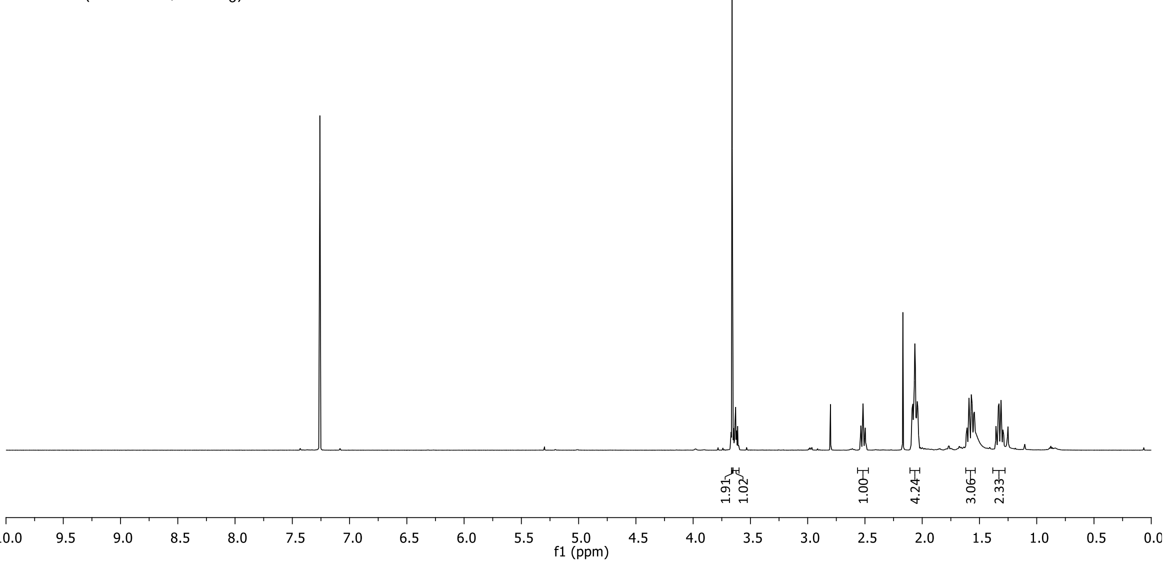

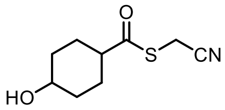

3j minor

$^{13}C\{^{1}H\}$ NMR (151 MHz, $CDCl_3$)

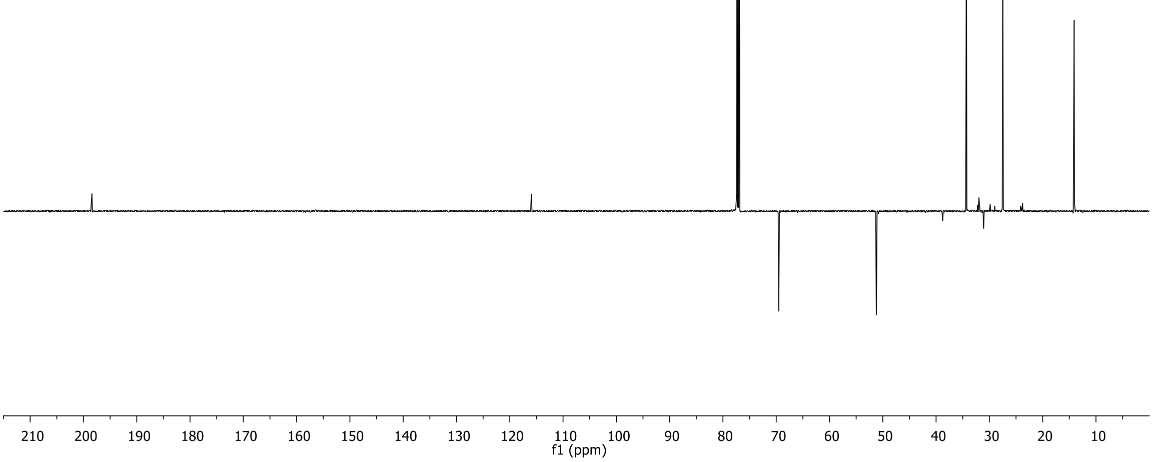

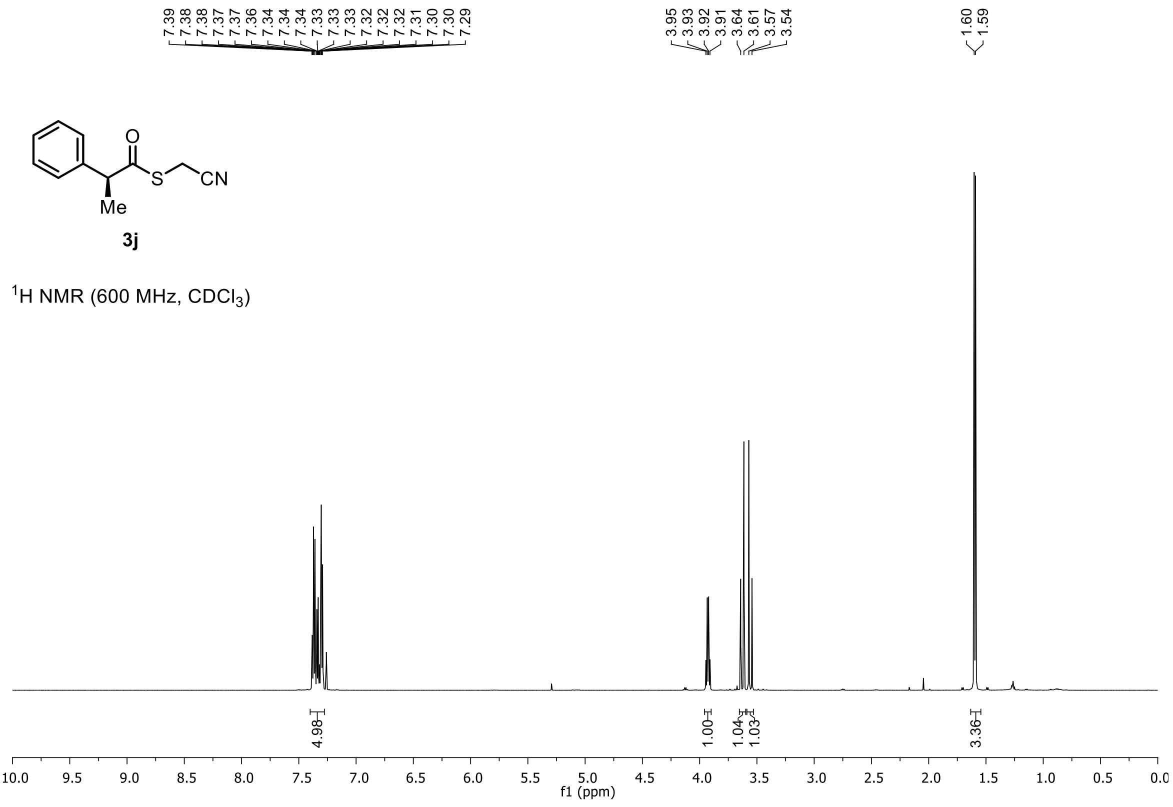
Me
CN
S
O
3j
^{1}H NMR (600 MHz, CDCl$_3$)
7.39 7.38 7.38 7.37 7.37 7.36 7.34 7.34 7.34 7.33 7.33 7.33 7.32 7.32 7.32 7.31 7.30 7.30 7.29
3.95 3.93 3.92 3.91 3.64 3.61 3.57 3.54
1.60 1.59
4.98 1.00 1.04 1.03 3.36
f1 (ppm)

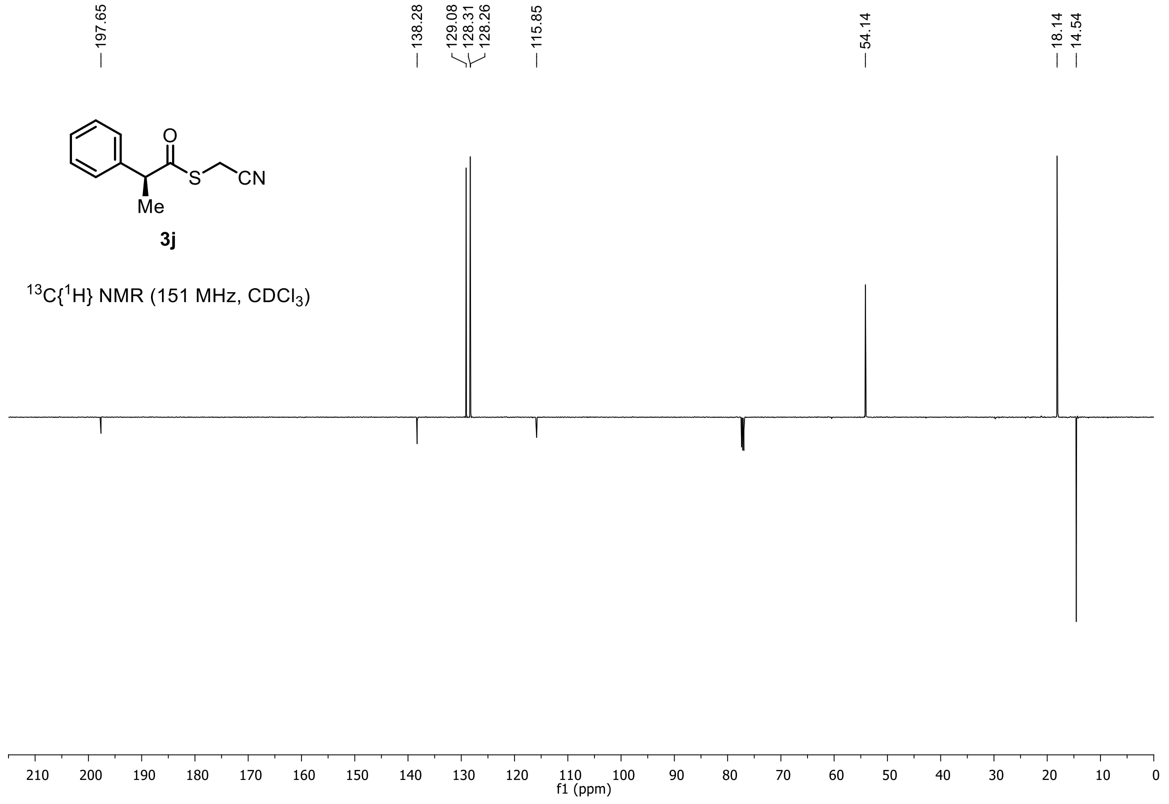
Me
CN
S
O
3j
^{13}C{^{1}H} NMR (151 MHz, CDCl$_3$)
197.65
138.28
129.08 128.31 128.26
115.85
54.14
18.14 14.54
f1 (ppm)

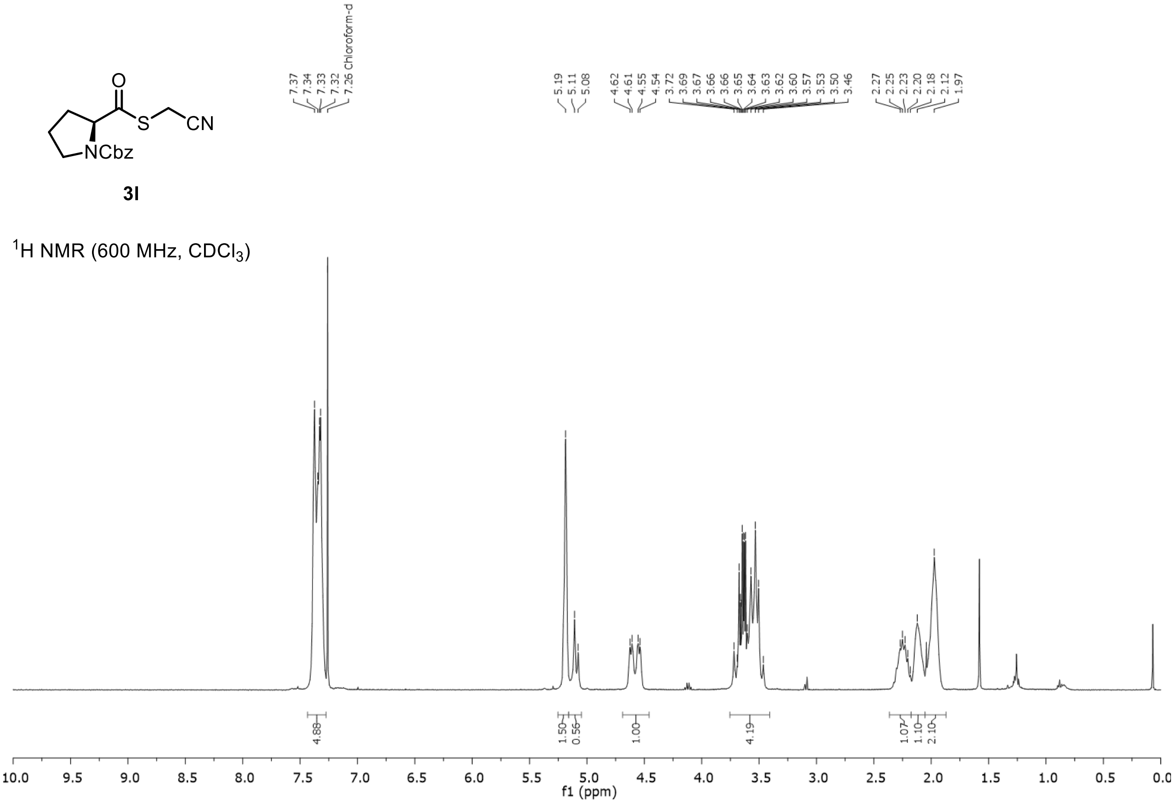
O
S
CN
NCbz
3l
1H NMR (600 MHz, CDCl3)
7.37
7.34
7.33
7.32
7.26 Chloroform-d
5.19
5.11
5.08
4.62
4.61
4.55
4.54
3.72
3.69
3.67
3.66
3.66
3.65
3.64
3.63
3.62
3.60
3.57
3.53
3.50
3.46
2.27
2.25
2.23
2.20
2.18
2.12
1.97
4.88
1.50
0.56
1.00
4.19
1.07
1.10
2.10
10.0 9.5 9.0 8.5 8.0 7.5 7.0 6.5 6.0 5.5 5.0 4.5 4.0 3.5 3.0 2.5 2.0 1.5 1.0 0.5 0.0
f1 (ppm)

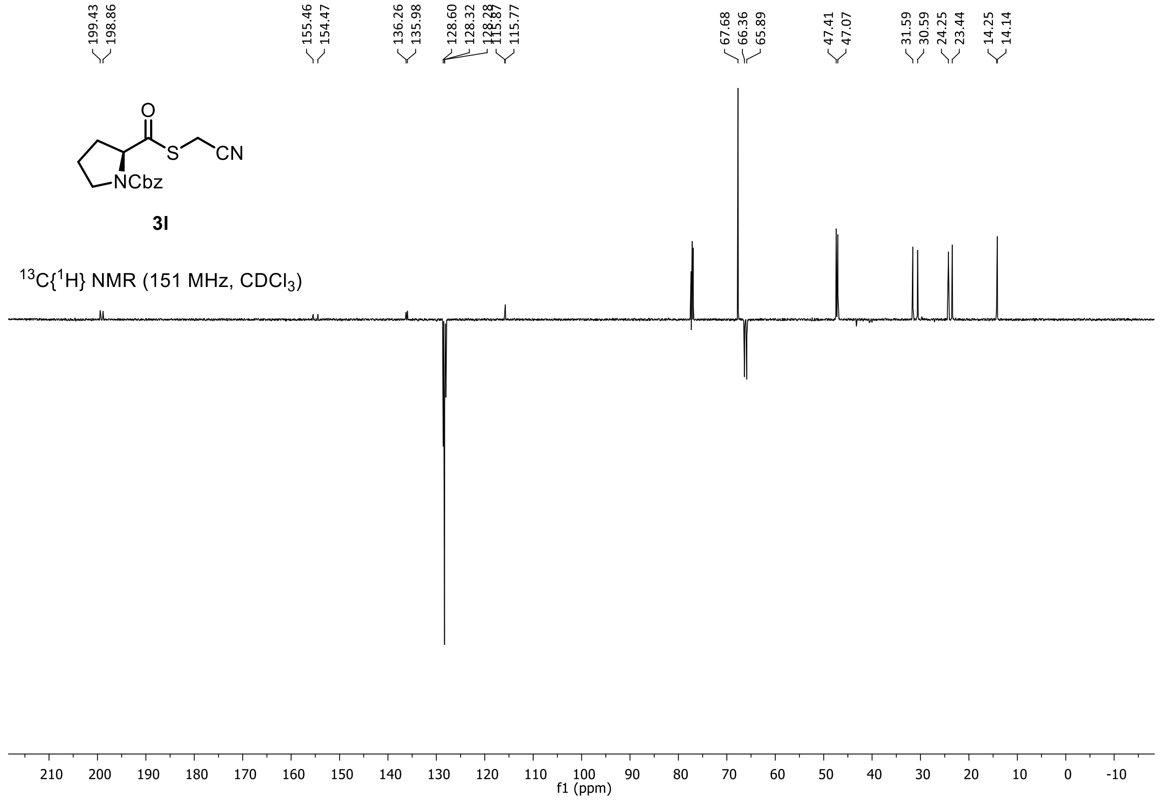
199.43
198.86
155.46
154.47
136.26
135.98
128.60
128.32
128.28
115.87
115.77
67.68
66.36
65.89
47.41
47.07
31.59
30.59
24.25
23.44
14.25
14.14
O
S
CN
NCbz
3l
13C{1H} NMR (151 MHz, CDCl3)
210 200 190 180 170 160 150 140 130 120 110 100 90 80 70 60 50 40 30 20 10 0 -10
f1 (ppm)

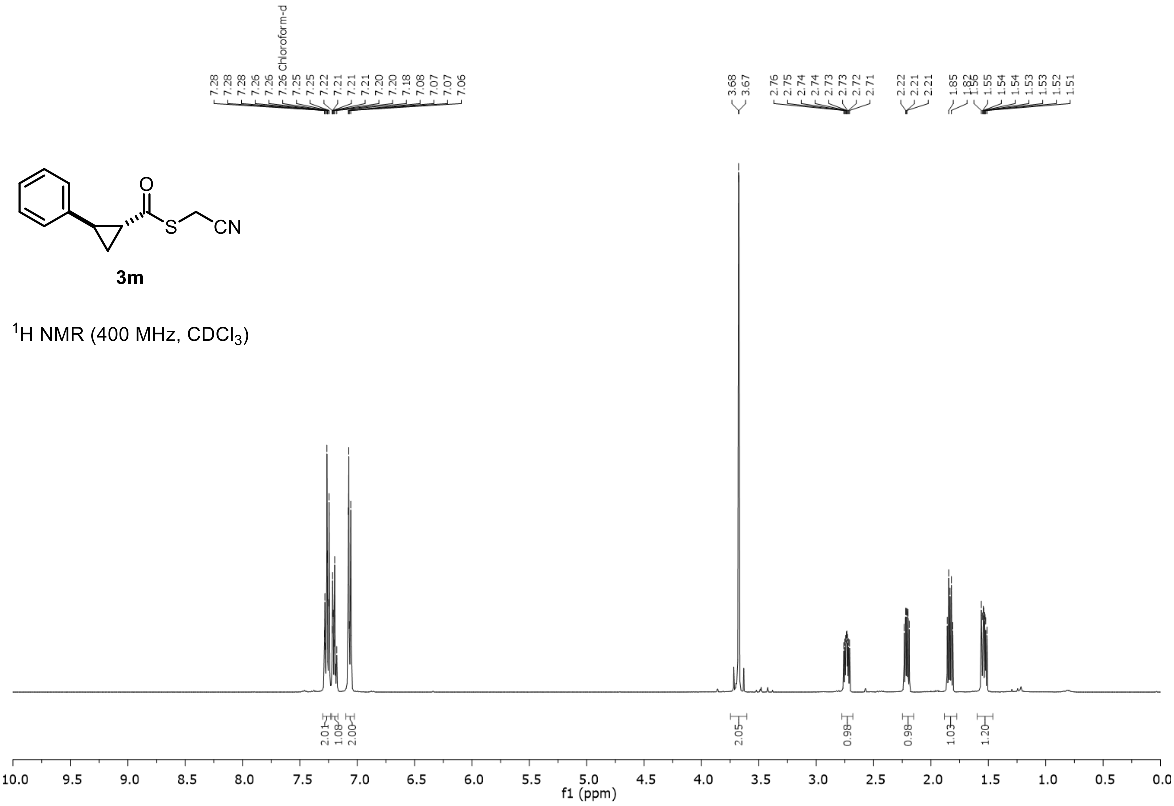
7.28 7.28 7.28 7.26 7.26 Chloroform-d 7.25 7.25 7.22 7.21 7.21 7.21 7.20 7.20 7.18 7.08 7.07 7.07 7.06
3.68 3.67
2.76 2.75 2.74 2.74 2.73 2.73 2.72 2.71
2.22 2.21 2.21
1.85 1.82 1.56 1.55 1.54 1.54 1.53 1.53 1.52 1.51
O
S
CN
3m
^{1}H NMR (400 MHz, CDCl$_3$)
2.01 1.08 2.00 2.05 0.98 0.98 1.03 1.20
f1 (ppm)

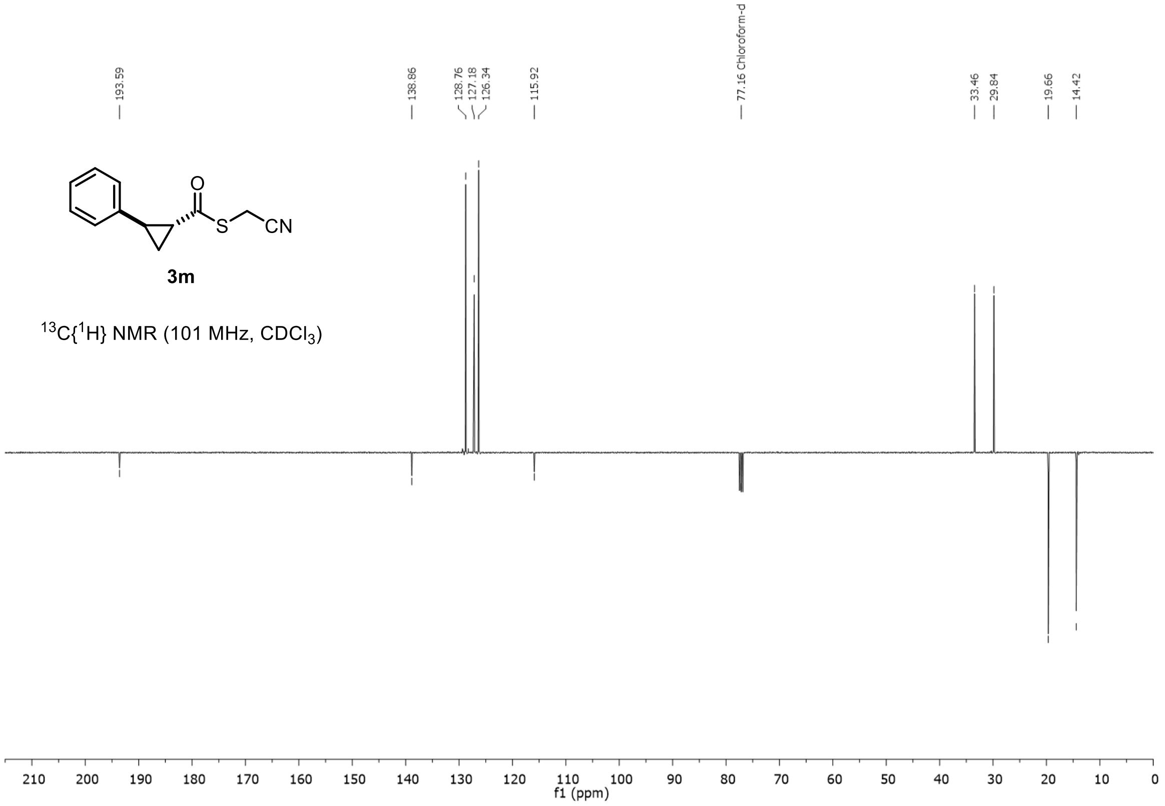
193.59
138.86
128.76 127.18 126.34
115.92
77.16 Chloroform-d
33.46 29.84
19.66 14.42
O
S
CN
3m
^{13}C{^{1}H} NMR (101 MHz, CDCl$_3$)
f1 (ppm)

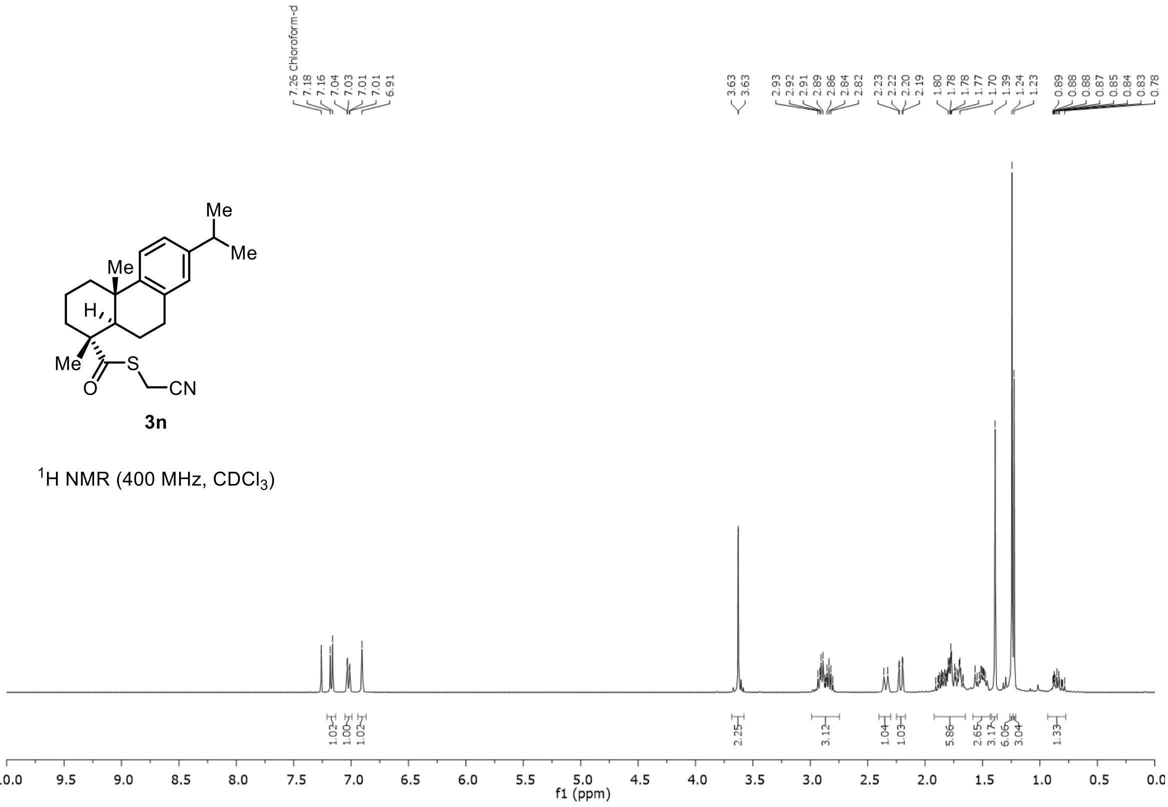
7.26 Chloroform-d
7.18
7.16
7.04
7.03
7.01
7.01
6.91
3.63
3.63
2.93
2.92
2.91
2.89
2.86
2.84
2.82
2.23
2.22
2.20
2.19
1.80
1.78
1.78
1.77
1.70
1.39
1.24
1.23
0.89
0.88
0.88
0.87
0.85
0.84
0.83
0.78
Me
Me
Me
H
Me
O
S
CN
3n
^{1}H NMR (400 MHz, CDCl$_3$)
1.02
1.00
1.02
2.25
3.12
1.04
1.03
5.86
2.65
3.17
6.06
3.04
1.33
f1 (ppm)

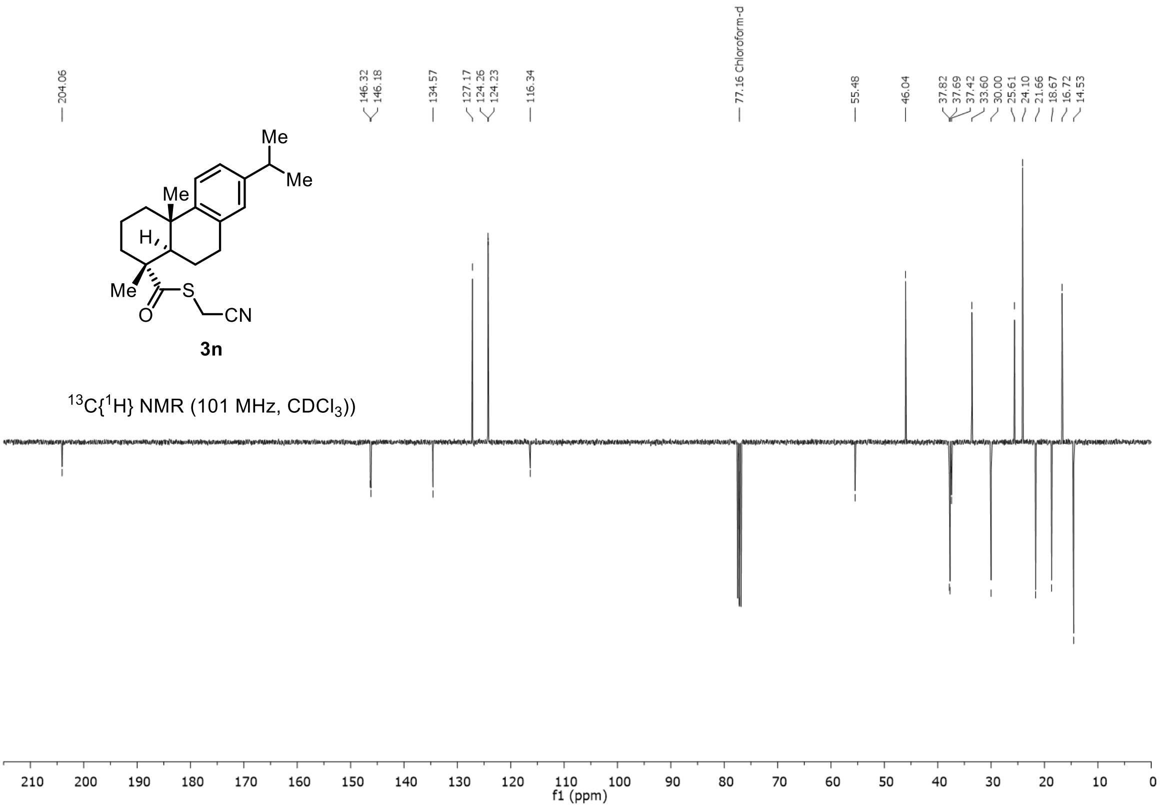
204.06
146.32
146.18
134.57
127.17
124.26
124.23
116.34
77.16 Chloroform-d
55.48
46.04
37.82
37.69
37.42
33.60
30.00
25.61
24.10
21.66
18.67
16.72
14.53
Me
Me
Me
H
Me
O
S
CN
3n
^{13}C{^{1}H} NMR (101 MHz, CDCl$_3$))
f1 (ppm)

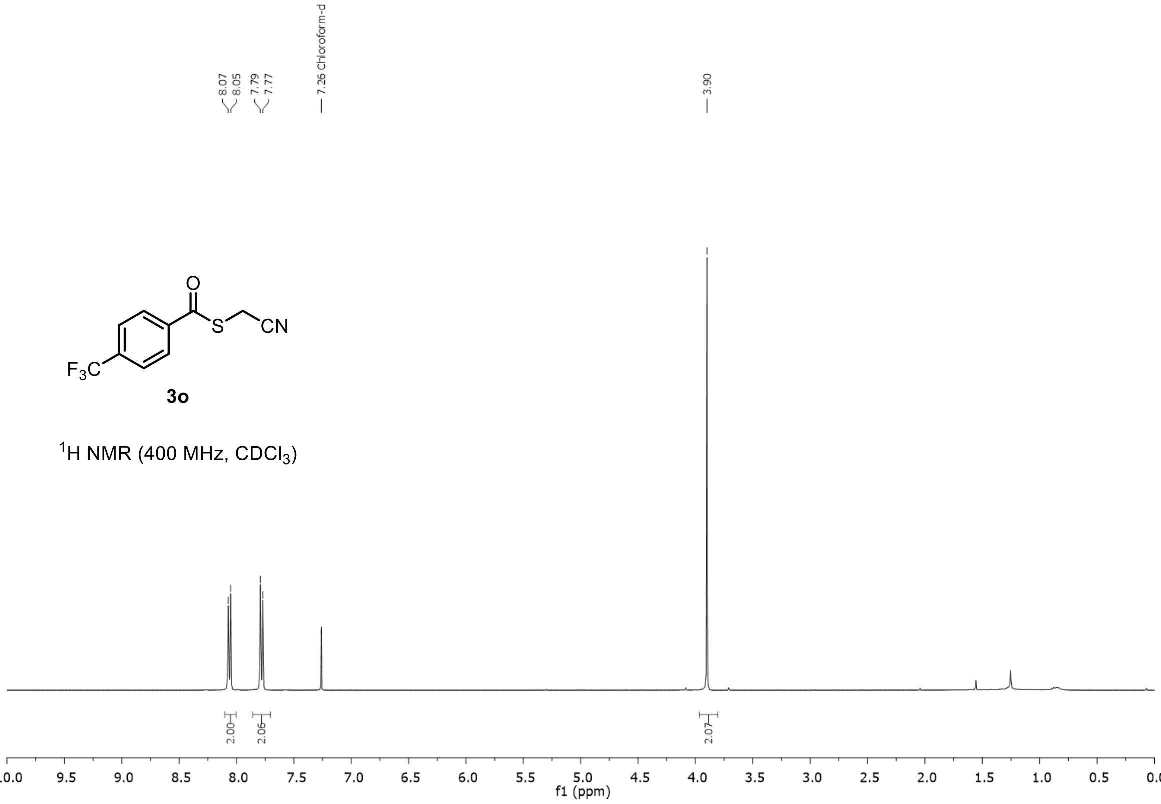
8.07
8.05
7.79
7.77
7.26 Chloroform-d
3.90
O
S
CN
F3C
3o
1H NMR (400 MHz, CDCl3)
2.00
2.06
2.07
10.0 9.5 9.0 8.5 8.0 7.5 7.0 6.5 6.0 5.5 5.0 4.5 4.0 3.5 3.0 2.5 2.0 1.5 1.0 0.5 0.0
f1 (ppm)

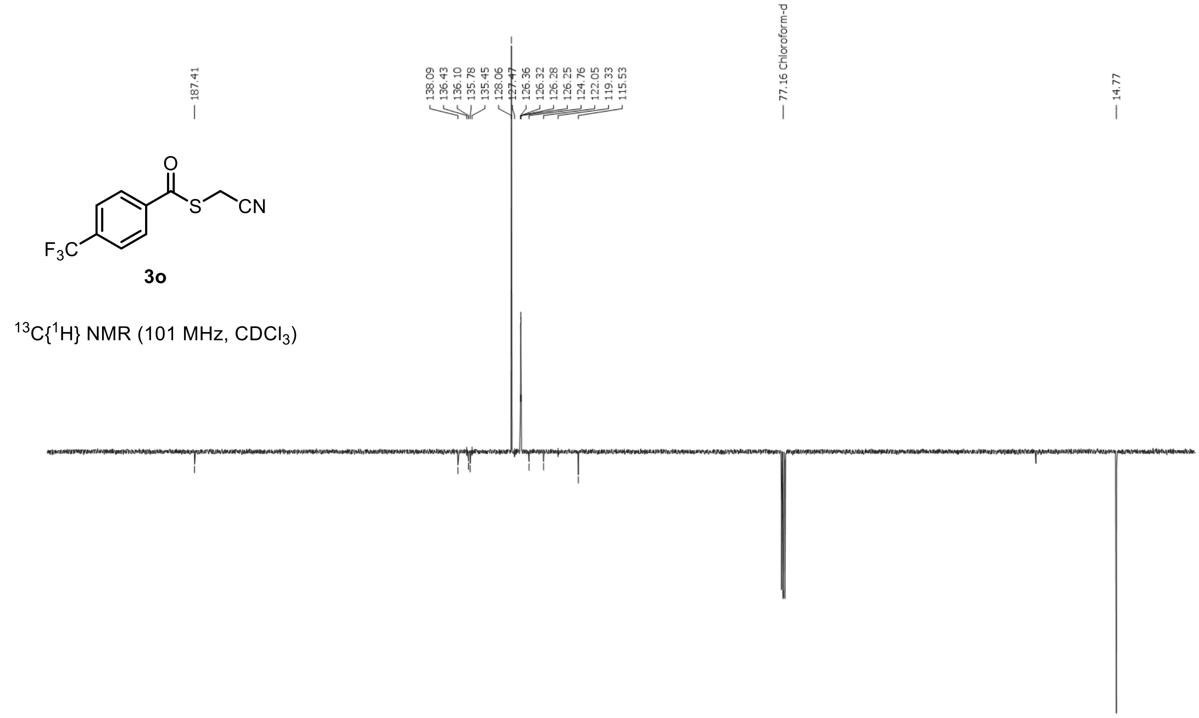
187.41
138.09
136.43
136.10
135.78
135.45
128.06
127.47
126.36
126.32
126.28
126.25
124.76
122.05
119.33
115.53
77.16 Chloroform-d
14.77
O
S
CN
F3C
3o
13C{1H} NMR (101 MHz, CDCl3)
210 200 190 180 170 160 150 140 130 120 110 100 90 80 70 60 50 40 30 20 10 0
f1 (ppm)

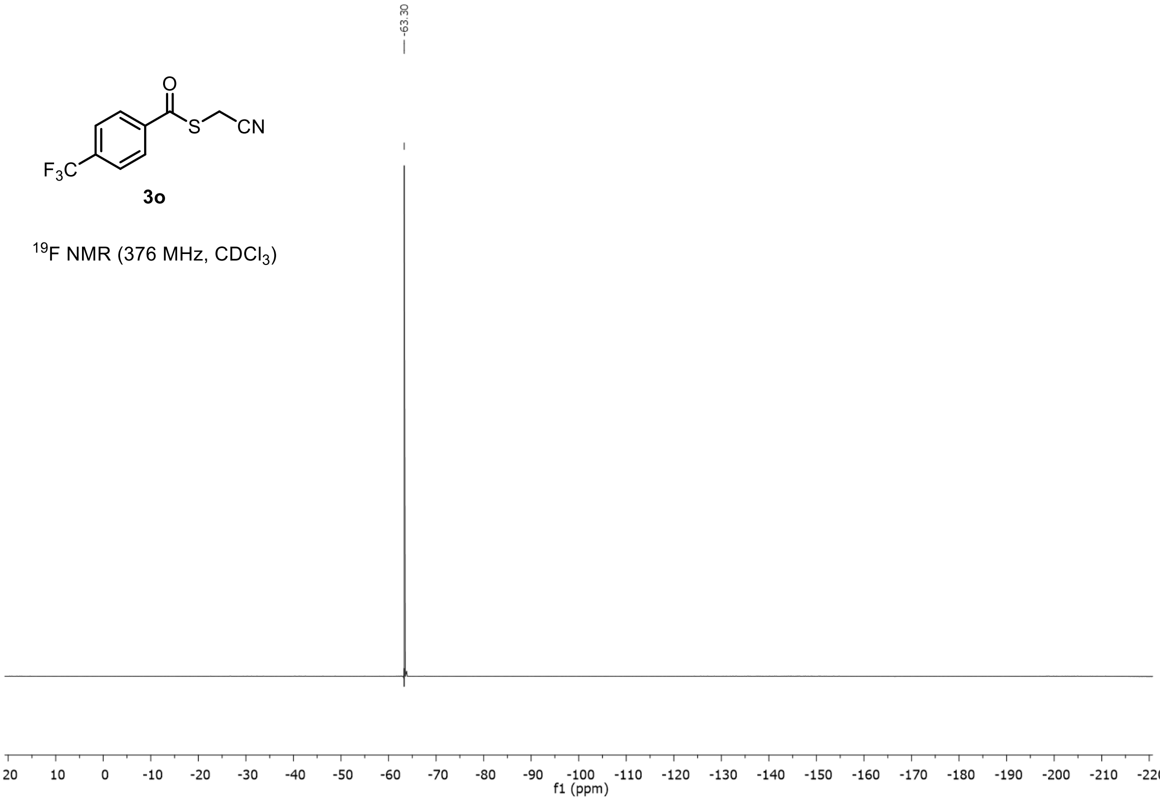
-63.30
O
S
CN
F3C
3o
19F NMR (376 MHz, CDCl3)
20 10 0 -10 -20 -30 -40 -50 -60 -70 -80 -90 -100 -110 -120 -130 -140 -150 -160 -170 -180 -190 -200 -210 -220
f1 (ppm)

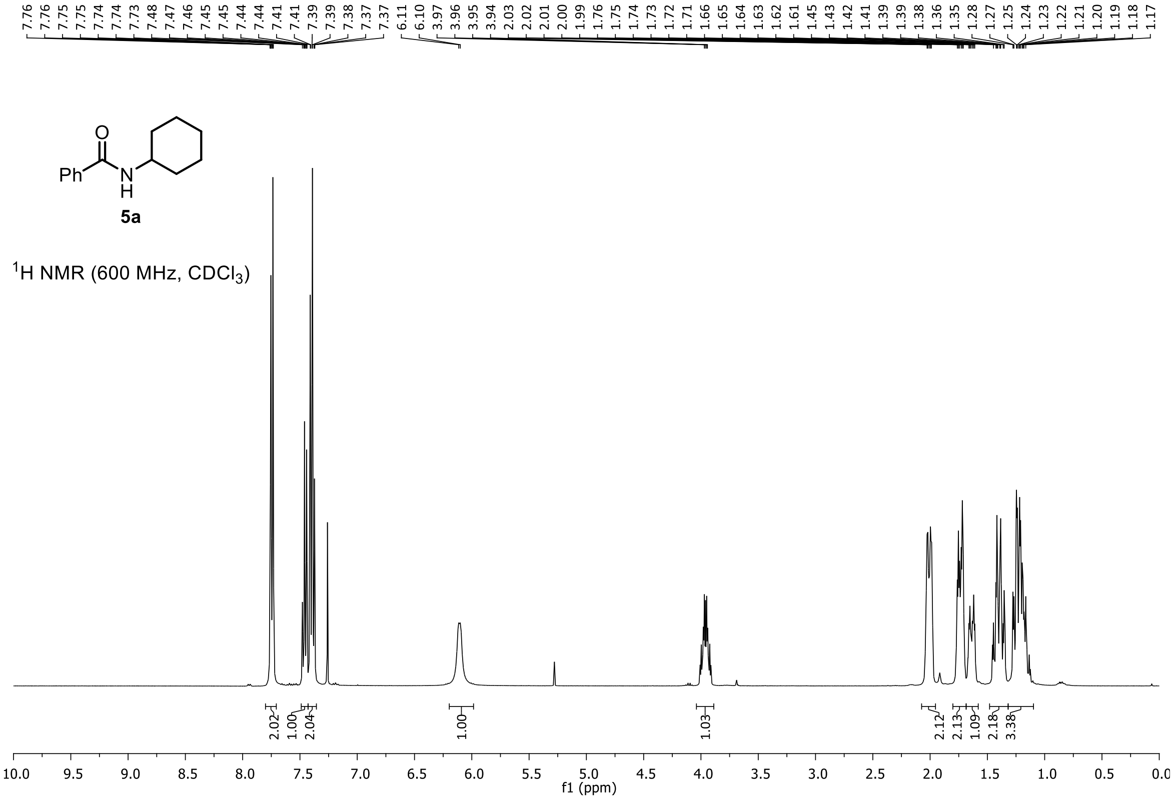
7.76 7.76 7.75 7.75 7.74 7.74 7.73 7.48 7.47 7.46 7.45 7.45 7.44 7.44 7.41 7.41 7.39 7.39 7.38 7.37 7.37 6.11 6.10 3.97 3.96 3.95 3.94 2.03 2.02 2.01 2.00 1.99 1.76 1.75 1.74 1.73 1.72 1.71 1.66 1.65 1.64 1.63 1.62 1.61 1.45 1.43 1.42 1.41 1.39 1.39 1.38 1.36 1.35 1.28 1.27 1.25 1.24 1.23 1.22 1.21 1.20 1.19 1.18 1.17
Ph
O
N
H
5a
1H NMR (600 MHz, CDCl3)
2.02 1.00 2.04 1.00 1.03 2.12 2.13 1.09 2.18 3.38
f1 (ppm)

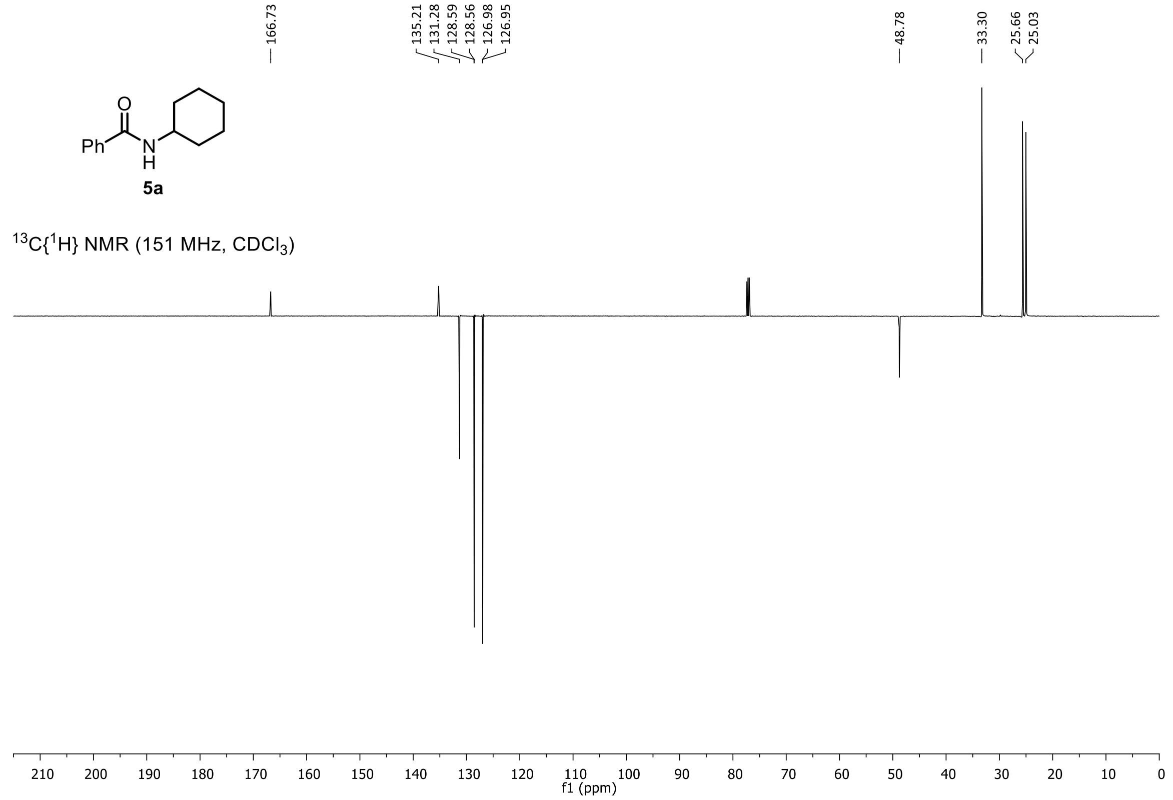
166.73
135.21 131.28 128.59 128.56 126.98 126.95
48.78
33.30
25.66 25.03
Ph
O
N
H
5a
13C{1H} NMR (151 MHz, CDCl3)
f1 (ppm)

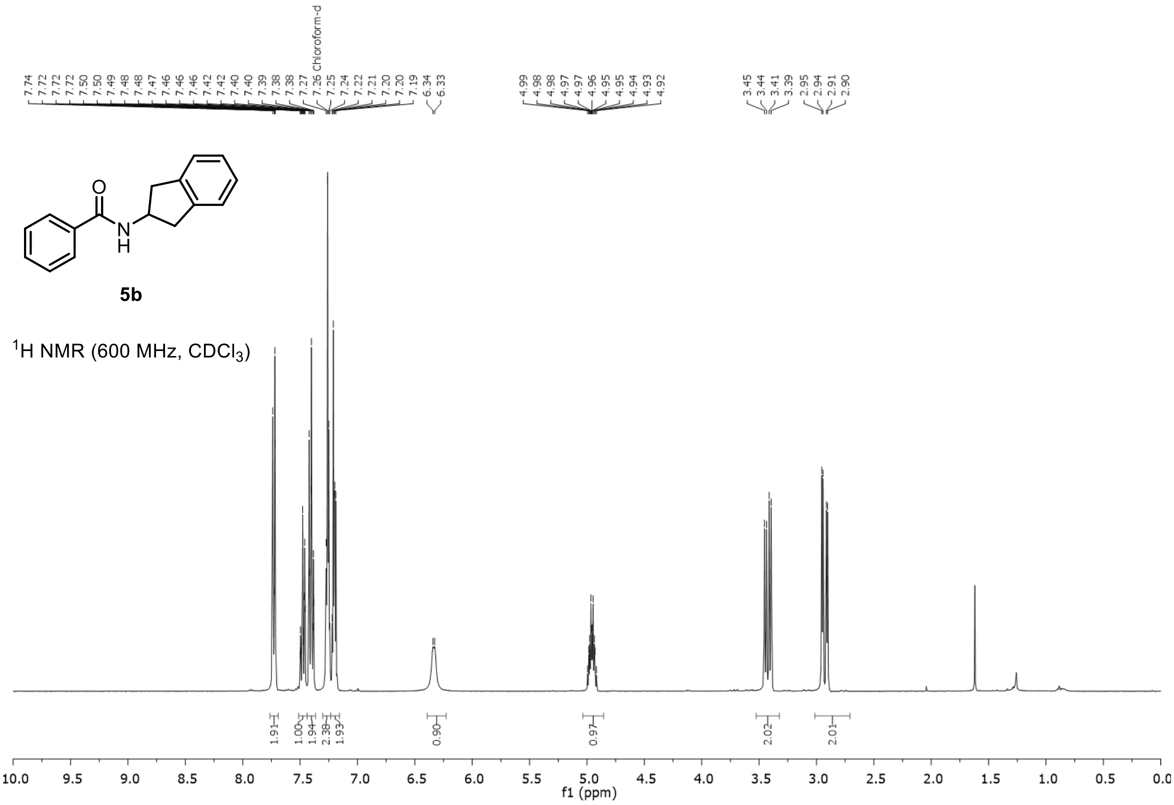
7.74
7.72
7.72
7.50
7.49
7.48
7.48
7.47
7.46
7.46
7.46
7.42
7.42
7.40
7.40
7.39
7.38
7.38
7.27
7.26 Chloroform-d
7.25
7.24
7.22
7.21
7.20
7.20
7.19
6.34
6.33
4.99
4.98
4.98
4.97
4.97
4.96
4.95
4.95
4.94
4.93
4.92
3.45
3.44
3.41
3.39
2.95
2.94
2.91
2.90
O
N
H
5b
1H NMR (600 MHz, CDCl3)
1.91
1.00
1.94
2.38
1.93
0.90
0.97
2.02
2.01
f1 (ppm)

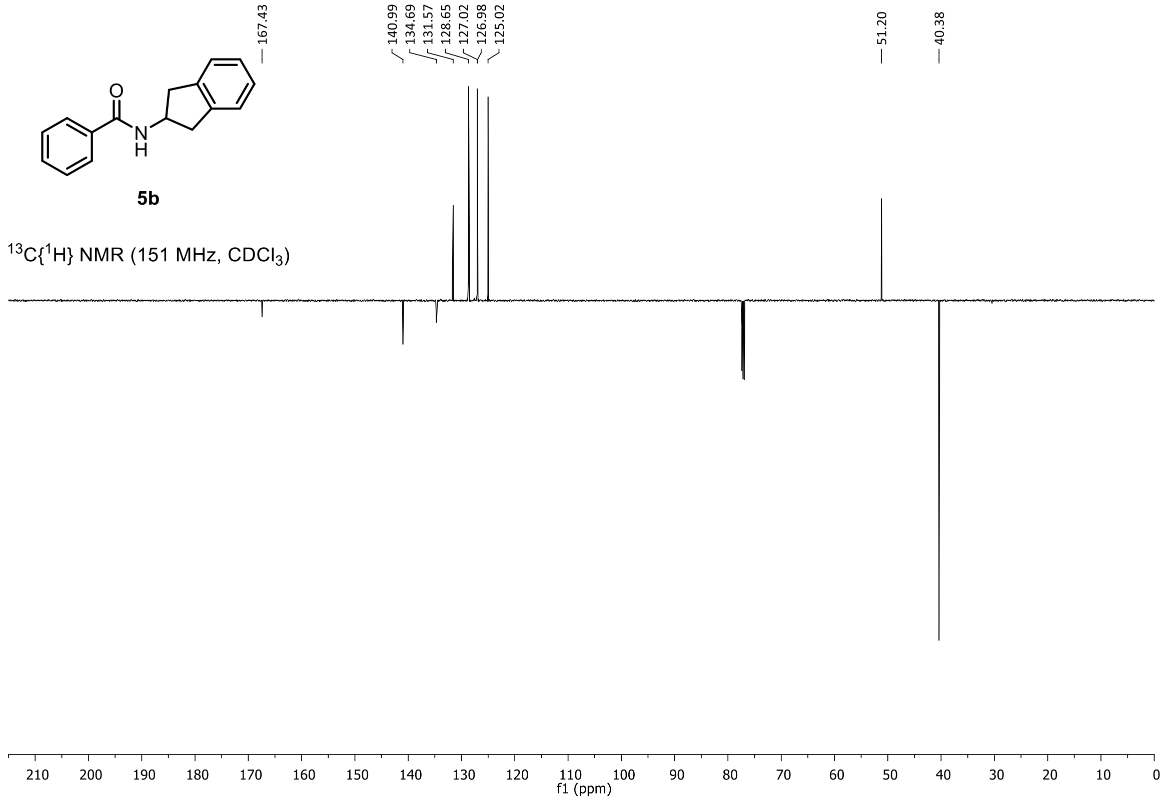
167.43
140.99
134.69
131.57
128.65
127.02
126.98
125.02
51.20
40.38
O
N
H
5b
13C{1H} NMR (151 MHz, CDCl3)
f1 (ppm)

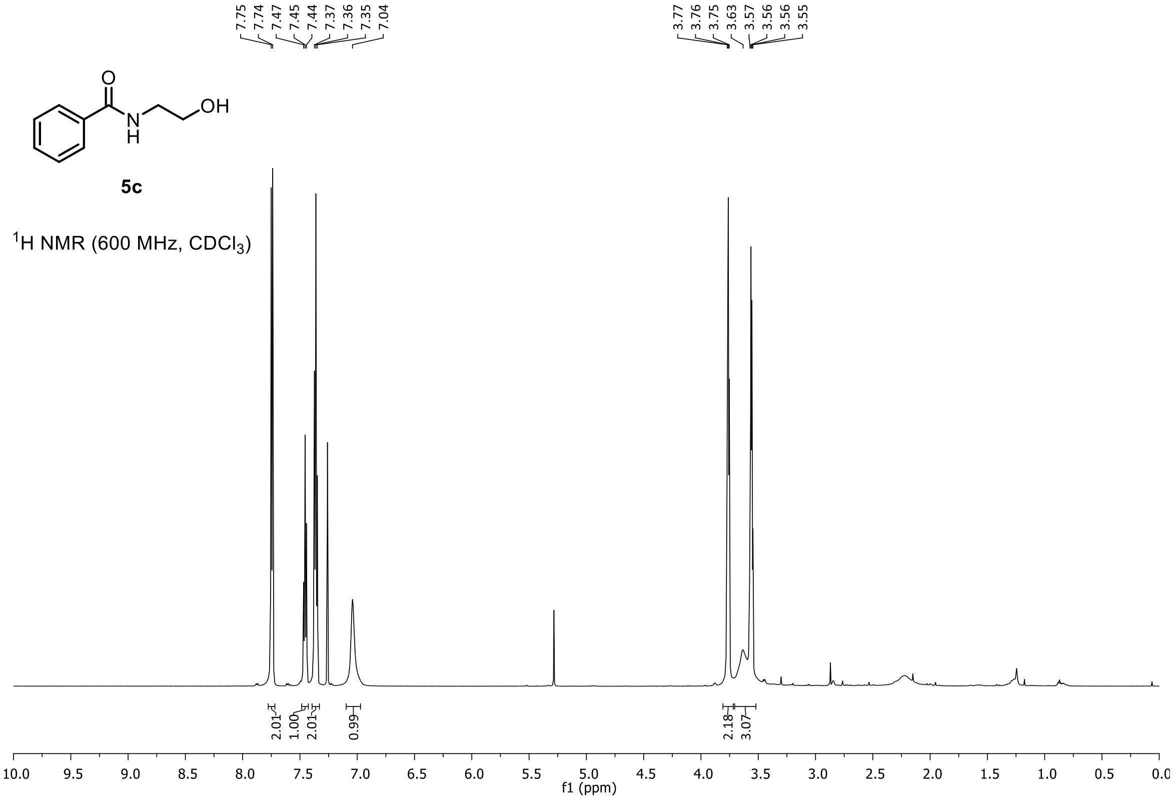
7.75
7.74
7.47
7.45
7.44
7.37
7.36
7.35
7.04
3.77
3.76
3.75
3.63
3.57
3.56
3.56
3.55
O
N
H
OH
5c
^{1}H NMR (600 MHz, CDCl$_3$)
2.01
1.00
2.01
0.99
2.18
3.07
10.0 9.5 9.0 8.5 8.0 7.5 7.0 6.5 6.0 5.5 5.0 4.5 4.0 3.5 3.0 2.5 2.0 1.5 1.0 0.5 0.0
f1 (ppm)

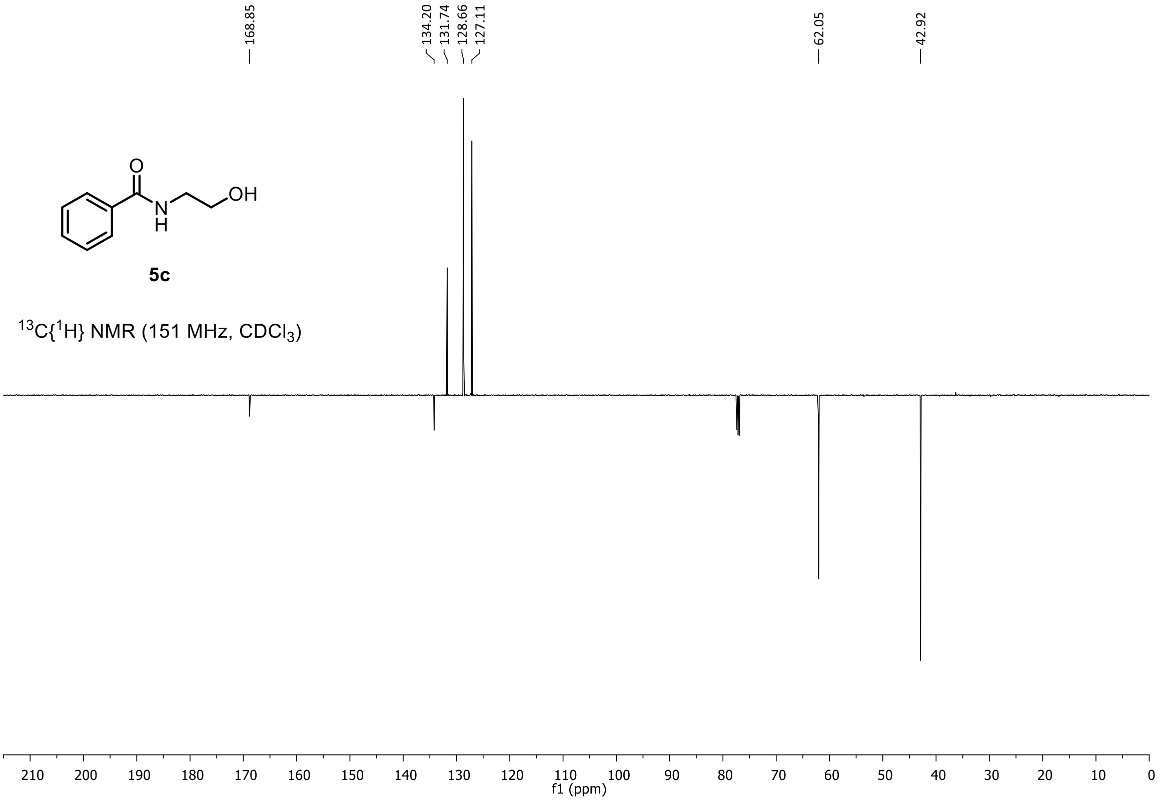
168.85
134.20
131.74
128.66
127.11
62.05
42.92
O
N
H
OH
5c
^{13}C{^{1}H} NMR (151 MHz, CDCl$_3$)
210 200 190 180 170 160 150 140 130 120 110 100 90 80 70 60 50 40 30 20 10 0
f1 (ppm)

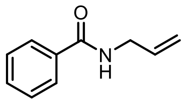

5d

^{1}H NMR (600 MHz, $CDCl_3$)

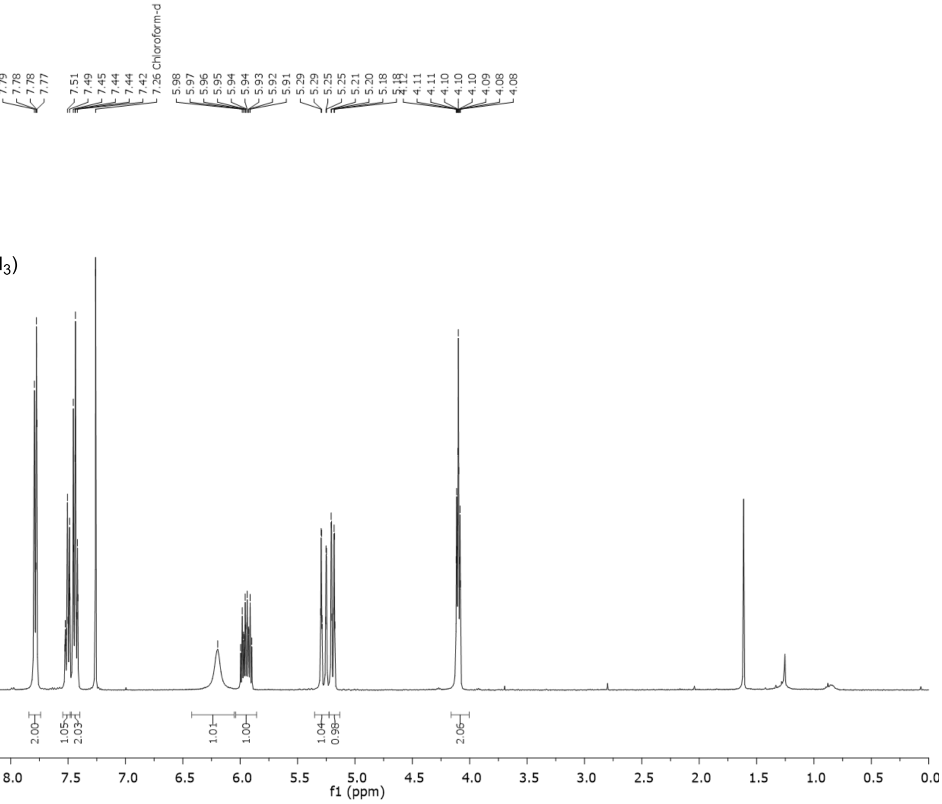

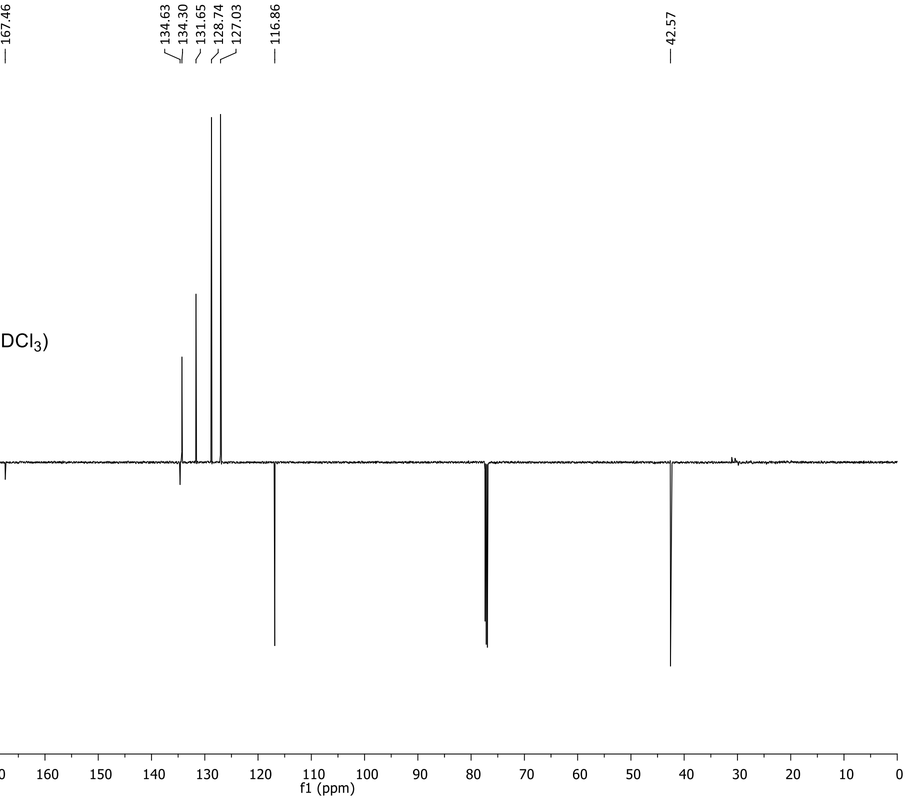

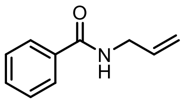

5d

$^{13}C\{^1H\}$ NMR (151 MHz, $CDCl_3$)

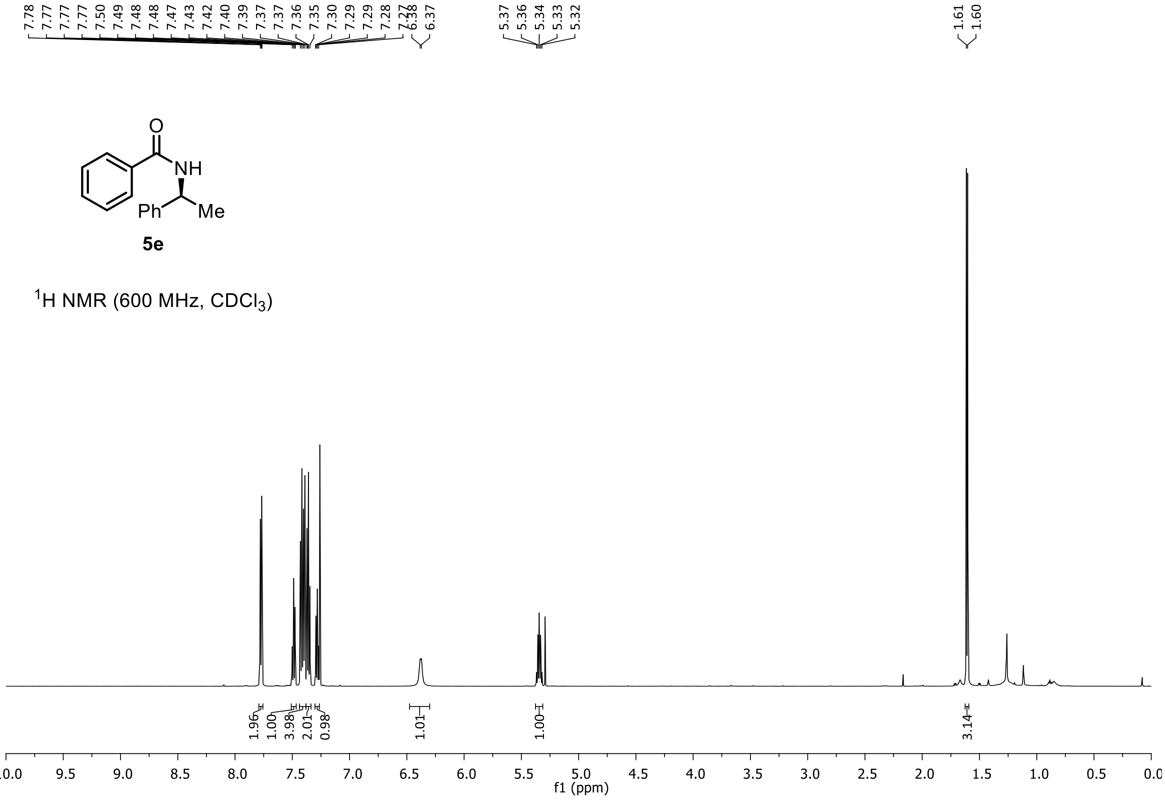
7.78 7.77 7.77 7.77 7.50 7.49 7.48 7.48 7.47 7.43 7.42 7.40 7.39 7.37 7.37 7.36 7.35 7.30 7.29 7.29 7.28 7.27 6.38 6.37
5.37 5.36 5.34 5.33 5.32
1.61 1.60
5e
^{1}H NMR (600 MHz, $CDCl_3$)
1.96 1.00 3.98 2.01 0.98 1.01 1.00 3.14
10.0 9.5 9.0 8.5 8.0 7.5 7.0 6.5 6.0 5.5 5.0 4.5 4.0 3.5 3.0 2.5 2.0 1.5 1.0 0.5 0.0
f1 (ppm)

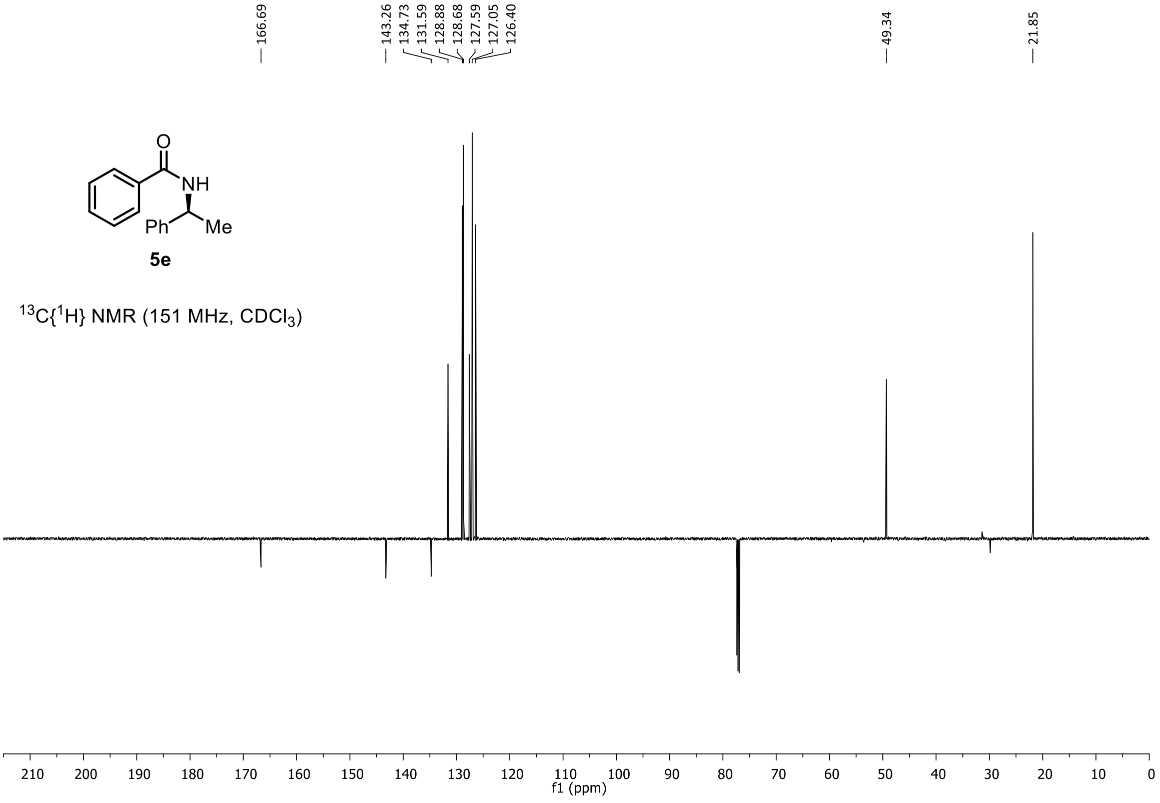
166.69
143.26 134.73 131.59 128.88 128.68 127.59 127.05 126.40
49.34
21.85
5e
^{13}C{^{1}H} NMR (151 MHz, $CDCl_3$)
210 200 190 180 170 160 150 140 130 120 110 100 90 80 70 60 50 40 30 20 10 0
f1 (ppm)

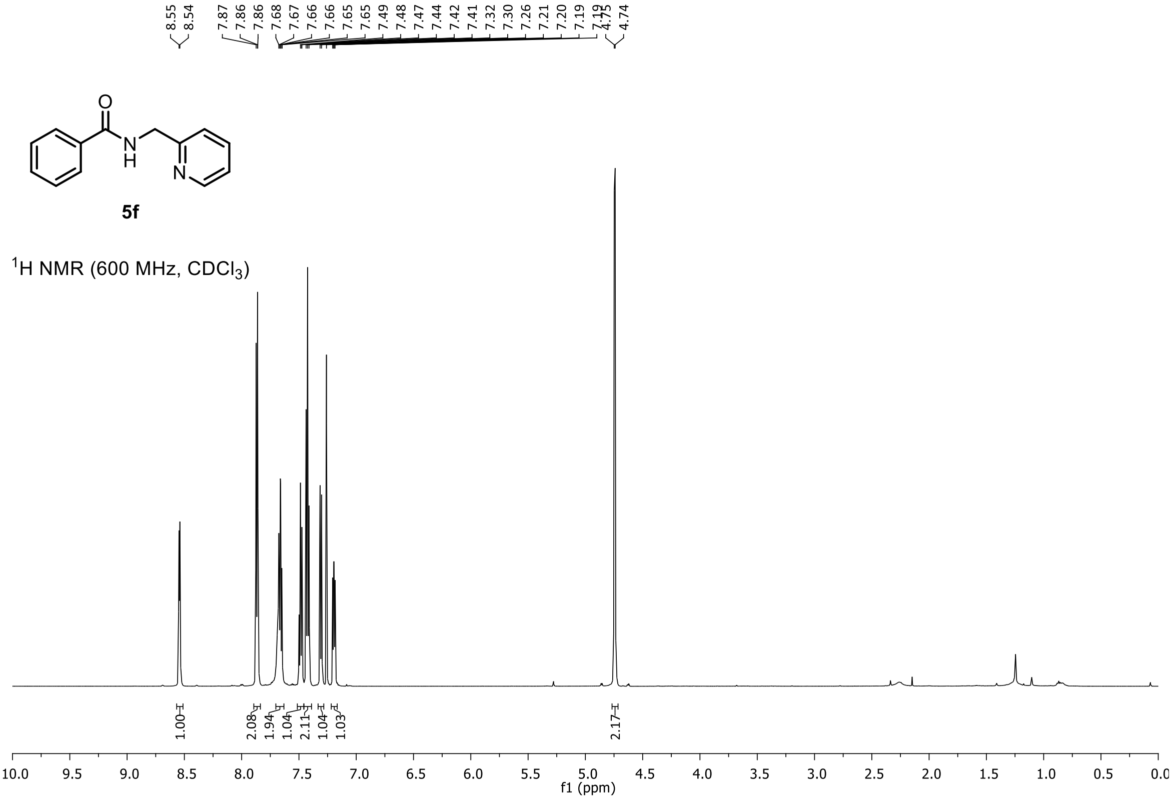
5f
1H NMR (600 MHz, CDCl3)
f1 (ppm)

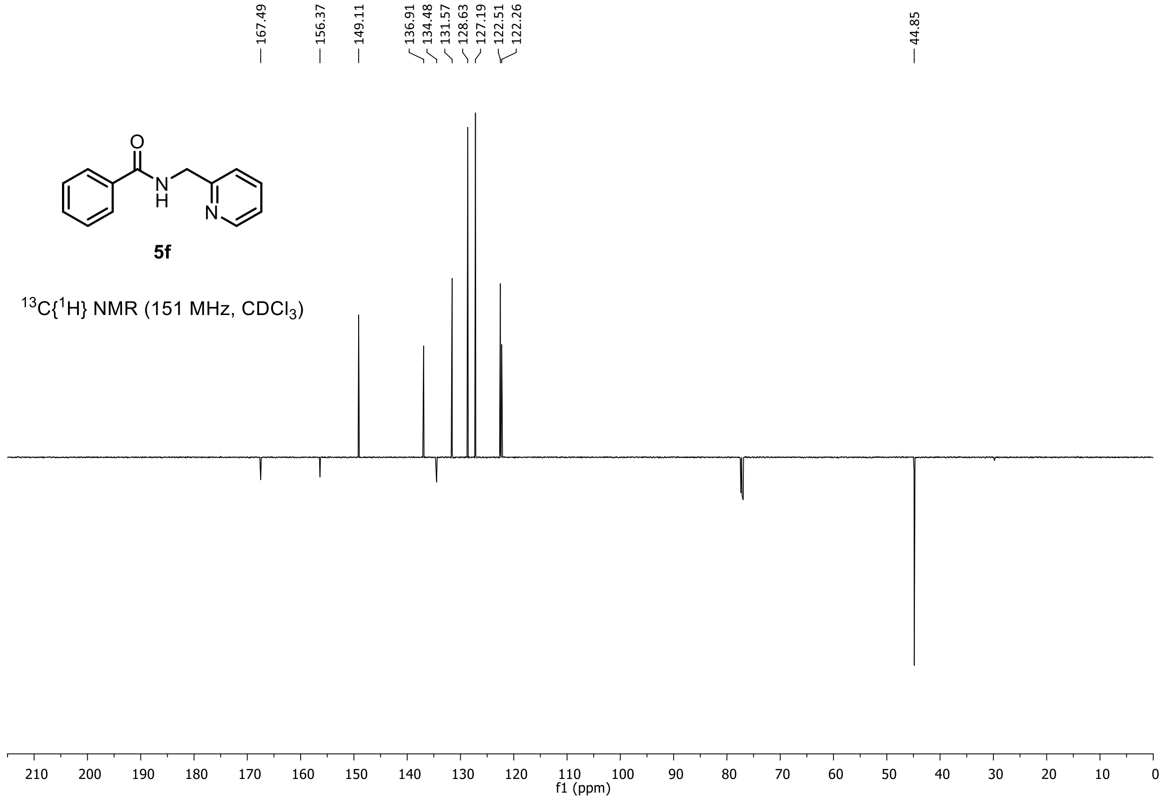
5f
13C{1H} NMR (151 MHz, CDCl3)
f1 (ppm)

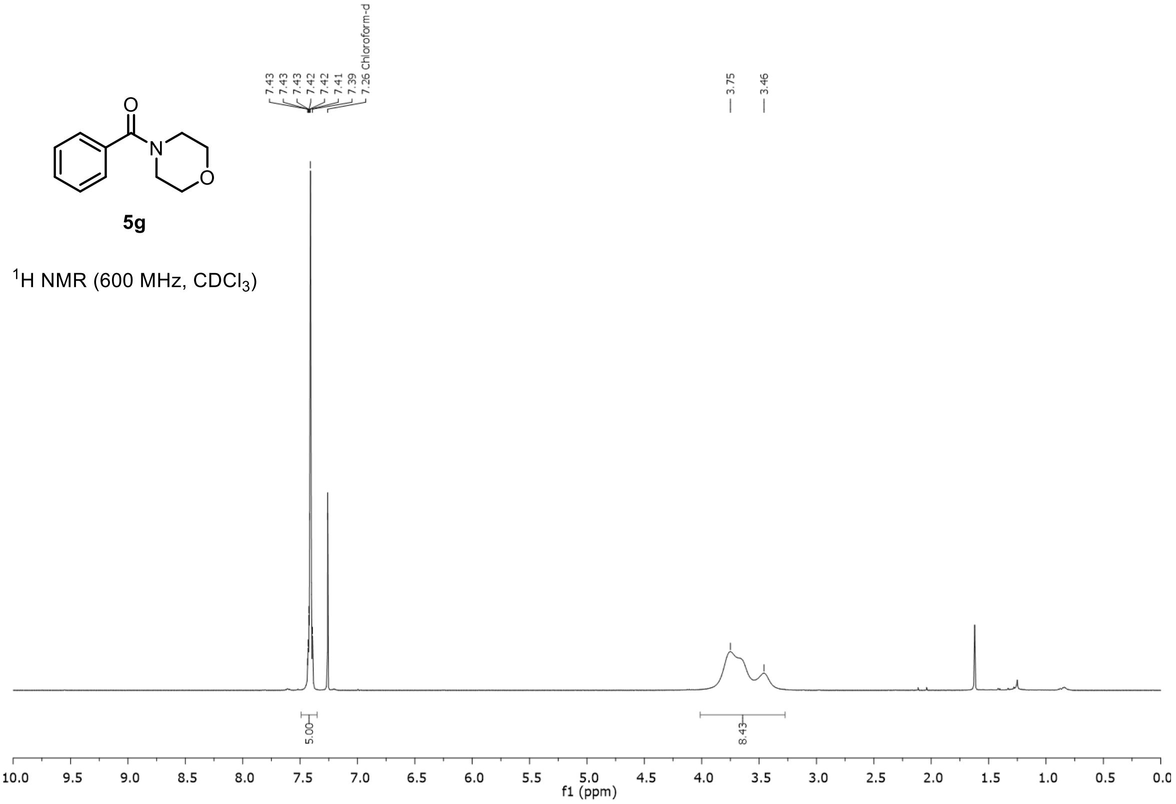
7.43
7.43
7.43
7.42
7.42
7.41
7.39
7.26 Chloroform-d
3.75
3.46
5g
^{1}H NMR (600 MHz, $CDCl_3$)
5.00
8.43
10.0 9.5 9.0 8.5 8.0 7.5 7.0 6.5 6.0 5.5 5.0 4.5 4.0 3.5 3.0 2.5 2.0 1.5 1.0 0.5 0.0
f1 (ppm)

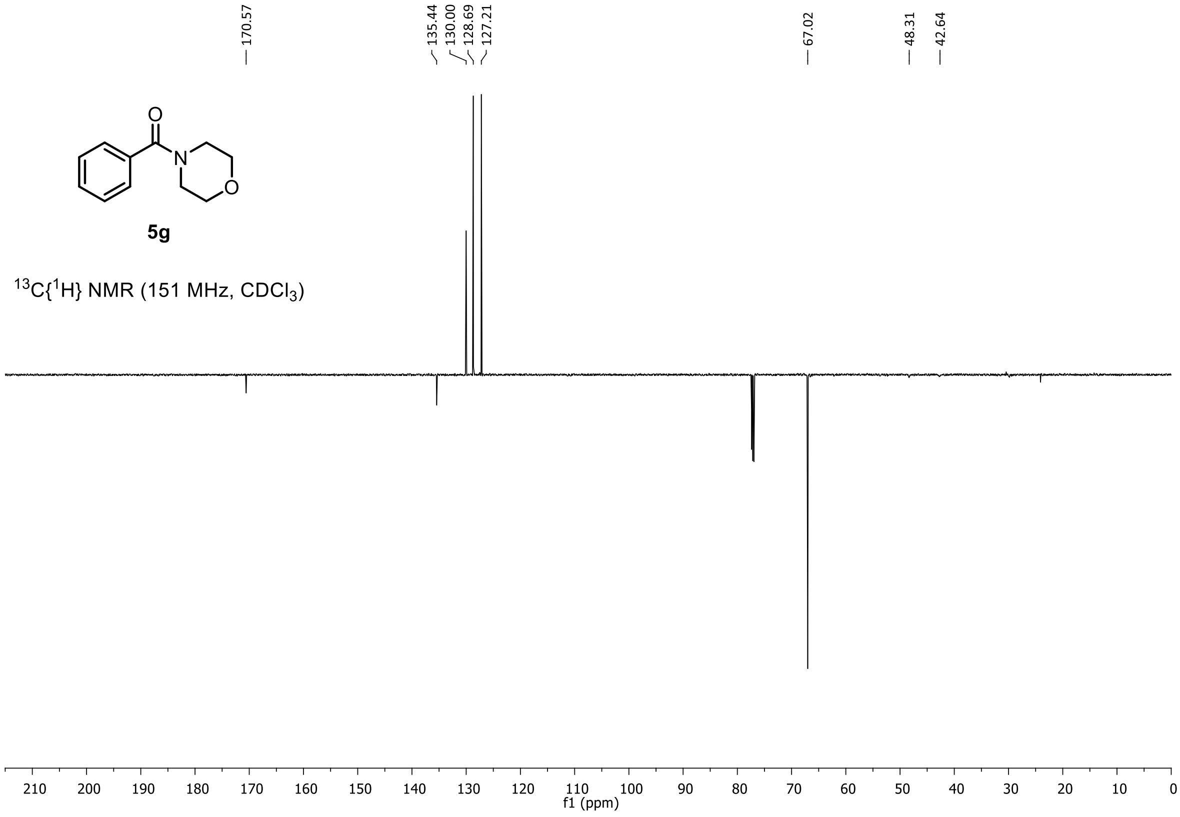
170.57
135.44
130.00
128.69
127.21
67.02
48.31
42.64
5g
$^{13}C\{^1H\}$ NMR (151 MHz, $CDCl_3$)
210 200 190 180 170 160 150 140 130 120 110 100 90 80 70 60 50 40 30 20 10 0
f1 (ppm)

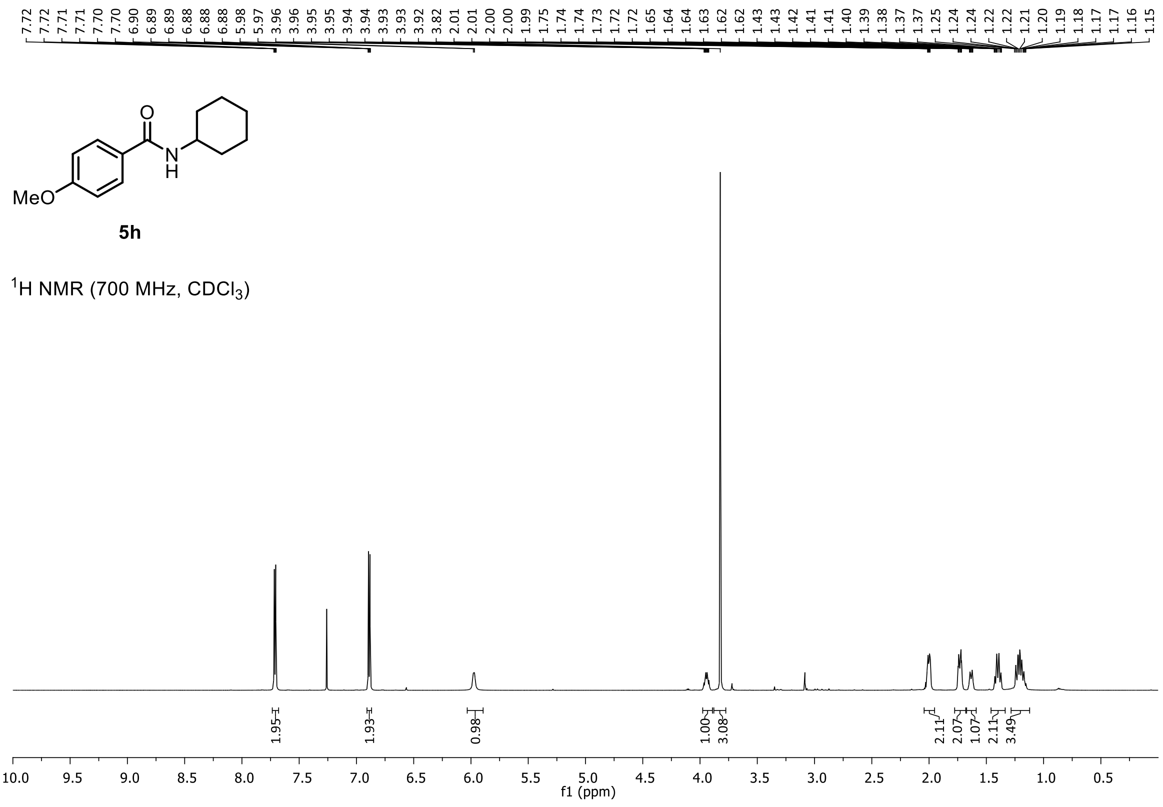
5h
^{1}H NMR (700 MHz, CDCl$_3$)
f1 (ppm)

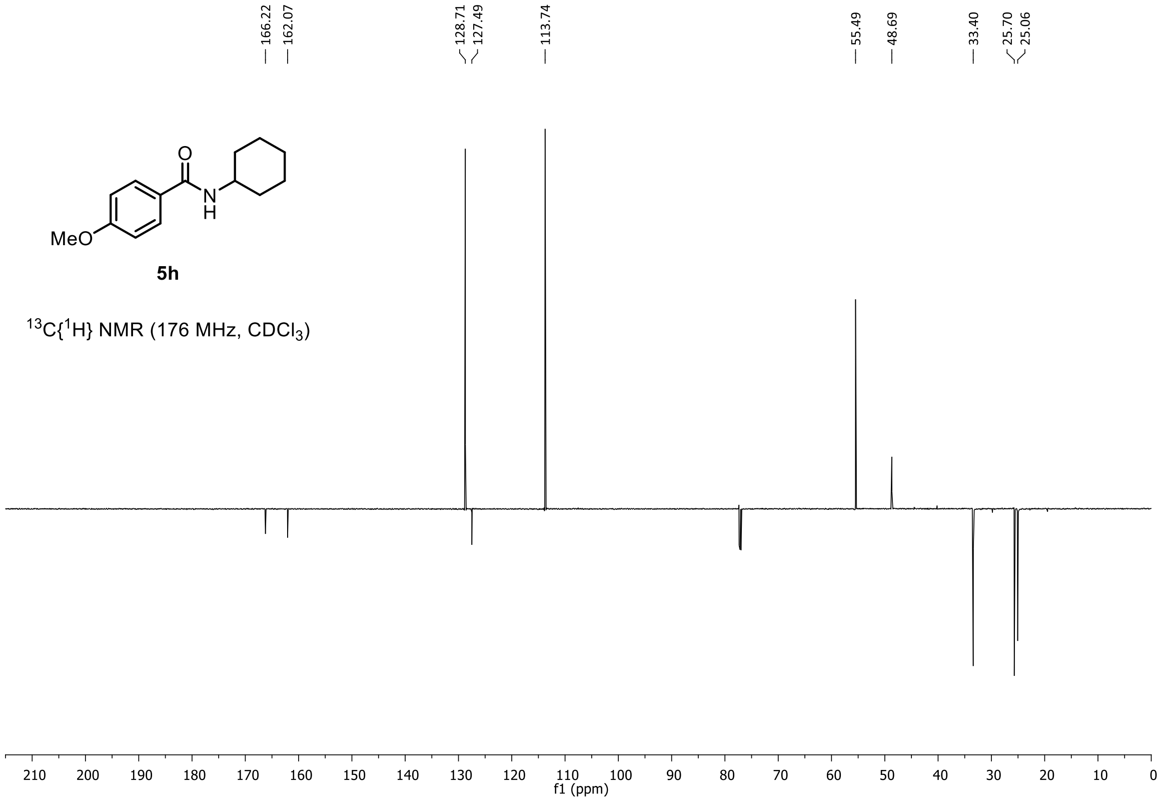
5h
^{13}C{^{1}H} NMR (176 MHz, CDCl$_3$)
f1 (ppm)

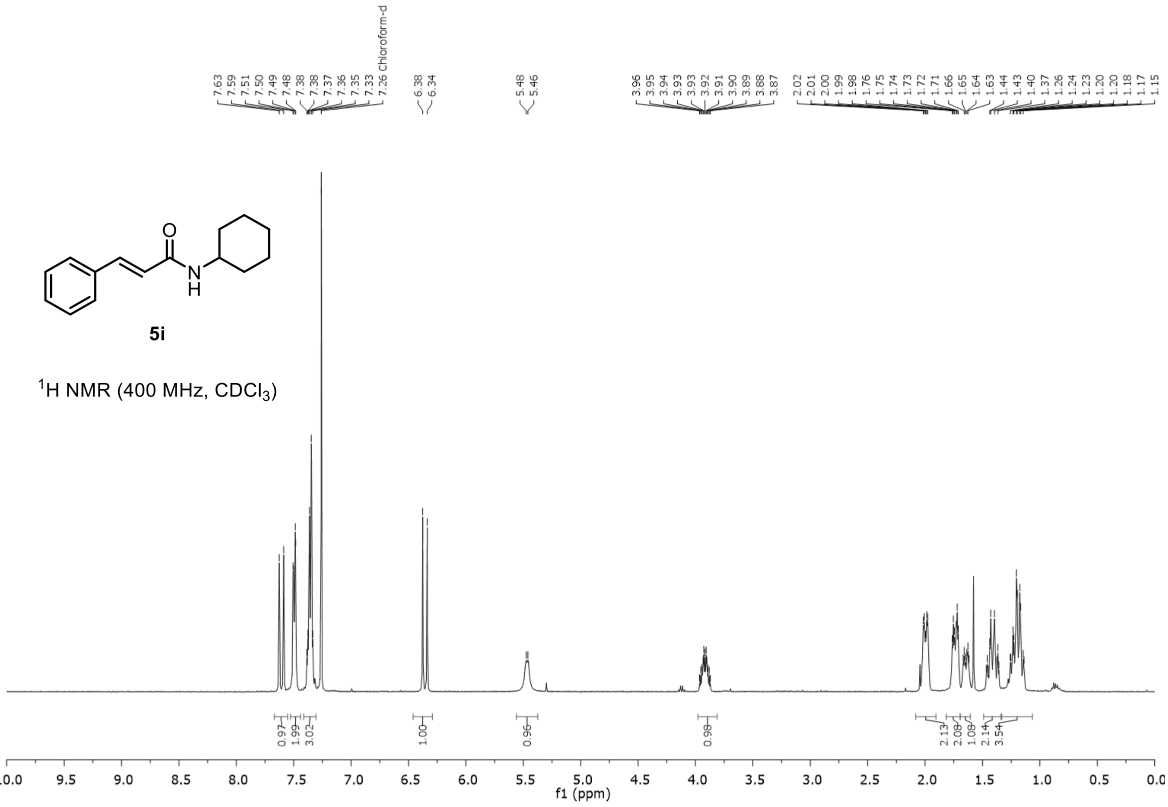
5i
^{1}H NMR (400 MHz, CDCl$_3$)
7.63 7.59 7.51 7.50 7.49 7.48 7.38 7.38 7.37 7.36 7.35 7.33 7.26 Chloroform-d
6.38 6.34
5.48 5.46
3.96 3.95 3.94 3.93 3.93 3.92 3.91 3.90 3.89 3.88 3.87
2.02 2.01 2.00 1.99 1.98 1.76 1.75 1.74 1.73 1.72 1.71 1.66 1.65 1.64 1.63 1.44 1.43 1.40 1.37 1.26 1.24 1.23 1.20 1.20 1.18 1.17 1.15
0.97 1.99 3.02 1.00 0.96 0.98 2.13 2.09 1.08 2.14 3.54
f1 (ppm)

4. References

(1) Grant, P. S.; Stopka, T.; Sabbatani, J.; Meyrelles, R.; Preinfalk, A.; Matyasovsky, J.; Maryasin, B.; Gonz, L.; Maulide, N. Direct Stereodivergent Olefination of Carbonyl Compounds with Sulfur Ylides. *J. Am. Soc.* **2022**, *144*, 12536–12543.

(2) Merad, J.; Matyašovský, J.; Stopka, T.; Brutiu, B. R.; Pinto, A.; Drescher, M.; Maulide, N. Stable and Easily Available Sulfide Surrogates Allow a Stereoselective Activation of Alcohols. *Chem. Sci.* **2021**, *12*, 7770–7774.

(3) Yamada, H.; Kinoshita, H.; Inomata, K.; Kotake, H. An Odorless Preparative Method of Sulfides and Thiocarboxylic S-Esters Using 3-(Alkylthio)-1,2-benzisothiazole 1,1-Dioxide. *Bull. Chem. Soc. Jpn*. 1983, *56*, 949–950.

(4) Leace, D. M.; Straub, M. R.; Matz, B. A.; Birman, V. B. Organocatalyzed Rearrangement of S-(2-Oxoalkyl)-thioenoates. *J. Org. Chem.* **2019**, *84*, 7523–7531.

(5) Yadav, D. K. T.; Bhanage, B. M. *tert*-Butyl Peroxybenzoate Mediated Selective and Mild N-Benzoylation of Ammonia/Amines under Catalyst- and Solvent-Free Conditions. *Synlett* **2015**, *26*, 1862–1866.

(6) Alalla, A.; Merabet-Khelassi, M.; Aribi-Zouioueche, L.; Riant, O. Green Synthesis of Benzamides in Solvent- and Activation-Free Conditions. *Synth. Commun.* **2014**, *44*, 2364–2376.

(7) Krieg, S. C.; Grimmer, J.; Kramer, P.; Bolte, M.; Kelm, H.; Manolikakes, G. Oxyenamides as Versatile Building Blocks for a Highly Stereoselective One-Pot Synthesis of the 1,3-Diamino-2-ol-Scaffold Containing Three Continuous Stereocenters. *Angew. Chemie Int. Ed.* **2021**, *60*, 23667–23671.

(8) Gockel, S. N.; Hull, K. L. Chloroform as a Carbon Monoxide Precursor: In or Ex Situ Generation of CO for Pd-Catalyzed Aminocarbonylations. *Org. Lett.* **2015**, *17*, 3236–3239.

(9) Zhao, Q.; Li, H.; Wang, L. The Direct Amidation of α-Diketones with Amines via TBHP-Promoted Oxidative Cleavage of C(sp^2)-C(sp^2) Bonds. *Org. Biomol. Chem.* **2013**, *11*, 6772–

6779.

(10) Loni, M.; Balmohammadi, Y.; Dadgar Yeganeh, R.; Imani, K.; Notash, B.; Bazgir, A. A Case Study of Pd⋯Pd Intramolecular Interaction in a Benzothiazole Based Palladacycle; Catalytic Activity toward Amide Synthesisviaan Isocyanide Insertion Pathway. *New J. Chem.* **2021**, *45*, 3290–3297.

(11) Townsend, S. D.; Wu, X.; Danishefsky, S. J. Enhancing the Scope of the Diels–Alder Reaction through Isonitrile Chemistry: Emergence of a New Class of Acyl-Activated Dienophiles. *J. Am. Chem. Soc.* **2012**, *134*, 10659–10663.
